# Meta-analyses on prevalence of selected Parkinson’s nonmotor symptoms before and after diagnosis

**DOI:** 10.1186/2047-9158-4-1

**Published:** 2015-01-08

**Authors:** Honglei Chen, Edward J Zhao, Wen Zhang, Yi Lu, Rui Liu, Xuemei Huang, Anna J Ciesielski-Jones, Michele A Justice, Deborah S Cousins, Shyamal Peddada

**Affiliations:** Epidemiology Branch, National Institute of Environmental Health Sciences, 111 T.W. Alexander Dr, P.O. Box 12233, Mail drop A3-05, Research Triangle Park, NC 27709 USA; Social & Scientific Systems, Inc, Durham, North Carolina USA; Departments of Neurology, Radiology, Neurosurgery, Pharmacology, & Kinesiology, Pennsylvania State University-Milton S. Hershey Medical Center, Hershey, Pennsylvania USA; Biostatistics Branch, National Institute of Environmental Health Sciences, Research Triangle Park, NC USA

**Keywords:** Parkinson’s disease, Nonmotor symptoms, Meta-analysis, Prevalence, Natural history

## Abstract

**Background:**

Nonmotor symptoms are common among patients with Parkinson’s disease (PD) and some may precede disease diagnosis.

**Methods:**

We conducted a meta-analysis on the prevalence of selected nonmotor symptoms before and after PD diagnosis, using random-effect models. We searched PubMed (1965 through October/November 2012) for the following symptoms: hyposmia, constipation, rapid eye movement sleep behavior disorder, excessive daytime sleepiness, depression, and anxiety. Eligible studies were publications in English with original data on one or more of these symptoms.

**Results:**

The search generated 2,373 non-duplicated publications and 332 met the inclusion criteria, mostly (n = 320) on symptoms after PD diagnosis. For all symptoms, the prevalence was substantially higher in PD cases than in controls, each affecting over a third of the patients. Hyposmia was the most prevalent (75.5% in cases vs. 19.1% in controls), followed by constipation (50% vs. 17.7%), anxiety (39.9% vs. 19.1%), rapid eye movement sleep behavior disorder (37.0% vs. 7.0%), depression (36.6% vs. 14.9%), and excessive daytime sleepiness (33.9% vs. 10.5%). We observed substantial heterogeneities across studies and meta-regression analyses suggested that several factors might have contributed to this. However, the prevalence estimates were fairly robust in several sensitivity analyses. Only 20 studies had data on any symptoms prior to PD diagnosis, but still the analyses revealed higher prevalence in future PD cases than in controls.

**Conclusion:**

These symptoms are common among PD patients both before and after diagnosis. Further studies are needed to understand the natural history of nonmotor symptoms in PD and their etiological and clinical implications.

**Electronic supplementary material:**

The online version of this article (doi:10.1186/2047-9158-4-1) contains supplementary material, which is available to authorized users.

Parkinson disease (PD) is the second most prevalent neurodegenerative disease and affects over one percent of the elderly. In addition to motor dysfunction, PD patients also suffer from a variety of nonmotor symptoms (NMS), ranging from hyposmia and depression early-on to dementia and visual hallucination at later stages of the disease. Many of these symptoms adversely affect the quality of life of PD patients [1], and some may precede PD diagnosis by years [2]. These prodromal NMS include hyposmia [3], constipation [4–6], depression [7, 8], anxiety [7, 9], rapid eye movement sleep behavior disorder (RBD) [10, 11], and excessive daytime sleepiness (EDS) [12, 13]. The presence of these NMS in prodromal PD is consistent with the Braak hypothesis [14] which implies that they may occur as the result of underlying Lewy pathogenesis at olfactory bulb, enteric nerves, and lower brainstem, before invading substantia nigra. If this hypothesis were true, investigation of early NMS may not only improve PD clinical care, but also advance our knowledge of PD etiology and natural history [15]. Fundamental to this research is to understand the prevalence and incidence of each symptom at various stages of PD. Yet, reported prevalence of these symptoms in PD patients varies greatly across published reports. For example, the reported proportion of hyposmia among PD patients ranges from 27% [16] to 100% [17], and depression from 4% [18] to 80.5% [19]. We therefore conducted meta-analyses to estimate the prevalence of these NMS before and after PD diagnosis.

## Methods

### Literature search and data abstraction

In October and November 2012, NIEHS librarians conducted separate searches for publications on each of the following NMS of PD: hyposmia, constipation, anxiety, depression, EDS, and RBD. We selected these symptoms because of clear evidence that they could develop prior to PD diagnosis. The searches were primarily based on MeSH terms for individual symptoms supplemented with keyword searches in titles or abstracts specifically to identify papers that had not yet been indexed by PubMed. We limited searches to studies that were relevant to PD by applying the MeSH Term/Major paper to “Parkinson Disease” or searching for the keywords “Parkinson’s OR Parkinson OR Parkinsons”. For MeSH terms, we used “olfaction disorders” for hyposmia, “constipation” for constipation, “depressive disorder” or “depression” for depression, “anxiety” or “anxiety disorders” for anxiety, “disorders of excessive somnolence” for EDS, and “REM sleep behavior disorder” for RBD. For the keyword searches, we used “olfact*”, “hyposmia”, “anosmia” or “smell” for hyposmia; “constipat* or “bowel movement” for constipation; “depress*” for depression, “anxiet*” or “anxious*” for anxiety; “daytime sleepiness” for EDS; and “rapid eye movement”, “rapid-eye-movement”, “REM”, or “RBD” for RBD. In addition to these specific symptoms, we also searched for general terms of pre-motor symptoms, using “early diagnosis” as the MeSH term and “non-motor” or “nonmotor”, or “premotor” or “pre-motor” or “prodrom*” as keywords. The search was restricted to original publications on one or more of these NMS by limiting searches to “Humans” and “English language” and by excluding comments, case reports, editorials, reviews, or letters. These searches generated a total of 2,298 entries. These searches were further supplemented with 592 articles from a separate search conducted in June 2011. The 2011 search used a combination of MeSH terms and keywords for PD and a broader range of individual NMS. Combing these two searches, we identified a total of 2,890 references. After excluding 517 duplicates, 2,373 were left for review.

**Figure 1:**
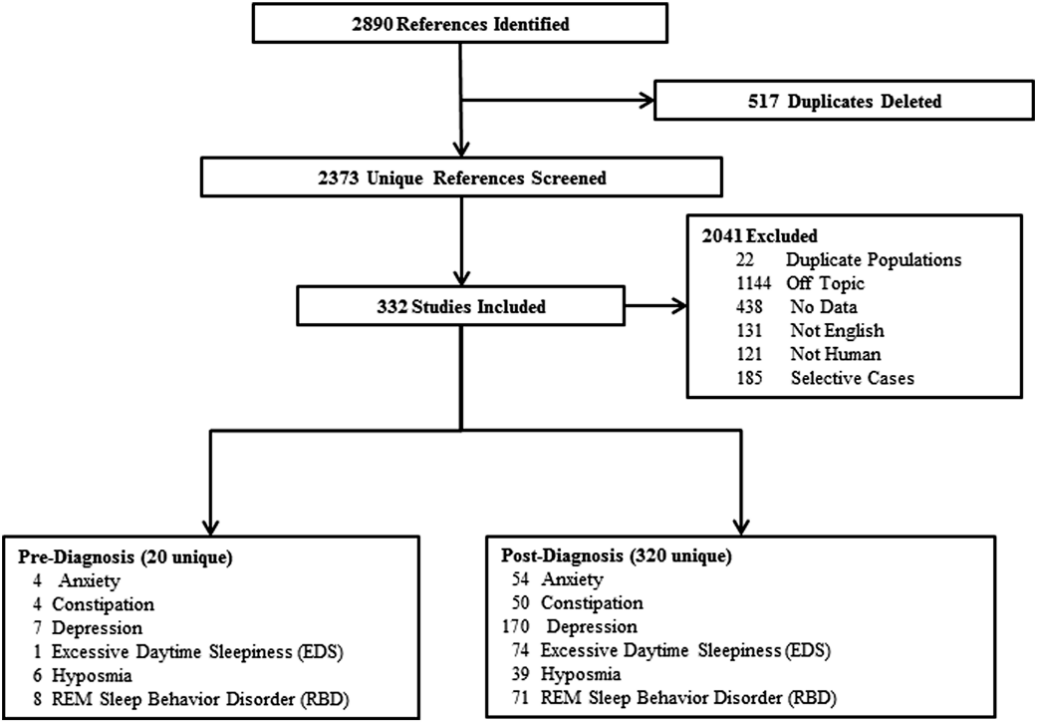
**Flow chart of literature review and data abstraction.**

Two authors independently screened the title and abstract of each paper and full text if necessary to determine study eligibility. Eligible studies are original publications that either reported prevalence of one or more NMS or provided original data from which prevalence could be calculated. Therefore, studies that only provided risk estimates such as relative risk without numbers of individuals with the symptoms were not eligible. We excluded studies for the following reasons: 1) duplicate populations (n = 22); 2) studies that did not have data on any of the above-referenced NMS (n = 1,144); 3) publications without original data, such as reviews, comments, editorials, or case-reports (n = 438); 4) non-English publications (n = 131); 5) non-human studies (n = 121); and 6) studies that could not be generalized to late-onset sporadic PD (n = 185). The full texts of 332 eligible studies were subsequently reviewed and relevant data were abstracted. For papers that reported data on more than one symptom, we abstracted data for each symptom separately. Similarly, for studies that reported data both after and prior to PD diagnosis, relevant data were abstracted separately. At each step, disagreements were resolved by group discussion and re-reviews. The details of the literature search are shown in Figure 1 and the characteristics of these studies are provided in Additional file 1: Table S1.

### NMS assessments

Details of symptom assessment in individual studies are provided in Additional file 1: Tables S2-S7. Assessment methods varied across studies and are summarized in Additional file 1: Table S8. Even for studies that used the same method, often different cutoffs were used. For the purpose of this meta-analysis, we refer NMS to symptoms rather than specific clinical diagnoses. Further, because we do not have original data of individual studies, we adopted symptom definitions that were used in individual studies.

### Statistical analyses

For the purpose of this meta-analysis, prevalence was defined as the proportion of individuals with a specific symptom among PD patients or among participants without PD (controls). As many clinical studies have a small sample size, rather than using the standard sample proportion to estimate prevalence, we used a Bayes estimator as commonly advocated for such situations [20]. In particular, we used the Laplace estimator [21] by adding one to the numerator (individuals with symptom) and two to the denominator (total number of participants) to all studies in order to obtain a more reasonable estimate in the meta-analyses. Such estimators of the binomial proportion are often used when dealing rare events to avoid zero estimates (with 0 standard error) for events that occur with small probabilities. Pooled prevalence estimates and standard errors were calculated using the random-effects model to account for between-study variations. Heterogeneity across studies was assessed using Cochran Q and I^2^ statistics [22]. For symptoms after diagnosis among PD cases, we further conducted sensitivity analyses to examine the robustness of our estimates by removing studies with fewer than 50 PD cases, studies without controls, or studies that simply used self-reported symptoms in NMS assessment. We assessed publication bias graphically using a funnel plot and quantitatively using the Begg rank correlation test and the Egger regression asymmetry test [23].

For symptoms after diagnosis, we further conducted meta-regression analyses among cases to examine factors that might have contributed to prevalence in individual studies. In these analyses, we performed logit transformation of the Laplace estimator since they are bounded between 0 and 1 and regression analysis requires unbounded support [21]. Due to the small number of studies, we did not conduct meta-regression analyses among controls or pre-diagnostic cases and controls. For the same reason, we did not evaluate publication bias for studies prior to PD diagnosis. Data preparation was performed using the SAS Software 9.3 (SAS Inc. Cary, NC) and statistical analyses were performed using STATA, version 11.1 (StataCorp, College Station, TX).

## Results

As shown in Figure 1, 320 studies reported data on symptoms after PD diagnosis; the exact number varies by symptoms from 39 for hyposmia to 170 for depression (Additional file 1: Table S1). Many studies provided data only on PD cases, and fewer included controls. For all symptoms, on average, approximately 60% of the cases were men and the average age at symptom evaluation was mid-60s, about seven years after diagnosis. Only 20 studies provided data on symptoms prior to PD diagnosis. Of these, several publications used data from large general purpose cohorts and therefore included few cases but a very large number of controls. Further, it is difficult to define the age or time of symptom assessment as many studies assessed cumulative prevalence – ever presence of symptoms prior to survey or disease diagnosis. Details of individual studies are summarized in Additional file 1: Tables S2-7.

### Measurements of NMS

A variety of methods were used in the assessment of NMS across studies and symptoms (Additional file 1: Table S8). Generally speaking, self-report was commonly used across symptoms. Structured questionnaires were most frequently used for assessing anxiety, depression, and EDS, while clinical examinations were used most often for RBD. For hyposmia, most likely an objective smell identification test was conducted.

### Prevalence of symptoms after diagnoses

**Figure 2:**
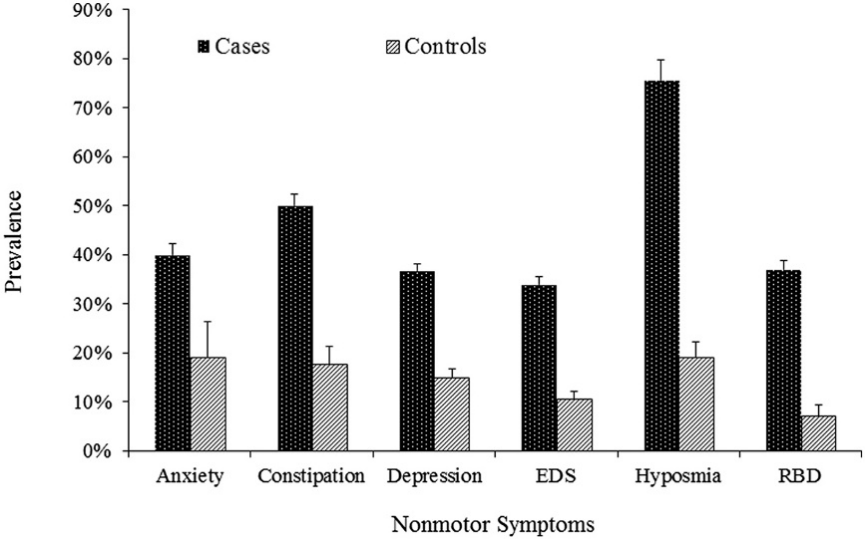
**Meta-analysis of the prevalence of selected non-motor symptoms after PD diagnosis.**

Each NMS affected at least a third of PD patients, 2.1 (anxiety) to 5.3 (RBD) times higher than the prevalence in controls. Hyposmia was the most prevalent in cases (75.5% vs. 19.1% in controls), followed by constipation (50.0% vs. 17.7%), anxiety (39.9% vs. 19.1%), RBD (37.0% vs. 7.0%), depression (36.6% vs. 14.9%), and EDS (33.9% vs. 10.5%). Similar results were obtained from all three sensitivity analyses (Table 1) (Figure 2).

**Table 1:** **Sensitivity analyses of the prevalence of selected non-motor symptoms of PD after diagnosis**

	PD cases								Controls					
	All data		Sample size ≥50		Studies with controls		Not self-reported single symptom		All data		Sample size ≥50		Not self-reported single symptom	
	N	%	N	%	N	%	N	%	N	%	N	%	N	%
Anxiety	54	39.9 ± 2.3	46	39.7 ± 2.5	5	33.9 ± 12.6	33	36.3 ± 2.4	5	19.1 ± 7.2	4	16.7 ± 7.7	2	17.3 ± 11.8
Constipation	50	50 ± 2.3	34	47.4 ± 2.5	14	44.8 ± 4.6	11	58.9 ± 5.8	14	17.7 ± 3.5	9	21 ± 4.2	3	16.7 ± 4.1
Depression	170	36.6 ± 1.4	136	35.7 ± 1.6	25	36.3 ± 3.7	145	35.6 ± 1.5	25	14.9 ± 1.8	20	15 ± 1.9	20	13.7 ± 1.8
EDS	74	33.9 ± 1.7	58	32.9 ± 1.9	22	32.8 ± 2.3	46	35.4 ± 1.8	22	10.5 ± 1.6	14	11.6 ± 2.1	17	9.2 ± 1.3
Hyposmia	39	75.5 ± 4.2	21	71 ± 5.9	17	76.5 ± 3.7	36	78.1 ± 2.7	17	19.1 ± 3.1	5	19.8 ± 5.5	16	20.1 ± 3.3
RBD	71	37 ± 1.8	51	36 ± 2	7	34.6 ± 8	53	39 ± 2.2	6	7 ± 2.4	3	7.4 ± 3.3	5	6.8 ± 3.4

There were however substantial heterogenities across studies in these analyses (cases: *I*^*2*^ = 92.5% to 94.4%, all P < 0.05; controls: *I*^*2*^ = 82.3% to 97.6%, all P <0.05). We therefore conducted meta-regression analyses among cases to evaluate potential sources of heterogenity (Table 2). Age at examination was not a major contributor to study heterogenity except for EDS, which was positively associated with age at examination. Disease duration was positively related to the prevalence of constipation and EDS across studies. Compared to studies using self-reported symptoms, studies with clinical examinations or structured questionnaires showed lower prevalence of anxiety. Studies using smell test showed much higher prevalence of hyposmia than self-reports. Compared to studies that were conducted in North America, studies carried out in other areas of the world showed higher prevalence of depression, and in Asia higher prevalence of constipation.

**Table 2:** **Patient characteristics and non-motor symptoms of PD meta-regression analysis among cases** ^**a**^

		Anxiety	Constipation	Depression	EDS	Hyposmia	RBD
		(N = 48)	(N = 39)	(N = 132)	(N = 63)	(N = 31)	(N = 58)
Age at examination		-0.0229428	0.0005514	-0.0123411	0.0664692^b^	0.0188255	0.0099428
Percent men		-2.136381	-0.2987655	0.3672128	1.512528	-1.614856	-0.4417779
Disease duration		-0.0117009	0.1112542^b^	0.0342322	0.0841153^b^	-0.0535196	-0.0164648
Symptom assessment (ref = Self-report symptom)^c^	Clinical exam/diagnosis	-0.7969928^b^	N/A	-0.2571586	N/A	N/A	0.4188744
	Structured questionnaire	-0.6081471^b^	0.4599401	0.0039112	0.0728903	N/A	0.5019682
	Smell test	N/A	N/A	N/A	N/A	1.580798^b^	N/A
Continent (ref = North America)	Europe	0.2150189	0.1268662	0.4494578^b^	-0.152538	0.1744257	-0.0748215
	Asia	-0.7000721	1.174114^b^	0.9805807^b^	-0.292603	-0.5923392	-0.3263885
	Other	0.051124	0.6902194	0.6824674^b^	0.2518004	-0.018769	-0.1487844

^a^Correlation coefficients were presented.

^b^P < 0.05.

^c^Methods with fewer than 5 studies were not included in the analyses.

Both visual inspections of the funnel plots and statistical tests (data not shown) suggest publication bias for studies that examined the prevalence of anxiety, contipation, depression, and EDS among cases after PD diagnosis, and for depression and hyposmia among controls (Egger test: P < 0.05). The positive Egger’s bias coefficients for these studies suggest that more extreme estimates from smaller studies were more likely to be published than less extreme estimates.

### Prevalence of symptoms prior to diagnoses

**Figure 3:**
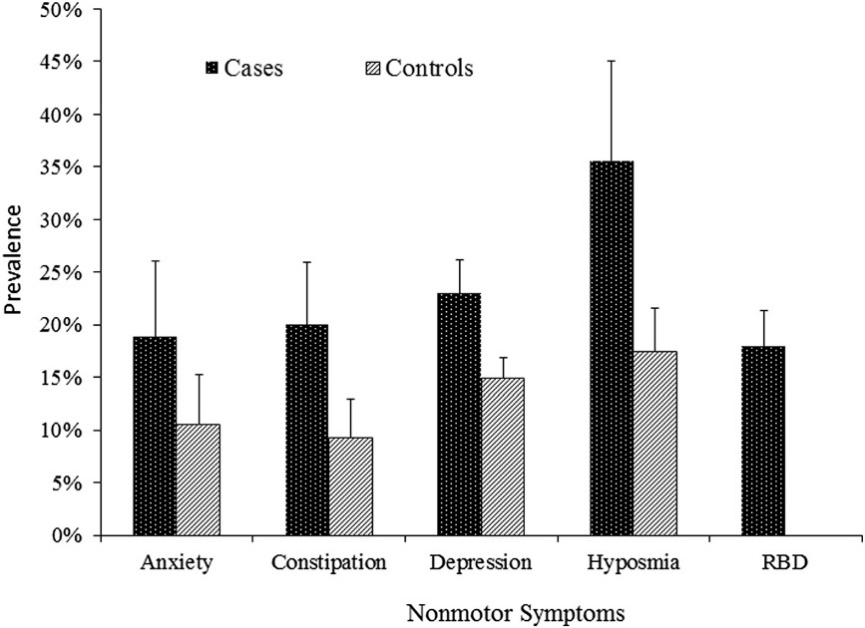
**Meta-analysis of the prevalence of selected non-motor symptoms prior to PD diagnosis.**

Few studies have data on NMS prior to PD diagnosis and most assessed symptoms via self-report. Despite these limitations, it is clear that the prevalence of these symptoms prior to PD diagnosis was higher among future PD cases than controls. The differences were, however, smaller than post-diagnosis data. Once again, hyposmia was the most prevalent, 35.5% in future cases versus 17.4% in controls, followed by depression (23.0% vs. 14.9%), constipation (20.0% vs. 9.3%), anxiety (18.8% vs. 10.5%), and RBD (17.9% for cases). Because only one study assessed EDS prior to PD diagnosis or RBD among controls, no summary estimates were calculated. Like the post-diagnosis analyses, significant study heterogeneities were observed across symptoms (cases: *I*^*2*^ = 79.1% to 96.4%, all P < 0.05; controls: *I*^*2*^ = 80.6% to 99.1%, P <0.01) (Figure 3).

## Discussion

It is well-known that NMS are common among PD patients; however, the exact prevalence and incidence are yet to be defined. To the best of our knowledge, this is the first comprehensive meta-analysis on the prevalence of multiple important NMS before and after PD diagnosis. A previous meta-analysis examined NMS and other factors in relation to PD risk [24]; however, it provided no data on symptom prevalence. The goal of our analysis was to survey available data and to provide preliminary prevalence estimates for future research on premotor symptoms of PD. As there are limited number of studies that were specifically designed to assess the prevalence of these symptoms, particularly prior to diagnosis, and among available studies, there are substantial heterogeneities in study population and symptom assessment, we have decided to be inclusive rather than exclusive in the selection of eligible studies.

Understanding the prevalence of NMS among PD patients is important in multiple ways. NMS adversely affect the quality of life of PD patients. Depression and sleep disturbances are often cited as the most important determinants of the health-related quality of life among PD patients [1, 25]. A good understanding of the prevalence of these symptoms allows for more comprehensive assessments of disease burden and better clinical managements. Some NMS, like the ones we analyzed, may precede PD diagnosis by years. Research on these symptoms may help us better define at-risk populations in our search for disease modifying interventions [26]. For example, several recent studies were carried out to examine whether hyposmia alone or with other NMS and neuro-biomarkers might help identify PD earlier than clinical diagnosis [27–29]. Equally important, this may also provide an unprecedented opportunity to investigate the origin and early etiology of PD as exemplified by the dual-hit hypothesis [30, 31]. These prodromal symptoms may serve as intermediate phenotypes to investigate risk factors that contribute to early PD development and to later conversion from intermediate phenotypes to clinical PD [15]. This research has been pioneered by experimental studies [31] and clinical investigations among RBD patients [32], and future population-based studies will be needed.

Our analyses systematically evaluated the prevalence of selected NMS before and after PD diagnosis. We clearly demonstrated that each of these symptoms affected at least a third of PD patients. Of these, hyposmia was the most prevalent, affecting 76% of PD patients after diagnosis and 36% prior to diagnosis. Although not directly comparable, all NMS in patients were about twice as prevalent after diagnosis as prior to diagnosis. This is consistent with the Braak hypothesis that the Lewy pathology is cumulative in PD [14] and also with the progressive nature of PD development. The prevalence of these symptoms was clearly higher in cases than in controls. After diagnosis, the largest difference was found for RBD, followed by hyposmia, EDS, and constipation, each about 3–5 times more prevalent among cases than among controls. Prior to diagnosis, the difference is however smaller, generally less than 2-fold. This, together with the relative high prevalence of symptoms among controls, supports the notion that most of these NMS are not specific; therefore future studies should focus on understanding patterns of multiple NMS and how they are related to future PD development [15, 33]. Notably, population data on prodromal PD are fairly limited and little data exist on the prevalence of EDS and RBD prior to PD diagnosis. Previous clinical-based studies suggest that RBD is the most specific prodromal symptom for PD [34], and this issue should be further evaluated in population-based studies.

It is not surprising that there are substantial heterogeneities across studies given the large variability in symptom definition, assessment methods, and patient characteristics. Many of the studies assessed symptoms via self-reports, which may be very useful to screen large populations cost-effectively but are not ideal for estimating prevalence. We found in the meta-regression analysis that self-reports might have underestimated the prevalence of hyposmia, but overestimated that for anxiety. This suggests that hyposmia are under-recognized by the elderly and objective means are needed to screen for smell deficit, such as the Sniffin’ Stick test [35] or the Brief Smell Identification Test [36]. Interestingly, with a few exceptions, we did not identify age and disease duration as major contributors to study heterogenity. This may relate to the fact that we analyzed aggregated data across studies, and this issue could be better investigated in large studies with indivdiual data.

In non-PD literature, prevalence data for the general population are widely available on depression, anxiety, and to a lesser extent, on constipation and hyposmia. Comparing our data directly with those is difficult, in part due to differences in study design, population characteristics, symptom assessment, and definitions of symptom prevalence. With these caveats, our data for controls are largely consistent with reports from several recent meta-analyses on constipation [37], depression [38], and anxiety [38] in general elderly populations. To the best of our knowledge, no other meta-analysis has been conducted on hyposmia. Our analyses showed that approximately 19% of the elderly population free of PD had hyposmia, which was also comparable to studies in the general older population [39]. While little data have been published on EDS and RBD outside the PD literature, our meta-analysis suggests that these symptoms may affect 7-10% of the elderly without PD.

Our analyses have several limitations. We limited our search to PubMed and publications in English and therefore might have missed a small number of relevant publications. Most studies were not originally designed to evaluate the prevalence of NMS, and there were substantial heterogeneities in the source population, study design, sample size, and symptom assessment. Nevertheless, we conducted several sensitivity analyses for symptoms after disease diagnosis and obtained similar results. Further, few studies have data prior to PD diagnosis, and therefore the corresponding estimates may not be as stable as those for post-diagnostic prevalence. Moreover, it is important to understand the temporal relationship of NMS to PD, particularly in the prodromal stage. However, nearly all studies had only cross-sectional measurements, and prospective investigations with repeated assessments are largely lacking. Finally, few previous studies examined the occurrence of multiple symptoms, which allows for a more comprehensive assessment of NMS burden among PD patients. Such assessment in the prodromal stage of PD may further help to better identify populations at risk despite that individual NMS are mostly not specific to PD [15].

Despite these limitations, our meta-analysis provided summary estimates of NMS among PD before and after diagnosis. Further prospective research is needed to examine the presence of multiple symptoms and their temporal relationships to PD to better understand the etiology and natural history of NMS and PD.

## Electronic supplementary material

Additional file 1: Table S1: Summary of study characteristics included in the meta-analyses. **Table S2.** Characteristics of individual studies on anxiety and PD. **Table S3.** Characteristics of individual studies on constipation and PD. **Table S4.** Characteristics of individual studies on depression and PD. **Table S5.** Characteristics of individual studies on excessive daytime sleepiness and PD. **Table S6.** Characteristics of individual studies on hyposmia and PD. **Table S7.** Characteristics of individual studies on REM sleep behavior disorder and PD. **Table S8.** Summary of methods of symptom assessments on selected nonmotor symptoms. **Table S9.** Abbreviations of assessment methods. (DOCX 207 KB)
