# Supplementary material for: Meta-analyses on prevalence of selected Parkinson’s nonmotor symptoms before and after diagnosis
Source: Transl Neurodegener. 2015 Jan 8;4(1):1. doi: 10.1186/2047-9158-4-1 (PMC4322463; doi:10.1186/2047-9158-4-1)
Supplement: Supplementary file 1 — Additional file 1: Table S1: Summary of study characteristics included in the meta-analyses. Table S2. Characteristics of individual studies on anxiety and PD. Table S3. Characteristics of individual studies on constipation and PD. Table S4. Characteristics of individual studies on depression and PD. Table S5. Characteristics of individual studies on excessive daytime sleepiness and PD. Table S6. Characteristics of individual studies on hyposmia and PD. Table S7. Characteristics of individual studies on REM sleep behavior disorder and PD. Table S8. Summary of methods of symptom assessments on selected nonmotor symptoms. Table S9. Abbreviations of assessment methods. (DOCX 207 KB) [file 40035_2014_74_MOESM1_ESM.docx]

| **Table S1. Summary of study characteristics included in the meta-analyses** | | | | | | | | | |  | | | |  |  |  | | |
| --- | --- | --- | --- | --- | --- | --- | --- | --- | --- | --- | --- | --- | --- | --- | --- | --- | --- | --- |
|  | Cases | | | | | | | | | Controls | | | | | | | | |
| Symptom | N of studies | Sample size^a^ | Age at Assessment | | Percent men | | Disease duration | | | N of studies | | | | Sample size | Age at Assessment | | | Percent men |
| ***After Diagnosis*** | | | | | | | | | | | | | | | | | | |
| Anxiety | 54 | 235±311 (113, 178) | | 65.9±4.3 (66.3, 4.2) | | 58.8±10 (59.6, 11.4) | | 6.9±3.1 (6.9, 2.7) | | | 5 | | | 134±93 (106, 77) | 65.6±3.4 (64.9, 4.5) | 48.8±9.9 (50.9, 14.5) | | |
| Constipation | 50 | 161±219 (88, 113) | | 66.5±3.8 (66.6, 3.8) | | 59.7±11.9 (60.4, 10.8) | | 6.3±3.6 (6.4, 3.8) | | | 14 | | | 96±80 (74, 118) | 65±2.9 (65.3, 4.7) | 52.4±13.5 (56.1, 10.8) | | |
| Depression | 170 | 210±350 (110, 180) | | 66.5±4.1 (67.1, 5.2) | | 59.2±10.2 (59.4, 12) | | 7±2.8 (7.2, 2.9) | | | 25 | | | 256±710 (100, 125) | 67.5±5.4 (67, 9.1) | 46±13.8 (47.8, 19.8) | | |
| EDS | 74 | 194±288 (94, 147) | | 66.5±4 (66.6, 4.9) | | 60.7±10.5 (60.8, 11.8) | | 7.5±2.8 (7.3, 2.9) | | | 22 | | | 92±69 (77, 79) | 66±6.1 (64.3, 9) | 52.5±16.2 (53.7, 15) | | |
| Hyposmia | 39 | 121±193 (58, 72) | | 64±4.8 (64.8, 7.4) | | 62.2±10.3 (61.1, 11.2) | | 6.5±2.9 (5.6, 2.6) | | | 17 | | | 50±45 (32, 25) | 63.9±5.2 (64.6, 8.6) | 44.5±10.2 (46.4, 17) | | |
| RBD | 71 | 143±202 (80, 82) | | 66.4±3.8 (67, 4.4) | | 61.4±11.2 (60.8, 10.8) | | 6.7±2.4 (6.4, 2.5) | | | 6 | | | 92±107 (42, 160) | 65.7±7.2 (63.2, 4.7) | 53±6.9 (53, 11.3) | | |
| ***Before Diagnosis*** | | | | | | | | | | | | | | | | | | |
| Anxiety | 4 | 157±45 (170, 57) | | 68±4 (68, 7) | | 58.1±6.1 (57.9, 10.4) | | | - | | | 3 | 6990±11856 (196, 20587) | | 65.8±6.3 (67.7, 12.2) | | 56.6±11.5 (62, 21.1) | |
| Constipation | 4 | 76±22 (77, 38) | | 67±1 (67, 1) | | 68.5±21.7 (61.1, 27.6) | | | - | | | 3 | 2282±3821 (93, 6636) | | 67.7 (67.7, 0) | | 57.3±10.3 (57.3, 14.5) | |
| Depression | 7 | 242±337 (164, 142) | | 69±5 (68, 4) | | 58.3±10.1 (57.7, 12.5) | | | - | | | 5 | 60204±123168 (196, 20587) | | 68.2±7 (69.4, 9.9) | | 54±10.9 (58.8, 18.6) | |
| EDS | 1 | 43 (43, 0) | | NA | | 100 (100, 0) | | | - | | | 1 | 3035 (3035, 0) | | 77.1 (77.1, 0) | | 100 (100, 0) | |
| Hyposmia | 6 | 46±30 (46, 41) | | 70±4 (68, 7) | | 68.8±18.8 (64.5, 12.3) | | | - | | | 4 | 935±1060 (718, 1720) | | 69.8±9 (67.7, 17.7) | | 67.9±22.2 (60.8, 28.8) | |
| RBD | 8 | 79±63 (60, 70) | | 68±3 (68, 4) | | 54.4±10.5 (57.7, 20.2) | | | - | | | 1 | 646 (646, 0) | | 74.8 (74.8, 0) | | 69.9 (69.9, 0) | |

^a^ mean ± standard deviation (median, IQR)

**Table S2. Study characteristics on anxiety among PD patients and controls after and prior to disease diagnosis**

|  | | | | Symptom Assessment Among Cases | | | | | |  | Symptom Assessment Among Controls | | | |
| --- | --- | --- | --- | --- | --- | --- | --- | --- | --- | --- | --- | --- | --- | --- |
| Authors, Year | Continent | Method |  | N | Mean Age at Assessment | Mean Duration (years) | Mean UPDRS-III | Men, % | Prevalence, % |  | N | Mean Age at Assessment | Men, % | Prevalence, % |
| **Studies with symptoms after PD diagnosis (N = 54)** | | | | | | | | | | | | | | |
| Anderson, K. E., 2002 | NA | BAI |  | 101 | NA | NA | NA | NA | 39 |  | NA | NA | NA | NA |
| Barone, P., 2009 | Europe | Self-report Symptom |  | 1072 | 67.4±9.4 | 5.1 | 24.2±13.1 | 60.4 | 55.8 |  | NA | NA | NA | NA |
| Brown, R. G., 2011 | Europe | HADS>=8 |  | 513 | 67.9±10.3 | 6.9±6.0 | 26.4±12 | 65.1 | 45 |  | NA | NA | NA | NA |
| Bugalho, P., 2012 | Europe | SCL-90-R>1 |  | 36 | 72.8±7.01 | 3.08±1.34 | NA | 47.2 | 27 |  | NA | NA | NA | NA |
| Chaudhuri, K. R., 2010 | Europe | NMSQ |  | 242 | 68.0±10.0 | 8.0±5.8 | NA | 63.2 | 41.7 |  | NA | NA | NA | NA |
| Chen, Y. K., 2010 | Asia | Clinical Exam |  | 133 | 66.3±11.2 | NA | NA | 55.6 | 27.1 |  | NA | NA | NA | NA |
| Cheon, S. M., 2008 | Asia | NMSQ |  | 74 | 64.9±8.6 | 6.4±6.1 | NA | 37.8 | 47.9 |  | NA | NA | NA | NA |
| Cheon, S. M., 2009 | Asia | Self-report Symptom |  | 67 | 58.2 | 9.3 | NA | 34.3 | 39.6 |  | NA | NA | NA | NA |
| Coelho, M., 2010 | Europe | Self-report Symptom |  | 50 | 74.1±7.0 | 17.94±6.3 | 49.18±13.1 | 46 | 50 |  | NA | NA | NA | NA |
| Di Giuda, D., 2012 | Europe | HAM-A>=11 |  | 21 | 59.6±13.4 | 4.0±2.1 | 16.1±6.2 | 66.7 | 81 |  | NA | NA | NA | NA |
| Dissanayaka, N. N., 2010 | Australia | Clinical Exam |  | 79 | 67.2±10.5 | 8.2±6.4 | 25.3±11.2 | 53.1 | 25 |  | NA | NA | NA | NA |
| Erro, R., 2012 | Europe | HADS-A>=7 |  | 34 | 59.3 | 1.3 | 13.88 | 64.7 | 26.5 |  | NA | NA | NA | NA |
| Erro, R., 2012 | Europe | NMSQ |  | 66 | 58.2±8.6 | 0.5 | 15.1±6.9 | 60.6 | 56.1 |  | NA | NA | NA | NA |
| Erro, R., 2012 | Europe | NMSQ |  | 97 | 57.6±8.5 | 1.14±0.48 | 16.2±7.4 | 60.8 | 54.9 |  | NA | NA | NA | NA |
| Evans, D., 2009 | Europe | HADS>=8 |  | 58 | 58.64 | 6.73 | NA | 48.3 | 57 |  | NA | NA | NA | NA |
| Gallagher, D. A., 2010 | Europe | NMSQ |  | 89 | 67.5±9.5 | 7.8±7.5 | 30.1±12.2 | 69 | 43 |  | NA | NA | NA | NA |
| Hanna, K. K., 2012 | North America | BAI>=18 |  | 38 | 62.1±8.7 | 8.4±6.4 | NA | 52.6 | 29 |  | NA | NA | NA | NA |
| Henderson, R., 1992 | North America | Zung Scale > 49 |  | 114 | 67.1±9.2 | 8.6±5.7 | NA | 53.7 | 15 |  | 106 | 64.4±10.0 | 35.3 | 5 |
| Hu, M., 2011 | Europe | HADS>=11 |  | 197 | 71.8 | 9.1 | NA | 65.8 | 31.3 |  | NA | NA | NA | NA |
| Hurt, C. S., 2012 | Europe | HADS>10 |  | 347 | 65.8±10.1 | 6.6±5.9 | NA | 64.6 | 23.3 |  | NA | NA | NA | NA |
| Kagi, G., 2010 | Europe | NMSQ |  | 27 | 68.2±5.9 | 13.3±3 | NA | 59.3 | 48.1 |  | NA | NA | NA | NA |
| Khedr, E. M., 2012 | Africa | NMSQ |  | 112 | 60.96±12.1 | 6.2±5.9 | NA | 68.8 | 60.7 |  | NA | NA | NA | NA |
| Kim, H. J., 2009 | Asia | NMSS |  | 23 | 64.3±8.5 | 0.8 | 16.5±9.0 | 47.8 | 39.1 |  | 23 | 65.3±9.1 | 47.8 | 30.4 |
| Kulisevsky, J., 2008 | Europe | HADS>=11 |  | 1351 | 70.61±9.09 | 5.65±4.94 | NA | 55.6 | 20.8 |  | NA | NA | NA | NA |
| Kummer, A., 2010 | Latin America | HAMA>= 14 |  | 91 | 57.2±10.5 | 8.8±4.9 | NA | 59.3 | 30.8 |  | NA | NA | NA | NA |
| Lee, M. A., 2007 | Europe | PACA |  | 123 | 75.4 | 6 | NA | 48 | 61.8 |  | 0 | NA | NA | NA |
| Leentjens, A. F., 2011 | International | Clinical Exam |  | 342 | 64.8±9.2 | 8.2±5.6 | 26.5±12.4 | 61 | 34.5 |  | NA | NA | NA | NA |
| Leiknes, I., 2010 | Europe | NPI |  | 189 | 67.8 | 2.3 | 23.2 | 59.8 | 15.3 |  | NA | NA | NA | NA |
| Leroi, I., 2012 | Europe | NPI |  | 127 | 65.4±11.6 | 7.8±5.3 | 27.33 | 66.9 | 40.6 |  | 33 | NA | NA | NA |
| Liu, A. A., 2011 | North America | BAI>=10 |  | 175 | 67 | 4.6 | 22.5 | 45.7 | 55.4 |  | NA | NA | NA | NA |
| Martinez-Martin, P., 2007 | International | NMSQ |  | 525 | 67.66±10.46 | 6.96±5.3 | NA | 58 | 45.3 |  | NA | NA | NA | NA |
| Martinez-Martin, P., 2011 | International | NMSS |  | 411 | 64.48±9.92 | 8.07±5.75 | NA | 61.3 | 50.6 |  | NA | NA | NA | NA |
| Martinez-Martin, P., 2012 | International | NMSS |  | 950 | 64.43±9.90 | 7.99±5.78 | NA | 62.6 | 46.4 |  | NA | NA | NA | NA |
| Matheson, S. F., 2012 | Australia | Clinical Exam |  | 58 | 66.24 | 8.15 | NA | 57 | 28 |  | NA | NA | NA | NA |
| Montel, S., 2009 | Europe | Clinical Exam |  | 135 | 60.6±10.5 | 9.5±4.9 | NA | 60 | 34 |  | NA | NA | NA | NA |
| Morley, J. F., 2011 | North America | SAI>55 |  | 248 | 64±10 | 6.6±5.4 | 22±10 | 75 | 16 |  | NA | NA | NA | NA |
| Nazzaro, J. M., 2011 | North America | NMSQ |  | 24 | 64.2±6.5 | 10.6±3.5 | 23.5±6.1 | 67 | 20.8 |  | NA | NA | NA | NA |
| Negre-Pages, L., 2010 | Europe | HADS>=8 |  | 422 | 68.6±9.8 | 5.4 | NA | 57 | 51 |  | 98 | 70.3±9.3 | 54 | 29 |
| Pontone, G. M., 2011 | North America | Clinical Exam |  | 249 | 66.3±10.3 | 8.3±6.4 | 18.1 | 66.7 | 42 |  | 0 | NA | NA | NA |
| Quelhas, R., 2009 | Europe | HADS>=8 |  | 43 | 72 | 7 | NA | 46 | 58 |  | NA | NA | NA | NA |
| Raggi, A., 2011 | Europe | NMSQ |  | 86 | 63.9±11.6 | NA | NA | 64 | 49 |  | NA | NA | NA | NA |
| Riedel, O., 2010 | Europe | NPI |  | 1449 | 70.7±8.4 | 5.8±5.2 | NA | 60.5 | 19.6 |  | 0 | NA | NA | NA |
| Rodriguez-Violante, M., 2011 | Latin America | NMSQ |  | 232 | 63.3±11.3 | 6.6±5.2 | NA | 56 | 44.8 |  | NA | NA | NA | NA |
| Romenets, S. R., 2012 | North America | HADS |  | 70 | 66.7±9.3 | 3.8±2.8 | 26.7±13.1 | 64.3 | 50.7 |  | NA | NA | NA | NA |
| Shulman, L. M., 2001 | North America | BAI>=10 |  | 99 | 67.4±8 | 6.9±5.7 | 22±9 | 53 | 33 |  | NA | NA | NA | NA |
| Shulman, L. M., 2002 | North America | BAI>=10 |  | 101 | 68.06±9.43 | 7.76±5.51 | 23.2±10.01 | 48 | 39 |  | NA | NA | NA | NA |
| Siri, C., 2010 | Europe | SCL-90R>1 |  | 486 | 65.82±10.25 | 12.06±5.93 | 20.66±11.3 | 58.6 | 46 |  | NA | NA | NA | NA |
| Skeie, G. O., 2012 | Europe | Self-report Symptom |  | 212 | NA | NA | NA | NA | 4.2 |  | 175 | NA | NA | NA |
| Solla, P., 2011 | Europe | Clinical Exam |  | 349 | 71.7±9.6 | 9.6±6.1 | 34.1±15.5 | 53 | 25.2 |  | NA | NA | NA | NA |
| Solla, P., 2012 | Europe | Clinical Exam |  | 156 | 69.3±8.5 | 6.3±4.4 | 30.2±15.3 | 58.3 | 23.1 |  | NA | NA | NA | NA |
| Spica, V., 2012 | Europe | NMSQ |  | 107 | 69.1±6.0 | 7.1±4.4 | 35.1±12.3 | 68.2 | 63.6 |  | NA | NA | NA | NA |
| Veazey, C., 2009 | North America | PRIME-MD |  | 80 | 70.8±8.6 | NA | NA | 98.8 | 62.5 |  | NA | NA | NA | NA |
| Wang, G., 2010 | Asia | NMSQ |  | 117 | 64.81±9.42 | 5.27±4.03 | NA | 64.1 | 27.9 |  | NA | NA | NA | NA |
| Yu, B., 2010 | Asia | NMSQ |  | 90 | 61.39±9.97 | NA | NA | 59 | 60 |  | 270 | 62.26±12.10 | 58 | 32.2 |
| Total Number of Cases and Controls |  |  |  | 12687 |  |  |  |  |  |  | 705 |  |  |  |
| **Studies with symptoms before PD diagnosis (N = 4)** | | | | | | | | | | | | | | |
| Gaenslen, A., 2011 | Europe | Self-report Symptom |  | 93 | 67.9±7.3 | NA | NA | 64.5 | 13.3 |  | 93 | 67.7±7.2 | 64.5 | 2.5 |
| Henderson, R., 1992 | North America | Self-report Symptom |  | 164 | NA | NA | NA | 53.7 | 9.5 |  | 150 | NA | 35.3 | NA |
| Ishihara-Paul, L., 2008 | Europe | Structured Questionnaire |  | 175 | 63.9±8.8 | NA | NA | 52 | 7.6 |  | 20680 | 58.8±9.3 | 43.4 | 3.8 |
| Shiba, M., 2000 | North America | Medical Records |  | 196 | 71 | NA | NA | 62 | 43.9 |  | 196 | 71 | 62 | 25.5 |
| Total Number of Cases and Controls |  |  |  | 628 |  |  |  |  |  |  | 21119 |  |  |  |

| **Table S3. Study characteristics on constipation among PD patients and controls after and prior to disease diagnosis** | | | | | | | | | | | | | | |
| --- | --- | --- | --- | --- | --- | --- | --- | --- | --- | --- | --- | --- | --- | --- |
|  |  |  |  |  |  |  |  |  |  |  |  |  |  |  |
|  | | | | Symptom Assessment Among Cases | | | | | |  | Symptom Assessment Among Controls | | | |
| Authors, Year | Continent | Method |  | N | Mean Age at Assessment | Mean Duration (years) | Mean UPDRS-III | Men, % | Prevalence, % |  | N | Mean Age at Assessment | Men, % | Prevalence, % |
| **Studies with symptoms after PD diagnosis (N = 50)** | | | | | | | | | | | | | | |
| Barone, P., 2009 | Europe | Self-report Symptom |  | 1072 | 67.4±9.4 | 5.1 | 24.2±13.1 | 60.4 | 27.5 |  | NA | NA | NA | NA |
| Bassotti, G., 2000 | Europe | <=1 BM per week |  | 31 | 73 | NA | NA | 61.2 | 71 |  | NA | NA | NA | NA |
| Byrne, K. G., 1994 | North America | Clinical Exam |  | 43 | 72.2 | 6.3 | NA | 62.8 | 76 |  | 0 | NA | NA | NA |
| Chaudhuri, K. R., 2006 | International | NMSQ |  | 123 | 68.1±10.3 | 6.4±4.3 | NA | 59.3 | 46.7 |  | 96 | 65.3±10.5 | 48 | 26 |
| Chaudhuri, K. R., 2010 | Europe | NMSQ |  | 242 | 68.0±10.0 | 8.0±5.8 | NA | 63.2 | 47.5 |  | NA | NA | NA | NA |
| Cheon, S. M., 2008 | Asia | NMSQ |  | 74 | 64.9±8.6 | 6.4±6.1 | NA | 37.8 | 65.8 |  | NA | NA | NA | NA |
| Coelho, M., 2010 | Europe | Self-report Symptom |  | 50 | 74.1±7.0 | 17.94±6.3 | 49.18±13.1 | 46 | 82 |  | NA | NA | NA | NA |
| Doi, H., 2012 | Asia | <3 BM per week |  | 31 | 68.1±7.8 | 4.2±3.8 | 18.37 | 35.5 | 64.5 |  | NA | NA | NA | NA |
| Eadie, M. J., 1965 | Australia | <1 BM per day |  | 76 | NA | NA | NA | NA | 60.5 |  | 96 | NA | NA | 12.5 |
| Edwards, L. L., 1991 | North America | <3 BM per week |  | 98 | 67.8 | 7.28 | NA | 60.2 | 28.7 |  | 50 | 63.3 | 24 | 10 |
| Edwards, L. L., 1993 | North America | <3 BM per week |  | 8 | 65.1 | 5.9 | 19.9 | 62.5 | 38 |  | 0 | NA | NA | NA |
| Edwards, L. L., 1994 | North America | <3 BM per week |  | 13 | 66.6 | 5.3 | 22.2 | 61.5 | 31 |  | 7 | 67 | 57.1 | 14 |
| Erro, R., 2012 | Europe | NMSQ |  | 66 | 58.2±8.6 | 0.5 | 15.1±6.9 | 60.6 | 22.7 |  | NA | NA | NA | NA |
| Erro, R., 2012 | Europe | NMSQ |  | 97 | 57.6±8.5 | 1.14±0.48 | 16.2±7.4 | 60.8 | 9.9 |  | NA | NA | NA | NA |
| Gabrielli, M., 2011 | Europe | ROME III Questionnaire |  | 48 | NA | 9.1 | 20.3 | 47.9 | 77.1 |  | 0 | NA | NA | NA |
| Gallagher, D. A., 2010 | Europe | NMSQ |  | 89 | 67.5±9.5 | 7.8±7.5 | 30.1±12.2 | 69 | 48 |  | NA | NA | NA | NA |
| Goldstein, D. S., 2010 | North America | N/A |  | 23 | 63.1 | NA | NA | 72.2 | 72 |  | 0 | NA | NA | NA |
| Jost, W. H., 1998 | Europe | <3 BM per week |  | 25 | 61.9±6.7 | 0.66 | NA | 56 | 28 |  | NA | NA | NA | NA |
| Kagi, G., 2010 | Europe | NMSQ |  | 27 | 68.2±5.9 | 13.3±3 | NA | 59.3 | 59.3 |  | NA | NA | NA | NA |
| Kaye, J., 2006 | Europe | Self-report Symptom |  | 156 | NA | NA | NA | NA | 59 |  | 148 | NA | NA | 20.9 |
| Khedr, E. M., 2012 | Africa | NMSQ |  | 112 | 60.96±12.1 | 6.2±5.9 | NA | 68.8 | 51.8 |  | NA | NA | NA | NA |
| Kim, H. J., 2009 | Asia | NMSS |  | 23 | 64.3±8.5 | 0.8 | 16.5±9.0 | 47.8 | 26.1 |  | 23 | 65.3±9.1 | 47.8 | 4.3 |
| Kim, J. S., 2012 | Asia | SCOPA-AUT |  | 47 | 66.6±11.7 | 1.8±1.6 | NA | 38.3 | 61.7 |  | NA | NA | NA | NA |
| Krogh, K., 2008 | Europe | <1 BM per 2 days |  | 416 | 69 | 7 | NA | 56 | 27 |  | 45 | NA | NA | 5 |
| Lee, M. A., 2007 | Europe | PACA |  | 123 | 75.4 | 6 | NA | 48 | 21.9 |  | 0 | NA | NA | NA |
| Martinez-Martin, P., 2007 | International | NMSQ |  | 525 | 67.66±10.46 | 6.96±5.3 | NA | 58 | 52.5 |  | NA | NA | NA | NA |
| Martinez-Martin, P., 2011 | International | NMSS |  | 411 | 64.48±9.92 | 8.07±5.75 | NA | 61.3 | 49.1 |  | NA | NA | NA | NA |
| Martinez-Martin, P., 2012 | International | NMSS |  | 950 | 64.43±9.90 | 7.99±5.78 | NA | 62.6 | 48 |  | NA | NA | NA | NA |
| Muller, B., 2011 | Europe | UPDRS |  | 207 | 67.9 | 2.3±1.8 | 23.2 | 58.9 | 39.3 |  | 175 | 67.5 | 59.4 | 13.8 |
| Nazzaro, J. M., 2011 | North America | NMSQ |  | 24 | 64.2±6.5 | 10.6±3.5 | 23.5±6.1 | 67 | 62.5 |  | NA | NA | NA | NA |
| Nihei, Y., 2012 | Asia | <1 BM per 3 days |  | 469 | 71±8.3 | 6.8±5.5 | 10.9±4.4 | 46.7 | 30.8 |  | NA | NA | NA | NA |
| Qin, Z., 2009 | Asia | <1 BM per day |  | 391 | 63.77±9.80 | 2.98±1.92 | 23.82±11.88 | 65.5 | 46.9 |  | NA | NA | NA | NA |
| Raggi, A., 2011 | Europe | NMSQ |  | 86 | 63.9±11.6 | NA | NA | 64 | 43.8 |  | NA | NA | NA | NA |
| Ramjit, A. L., 2010 | North America | Self-report Symptom |  | 58 | 69.27±6.85 | 10.96±8.66 | 30.76±10.57 | NA | 67.3 |  | 51 | 66.45±9.17 | NA | 21.6 |
| Rodriguez-Violante, M., 2011 | Latin America | NMSQ |  | 232 | 63.3±11.3 | 6.6±5.2 | NA | 56 | 57.7 |  | NA | NA | NA | NA |
| Romenets, S. R., 2012 | North America | SCOPA-AUT, Rome III questionnaire |  | 70 | 66.7±9.3 | 3.8±2.8 | 26.7±13.1 | 64.3 | 48.6 |  | NA | NA | NA | NA |
| Ruiz-Martinez, J., 2011 | Europe | <2 BM per week |  | 146 | 70.3±9.8 | 7.4±5.6 | 13.4±7.5 | 55.5 | 51.4 |  | NA | NA | NA | NA |
| Sakakibara, R., 2003 | Asia | Structured Questionnaire |  | 12 | 68 | 5 | NA | 83.3 | 83.3 |  | 10 | 62 | 70 | 20 |
| Savica, R., 2009 | North America | Medical Records |  | 196 | NA | NA | NA | 61.7 | 36.2 |  | 196 | NA | NA | 20.4 |
| Schrag, A., 2002 | Europe | Self-report Symptom |  | 124 | 72±10.9 | 6±4.6 | 22.7±11.1 | 52.3 | 52 |  | NA | NA | NA | NA |
| Singer, C., 1992 | North America | <1 BM per week |  | 48 | 65.8 | 8 | NA | 100 | 7.3 |  | 32 | 70.4 | NA | NA |
| Spica, V., 2012 | Europe | NMSQ |  | 107 | 69.1±6.0 | 7.1±4.4 | 35.1±12.3 | 68.2 | 43.9 |  | NA | NA | NA | NA |
| Sung, H. Y., 2012 | Asia | Self-report Symptom |  | 19 | 66.3±10.6 | 1.0±0.2 | NA | 42 | 57.9 |  | NA | NA | NA | NA |
| Tateno, F., 2011 | Asia | Structured Questionnaire |  | 19 | 66 | 2.2 | NA | 52.6 | 94.7 |  | NA | NA | NA | NA |
| Ueki, A., 2004 | Asia | <1 BM per 3 days |  | 94 | 68.1±8.6 | 8.6 | NA | 53.2 | 71.1 |  | NA | NA | NA | NA |
| Verbaan, D., 2007 | Europe | SCOPA-AUT |  | 420 | 61.1±11.5 | 10.5±6.5 | NA | 64 | 50 |  | 150 | 60.9±9.9 | 55 | 11 |
| Wang, G., 2010 | Asia | NMSQ |  | 117 | 64.81±9.42 | 5.27±4.03 | NA | 64.1 | 55.6 |  | NA | NA | NA | NA |
| Wang, S. J., 1993 | Asia | <3 BM per week or >25% straining |  | 62 | 65.6±7.3 | 4.7±3.6 | NA | 88.7 | 71 |  | 62 | NA | NA | NA |
| Witjas, T., 2002 | Europe | Self-report Symptom |  | 50 | 66.2±8.5 | 12.7±5.4 | 18.5 | 60 | 40 |  | 0 | NA | NA | NA |
| Yu, B., 2010 | Asia | NMSQ |  | 90 | 61.39±9.97 | NA | NA | 59 | 70 |  | 270 | 62.26±12.10 | 58 | 47.8 |
| Total Number of Cases and Controls |  |  |  | 8040 |  |  |  |  |  |  | 1411 |  |  |  |
| **Studies with symptoms before PD diagnosis (N = 4)** | | | | | | | | | | | | | | |
| Abbott, R. D., 2001 | North America | <1 BM per day |  | 96 | NA | NA | NA | 100 | 10.4 |  | 6694 | NA | NA | 4.2 |
| Chaudhuri, K. R., 2008 | International | Self-report Symptom |  | 54 | 66.7±10.04 | NA | NA | 57.7 | 39.6 |  | NA | NA | NA | NA |
| Gaenslen, A., 2011 | Europe | Self-report Symptom |  | 93 | 67.9±7.3 | NA | NA | 64.5 | 24.7 |  | 93 | 67.7±7.2 | 64.5 | 10.9 |
| Gonera, E. G., 1997 | Europe | Medical Records |  | 60 | NA | NA | NA | 51.7 | 6.7 |  | 58 | NA | 50 | 13.8 |
| Total Number of Cases and Controls |  |  |  | 303 |  |  |  |  |  |  | 6845 |  |  |  |

| **Table S4. Study characteristics on depression among PD patients and controls after and prior to disease diagnosis** | | | | | | | | | | | | | | |
| --- | --- | --- | --- | --- | --- | --- | --- | --- | --- | --- | --- | --- | --- | --- |
|  |  |  |  |  |  |  |  |  |  |  |  |  |  |  |
|  | | | | Symptom Assessment Among Cases | | | | | |  | Symptom Assessment Among Controls | | | |
| Authors, Year | Continent | Method |  | N | Mean Age at Assessment | Mean Duration (years) | Mean UPDRS-III | Men, % | Prevalence, % |  | N | Mean Age at Assessment | Men, % | Prevalence, % |
| **Studies with symptoms after PD diagnosis (N = 170)** | | | | | | | | | | | | | | |
| Andreadou, E., 2011 | Europe | Self-report Symptom |  | 139 | 69.6±9.1 | 8.5±6.2 | 24.9±14.3 | 48.9 | 29.7 |  | NA | NA | NA | NA |
| Araujo Lima, A. M., 2012 | Latin America | BDI>10 |  | 40 | 63.2±8.55 | 6.55±5.03 | 11.78 | 67.5 | 70 |  | NA | NA | NA | NA |
| Arun, M. P., 2011 | Asia | BDI>=14 |  | 46 | 65.5±9.4 | 4.3±3.5 | NA | 67.4 | 49.9 |  | 30 | 62.4±8.4 | 70 | 23.4 |
| Assogna, F., 2012 | Europe | Clinical Exam |  | 100 | 71.7±5.0 | 6.2±6.0 | 26.7±13.5 | 48 | 56 |  | 100 | 72.7±4.4 | 39 | 66 |
| Barone, P., 2009 | Europe | Self-report Symptom |  | 1072 | 67.4±9.4 | 5.1 | 24.2±13.1 | 60.4 | 22.4 |  | NA | NA | NA | NA |
| Becker, C., 2011 | Europe | Medical Records |  | 3637 | NA | NA | NA | NA | 6.5 |  | 3637 | NA | NA | 3.8 |
| Benito-Leon, J., 2009 | Europe | Self-report Symptom |  | 52 | 77.9±5.5 | NA | NA | 65.4 | 44.2 |  | 260 | 77.1±6.2 | 63.1 | 29.2 |
| Benkler, M., 2012 | Asia | N/A |  | 77 | 68.7±11.6 | 7±4.7 | 31±24.1 | 58.4 | 40 |  | 77 | NA | NA | NA |
| Boller, F., 1998 | Europe | Clinical Exam |  | 22 | 71±11.4 | NA | NA | 59.1 | 50 |  | 11 | 70.2±11.22 | 45.4 | NA |
| Bolluk, B., 2010 | Asia | Clinical Exam |  | 50 | 61.8±9.9 | NA | NA | 60 | 30 |  | 50 | 61.5±10.1 | 60 | 2 |
| Bouwmans, A. E., 2012 | Europe | HAM-D>=8 |  | 53 | 69.81±9.74 | NA | 16.88±6.06 | 63 | 19 |  | NA | NA | NA | NA |
| Brockmann, K., 2011 | Europe | BDI-II |  | 20 | 67.6±9.3 | 9.85±6.3 | 27.85±7.5 | NA | 25 |  | NA | NA | NA | NA |
| Brown, R. G., 2011 | Europe | HADS>=8 |  | 513 | 67.9±10.3 | 6.9±6.0 | 26.4±12 | 65.1 | 35 |  | NA | NA | NA | NA |
| Bryant, M. S., 2012 | North America | CES-D-10>=10 |  | 54 | 70.76 | 8.75 | 20.82 | 75.9 | 37 |  | NA | NA | NA | NA |
| Bugalho, P., 2012 | Europe | SCL-90-R>1 |  | 36 | 72.8±7.01 | 3.08±1.34 | NA | 47.2 | 36.1 |  | NA | NA | NA | NA |
| Butterfield, L. C., 2010 | North America | BDI-II>=10 |  | 68 | 69.96±7.03 | 7.07±4.96 | NA | 66.2 | 52 |  | NA | NA | NA | NA |
| Caap-Ahlgren, M., 2001 | Europe | GDS-15>=5 |  | 102 | 70.6 | NA | NA | 57 | 52 |  | NA | NA | NA | NA |
| Ceravolo, R., 2012 | Europe | Clinical Exam |  | 44 | 68.1±7.9 | 1.14±0.98 | 17.9±7.7 | NA | 13.6 |  | NA | NA | NA | NA |
| Chagas, M. H., 2010 | Latin America | DSM-IV |  | 78 | 61.03±10.52 | 8.22±4.98 | NA | 52.6 | 23.1 |  | NA | NA | NA | NA |
| Chagas, M. H., 2011 | Latin America | Clinical Exam |  | 110 | 61.09 | 7.67 | NA | 47.3 | 25.5 |  | NA | NA | NA | NA |
| Chaudhuri, K. R., 2010 | Europe | NMSQ |  | 242 | 68.0±10.0 | 8.0±5.8 | NA | 63.2 | 48.8 |  | NA | NA | NA | NA |
| Cheon, S. M., 2008 | Asia | NMSQ |  | 74 | 64.9±8.6 | 6.4±6.1 | NA | 37.8 | 65.3 |  | NA | NA | NA | NA |
| Cheon, S. M., 2009 | Asia | Self-report Symptom |  | 67 | 58.2 | 9.3 | NA | 34.3 | 16.7 |  | NA | NA | NA | NA |
| Cho, J. W., 2011 | Europe | HAM-D>=10 or BDI>=14 |  | 61 | 68.1 | NA | NA | 62.3 | 52.5 |  | 41 | 57.9±12.6 | 68.3 | NA |
| Cimino, C. R., 2011 | North America | HAM-D>8 |  | 50 | 67.12 | NA | NA | 64 | 42 |  | NA | NA | NA | NA |
| Coelho, M., 2010 | Europe | Clinical Exam |  | 50 | 74.1±7.0 | 17.94±6.3 | 49.18±13.1 | 46 | 62 |  | NA | NA | NA | NA |
| Cubo, E., 2012 | Europe | Clinical Exam |  | 557 | 68.8±9.7 | 1.3±0.6 | 24.97 | 60.3 | 44.8 |  | NA | NA | NA | NA |
| Defazio, G., 2008 | North America | BDI |  | 402 | 67.4±9.1 | 7.6 | 20.8±10.2 | 63.2 | 16.7 |  | 317 | 65.5±10.4 | 56.5 | 6 |
| Di Giuda, D., 2012 | Europe | HAM-D>=12 |  | 21 | 59.6±13.4 | 4.0±2.1 | 16.1±6.2 | 66.7 | 76.2 |  | NA | NA | NA | NA |
| Dias, F. M., 2011 | Latin America | Clinical Exam |  | 30 | 59.3±11.1 | 10 | NA | 33.3 | 56.6 |  | NA | NA | NA | NA |
| Dissanayaka, N. N., 2010 | Australia | Clinical Exam |  | 79 | 67.2±10.5 | 8.2±6.4 | 25.3±11.2 | 53.1 | 19 |  | NA | NA | NA | NA |
| Dissanayaka, N. N., 2011 | Australia | GDS-15, self-report symptom |  | 639 | NA | NA | NA | 63.7 | 66 |  | NA | NA | NA | NA |
| Djaldetti, R., 2008 | Asia | UPDRS |  | 159 | 68±10.2 | 8.2±7.3 | 34±19 | 61.6 | 37 |  | NA | NA | NA | NA |
| Dotchin, C. L., 2009 | Africa | HADS>=8 |  | 33 | 74.5 | 5 | NA | 69.7 | 52 |  | NA | NA | NA | NA |
| Drijgers, R. L., 2010 | Europe | HAM-D |  | 122 | 64.6±8.5 | 8.5±5.6 | 19.4±8 | 59 | 17.3 |  | NA | NA | NA | NA |
| Ehrt, U., 2010 | Europe | Clinical Exam |  | 235 | 74.7±8.4 | 9.2±5.8 | NA | 48.5 | 7.9 |  | NA | NA | NA | NA |
| Erro, R., 2012 | Europe | Clinical Exam |  | 66 | 58.2±8.6 | 0.5 | 15.1±6.9 | 60.6 | NA |  | NA | NA | NA | NA |
| Erro, R., 2012 | Europe | NMSQ |  | 97 | 57.6±8.5 | 1.14±0.48 | 16.2±7.4 | 60.8 | 43.9 |  | NA | NA | NA | NA |
| Evans, D., 2009 | Europe | HADS>=8 |  | 58 | 58.64 | 6.73 | NA | 48.3 | 50 |  | NA | NA | NA | NA |
| Factor, S. A., 2011 | North America | BDI-II>10 |  | 500 | 67.7±10.8 | 8.5±6.2 | NA | 62 | 34.6 |  | NA | NA | NA | NA |
| Farabaugh, A. H., 2011 | North America | HANDS>=6 |  | 158 | 66.8±9.6 | 9.1±6.2 | NA | 68 | 36.7 |  | NA | NA | NA | NA |
| Fernandez, H. H., 2009 | North America | BDI>=14 |  | 82 | 67.7±9.96 | 8.41±6.08 | 36.96±12.31 | 70.7 | 30 |  | NA | NA | NA | NA |
| Fujiwara, S., 2011 | Asia | SRQ-D>=11 |  | 100 | 69.3±8.41 | 6.74 | NA | 46 | 46 |  | 111 | 67±7.68 | 28.8 | 10.8 |
| Gallagher, D. A., 2010 | Europe | NMSQ |  | 94 | 67.5±9.5 | 7.8±7.5 | 30.1±12.2 | 69 | 48 |  | NA | NA | NA | NA |
| Gerbin, M., 2012 | North America | CES-D-10>=10 |  | 40 | 68.9±8.0 | 9.3±5.6 | NA | 65 | 31.5 |  | 120 | 70.3±6.0 | 36.7 | 22.2 |
| Ghys, L., 2011 | Europe | PDQ-8 |  | 267 | 64.6±9.7 | 4.8±4.4 | 30.4±12.9 | 65.5 | 35.6 |  | NA | NA | NA | NA |
| Giladi, N., 2000 | Asia | Clinical Exam |  | 172 | 70.3±11.7 | 11.8±5.6 | NA | 57.6 | 33 |  | NA | NA | NA | NA |
| Go, C. L., 2011 | North America | MADRS>=14 |  | 76 | 59.4 | 1.3 | 34.8 | 42 | 60.5 |  | NA | NA | NA | NA |
| Gomez-Esteban, J. C., 2009 | Latin America | Clinical Exam |  | 118 | 60.4±11.2 | 8.5±6.2 | 25.1±8.9 | 54.2 | 45.8 |  | NA | NA | NA | NA |
| Goulart, F. O., 2009 | Latin America | Clinical Exam |  | 50 | 70.5 | 6.3 | 31.5 | 44 | 44 |  | 50 | 73.9 | 14 | 8 |
| Gupta, A., 2000 | Asia | GDS>=11 |  | 40 | 63.75 | 5.75 | NA | 55 | 80.5 |  | NA | NA | NA | NA |
| Han, M., 2011 | Asia | BDI>=14 |  | 127 | 69.1 | 5.8 | NA | 44.9 | 43.3 |  | NA | NA | NA | NA |
| Hanna, K. K., 2012 | North America | BDI-II>=17 |  | 38 | 62.1±8.7 | 8.4±6.4 | NA | 52.6 | 21 |  | NA | NA | NA | NA |
| Happe, S., 2001 | Europe | ZDS>=35 |  | 56 | 69.9±9.2 | 8.6±5.6 | NA | 39.3 | 76.4 |  | 59 | 71.4±9.3 | 48.8 | 42.4 |
| Hatano, T., 2009 | Asia | Self-report Symptom |  | 132 | NA | 9.5 | NA | 52.3 | 48 |  | NA | NA | NA | NA |
| Havlikova, E., 2008 | Europe | HADS>=11 |  | 78 | 68.8±8.7 | 7.2±6.8 | NA | 52.6 | 14.3 |  | NA | NA | NA | NA |
| Henderson, J. M., 2003 | Australia | Self-report Symptom |  | 38 | 72±1 | 8.2±1 | NA | 45 | 16 |  | 32 | 74±2 | 34.3 | 6 |
| Henderson, R., 1992 | North America | MAACL DYS>2 SD above mean |  | 114 | 67.1±9.2 | 8.6±5.7 | NA | 53.7 | 23 |  | 106 | 64.4±10.0 | 35.3 | 17 |
| Herlofson, K., 2012 | Europe | MADRS>14 |  | 199 | 67.6±9.1 | NA | 22.9±11.1 | 58.8 | 4 |  | 172 | 67.4±9.1 | 59.3 | 0.6 |
| Herzallah, M. M., 2010 | Asia | BDI-II>13 |  | 12 | 60.28±9.19 | 5.5±4.64 | 24.2±13.98 | 75 | 58.3 |  | 12 | 58.43±6.86 | 37.5 | 16.7 |
| Hesse, S., 2009 | Europe | BDI |  | 140 | 64.6 | NA | 23.14 | 51.4 | 21.4 |  | 18 | 62.3±10.9 | 44 | NA |
| Hinnell, C., 2012 | Europe | HADS>=8 |  | 462 | 67.5±10.3 | 5.0±8.0 | 25.9±11.6 | 64.9 | 33.1 |  | NA | NA | NA | NA |
| Hu, M., 2011 | Europe | HADS>=11 |  | 201 | 71.8 | 9.1 | NA | 65.8 | 37.3 |  | NA | NA | NA | NA |
| Hurt, C. S., 2012 | Europe | HADS>=8 |  | 37 | 66.1±7.2 | 8.3±3.6 | 17.7 | 51 | 45.9 |  | NA | NA | NA | NA |
| Hurt, C. S., 2012 | Europe | HADS>10 |  | 347 | 65.8±10.1 | 6.6±5.9 | NA | 64.6 | 10.4 |  | NA | NA | NA | NA |
| Imamura, K., 2011 | Asia | Clinical Exam |  | 52 | 71.5±10.2 | 5.9 | NA | 55.8 | 57.7 |  | 9 | 72.7±7.0 | 55.6 | NA |
| Inoue, T., 2010 | Asia | BDI-II>=14 |  | 105 | 68.5 | 7.6 | NA | 55.2 | 38 |  | NA | NA | NA | NA |
| Jasinska-Myga, B., 2010 | North America | Clinical exam, antidepressant use |  | 685 | 70.8±9.1 | 6.3 | 30.2±14.6 | 68 | 18 |  | NA | NA | NA | NA |
| Johnson, D. K., 2011 | North America | N/A |  | 47 | 71.1±8.0 | NA | NA | 75 | 26.3 |  | 191 | 74.1±9.4 | 39 | 5.6 |
| Jones, C. A., 2009 | North America | CIDI-SFMD |  | 279 | 68.9 | 7.3 | NA | 55.9 | 9 |  | NA | NA | NA | NA |
| Joutsa, J., 2012 | Europe | BDI>=10 |  | 575 | 64 | 6 | NA | 63.5 | 47.7 |  | NA | NA | NA | NA |
| Kagi, G., 2010 | Europe | NMSQ |  | 27 | 68.2±5.9 | 13.3±3 | NA | 59.3 | 63 |  | NA | NA | NA | NA |
| Kasten, M., 2012 | Europe | BDI>=9 |  | 90 | 68±7 | 6±5 | 24.2±9.0 | 66 | 52 |  | 127 | 59±12 | 52 | 18 |
| Khedr, E. M., 2012 | Africa | NMSQ |  | 112 | 60.96±12.1 | 6.2±5.9 | NA | 68.8 | 47.3 |  | NA | NA | NA | NA |
| Kim, H. J., 2009 | Asia | NMSS |  | 23 | 64.3±8.5 | 0.8 | 16.5±9.0 | 47.8 | 34.8 |  | 23 | 65.3±9.1 | 47.8 | 8.7 |
| Kim, J. S., 2012 | Asia | Clinical Exam |  | 47 | 66.6±11.7 | 1.8±1.6 | NA | 38.3 | 59.6 |  | NA | NA | NA | NA |
| Kirsch-Darrow, L., 2011 | North America | BDI-II>=14 |  | 161 | 64.1±9.7 | 8.5 | 25.5±8.6 | 68.9 | 25.3 |  | NA | NA | NA | NA |
| Klotsche, J., 2011 | Europe | BDI |  | 145 | 67.3±9.6 | 9.3±7.4 | 31.2±21.6 | 66.9 | 44.3 |  | NA | NA | NA | NA |
| Koerts, J., 2012 | Europe | MADRS>=18 |  | 43 | 63.7±8.6 | 5.1±4.1 | 24.6±8.8 | 56 | 18.6 |  | 25 | 62.8±11.5 | 44 | NA |
| Kostic, V. S., 1991 | North America | Clinical Exam |  | 26 | 56.42 | NA | NA | 57.7 | 30.8 |  | NA | NA | NA | NA |
| Kulisevsky, J., 2008 | Europe | HADS>=8 |  | 1351 | 70.61±9.09 | 5.65±4.94 | NA | 55.6 | 49.7 |  | NA | NA | NA | NA |
| Kummer, A., 2009 | Latin America | Clinical Exam |  | 82 | 57.2±9.8 | 8.5±4.6 | NA | 61.2 | 41.5 |  | NA | NA | NA | NA |
| Kummer, A., 2010 | Latin America | Clinical Exam |  | 91 | 57.2±10.5 | 8.8±4.9 | NA | 59.3 | 45.1 |  | NA | NA | NA | NA |
| Larsen, J. P., 2000 | Europe | BDI |  | 240 | 73.6±8.4 | 9.1±5.8 | 28.33 | 49.7 | 21.1 |  | 100 | NA | NA | 3 |
| Lavault, S., 2010 | Europe | BDI>13 |  | 61 | 64.3 | 6.89 | 18.9 | 64 | 36 |  | NA | NA | NA | NA |
| Lee, M. A., 2007 | Europe | BDI>=17 |  | 123 | 75.4 | 6 | NA | 48 | 16.3 |  | 0 | NA | NA | NA |
| Leentjens, A. F., 2011 | International | Clinical Exam |  | 342 | 64.8±9.2 | 8.2±5.6 | 26.5±12.4 | 61 | 19.8 |  | NA | NA | NA | NA |
| Leiknes, I., 2010 | Europe | NPI |  | 189 | 67.8 | 2.3 | 23.2 | 59.8 | 36 |  | NA | NA | NA | NA |
| Leroi, I., 2012 | Europe | NPI |  | 127 | 65.4±11.6 | 7.8±5.3 | 27.33 | 66.9 | 38.3 |  | 33 | NA | NA | NA |
| Letro, G. H., 2009 | Latin America | BDI |  | 50 | 62.9 | 8.06±4.77 | NA | 60 | 44 |  | NA | NA | NA | NA |
| Li, W., 2010 | Asia | Clinical Exam |  | 32 | 62.9 | 5.94 | 36.11 | 43.8 | 43.8 |  | NA | NA | NA | NA |
| Liu, A. A., 2011 | North America | BDI>=10 |  | 175 | 67 | 4.6 | 22.5 | 45.7 | 45.7 |  | NA | NA | NA | NA |
| Lo, R. Y., 2009 | North America | UPDRS |  | 573 | 70.5±7.4 | NA | NA | 61.3 | 12.4 |  | NA | NA | NA | NA |
| Margis, R., 2010 | Latin America | GDS>=10 |  | 57 | 70.3±6.8 | 7.5±5.8 | 28.5±16.2 | 53 | 49.1 |  | NA | NA | NA | NA |
| Martinez-Martin, P., 2007 | International | NMSQ |  | 525 | 67.66±10.46 | 6.96±5.3 | NA | 58 | 50.1 |  | NA | NA | NA | NA |
| Martinez-Martin, P., 2011 | International | NMSS |  | 411 | 64.48±9.92 | 8.07±5.75 | NA | 61.3 | 49.6 |  | NA | NA | NA | NA |
| Martinez-Martin, P., 2012 | International | NMSS |  | 950 | 64.43±9.90 | 7.99±5.78 | NA | 62.6 | 47.6 |  | NA | NA | NA | NA |
| Matheson, S. F., 2012 | Australia | Clinical Exam |  | 58 | 66.24 | 8.15 | NA | 57 | 21 |  | NA | NA | NA | NA |
| Matuja, W. B., 2008 | Africa | BDI>=10 |  | 42 | 61.5 | NA | NA | 59.5 | NA |  | NA | NA | NA | NA |
| Miwa, H., 2011 | Asia | SDS>=40 |  | 46 | 68.6±9.5 | 5.4 | 23.51 | 45.6 | 48 |  | NA | NA | NA | NA |
| Montel, S., 2009 | Europe | Clinical Exam |  | 135 | 60.6±10.5 | 9.5±4.9 | NA | 60 | 43 |  | NA | NA | NA | NA |
| Morley, J. F., 2011 | North America | GDS-15>=5 |  | 248 | 64±10 | 6.6±5.4 | 22±10 | 75 | 33 |  | NA | NA | NA | NA |
| Nation, D. A., 2009 | North America | BDI |  | 111 | 64.1±9.7 | 11.8±5.5 | NA | 69.3 | 26.1 |  | NA | NA | NA | NA |
| Negre-Pages, L., 2010 | Europe | HADS>=8 |  | 422 | 68.6±9.8 | 5.4 | NA | 57 | 40 |  | 98 | 70.3±9.3 | 54 | 10 |
| O'Sullivan, S. S., 2008 | Europe | Medical Records |  | 433 | 62.9 | 2 | NA | 63 | 24.7 |  | NA | NA | NA | NA |
| Oguru, M., 2010 | Asia | BDI-II>=14 |  | 150 | 69.7±8.6 | 6.3±4.4 | NA | 46.7 | 56 |  | NA | NA | NA | NA |
| Ondo, W. G., 2011 | North America | N/A |  | 40 | 69.1±7.8 | NA | 21.65 | 60 | 40 |  | NA | NA | NA | NA |
| Pedersen, K. F., 2009 | Europe | Clinical exam, MADRS |  | 79 | 72±8.3 | 13±4.7 | 28.5±17.6 | 44.3 | 20.3 |  | NA | NA | NA | NA |
| Pedersen, K. F., 2010 | Europe | MADRS>17 or minor depression |  | 175 | 67.8±9.0 | 2.3±1.8 | 23.6±11.5 | 58.3 | 16.6 |  | 165 | 67.2±9.1 | 59.4 | NA |
| Peralta, C. M., 2009 | Europe | Clinical Exam |  | 113 | 67.4±9.2 | 9.4±5.6 | 28.9±12.1 | 72.6 | 54.9 |  | NA | NA | NA | NA |
| Phuong, L., 2009 | North America | Clinical Exam |  | 193 | 65.8±10.7 | 7.3±5.5 | 21.9±10.8 | 79.3 | 33.2 |  | NA | NA | NA | NA |
| Piccinni, A., 2012 | Europe | Clinical Exam |  | 122 | 67.01±8.5 | 6.9±4.8 | 23.01 | 57.4 | 52.2 |  | NA | NA | NA | NA |
| Pollak, L., 2009 | Asia | Clinical Exam |  | 54 | 66±10.1 | 7.4±5.6 | NA | 59.2 | 42.6 |  | 53 | 46±15 | 45.7 | NA |
| Qin, Z., 2009 | Asia | CES-D>=16 |  | 391 | 63.77±9.8 | 2.98±1.92 | 23.82±11.84 | 65.5 | 37.3 |  | NA | NA | NA | NA |
| Quelhas, R., 2009 | Europe | HADS>=8 |  | 43 | 72 | 7 | NA | 46 | 58 |  | NA | NA | NA | NA |
| Raggi, A., 2011 | Europe | NMSQ |  | 86 | 63.9±11.6 | NA | NA | 64 | 57.3 |  | NA | NA | NA | NA |
| Ravina, B., 2007 | North America | GDS15>=5 |  | 413 | 61.7 | 0.66 | 15.9 | 64.2 | 13.8 |  | NA | NA | NA | NA |
| Ravina, B., 2009 | North America | GDS-15>=5 |  | 537 | 59.8±9.8 | 2.1±1.4 | 17.9±8.0 | 65 | 14 |  | NA | NA | NA | NA |
| Ready, R. E., 2004 | North America | BDI-II |  | 49 | 68.9±10.9 | NA | NA | 100 | 37.6 |  | NA | NA | NA | NA |
| Reiff, J., 2011 | Europe | Clinical Exam |  | 110 | 63.1±8.1 | 8.9±5.3 | 20.2±9.8 | 61.8 | 26.3 |  | NA |  | NA | NA |
| Reijnders, J. S., 2010 | Europe | Clinical Exam |  | 224 | 67.6 | NA | NA | 60.3 | 17 |  | NA | NA | NA | NA |
| Richard, I. H., 2006 | North America | GDS>15 |  | 90 | 66.7 | NA | NA | 45 | 14 |  | NA | NA | NA | NA |
| Richard, I. H., 2007 | North America | Self-report diagnosis |  | 345 | 67.1 | NA | NA | 64 | 32 |  | NA | NA | NA | NA |
| Riedel, O., 2012 | Europe | MADRS>=14 or current antidepressant use |  | 1449 | 70.7±8.4 | 5.5±5.1 | NA | 60.5 | 33.1 |  | NA | NA | NA | NA |
| Rodriguez-Violante, M., 2011 | Latin America | NMSQ |  | 232 | 63.3±11.3 | 6.6±5.2 | NA | 56 | 66.7 |  | NA | NA | NA | NA |
| Rodriguez-Violante, M., 2012 | Latin America | BDI>=17 |  | 147 | 62.1±11.7 | 6.3±5 | 23.14 | 50.3 | 33.3 |  | NA | NA | NA | NA |
| Romenets, S. R., 2012 | North America | HADS, BDI |  | 70 | 66.7±9.3 | 3.8±2.8 | 26.7±13.1 | 64.3 | 30.9 |  | NA | NA | NA | NA |
| Santangelo, G., 2009 | Europe | Clinical Exam |  | 125 | 64 | 7.7 | 17.06 | 59.2 | 52 |  | NA | NA | NA | NA |
| Scalzo, P., 2009 | Latin America | BDI>=18 |  | 37 | 65±7.9 | 7.7±4.7 | 29.8±19 | 51.3 | 37.8 |  | NA | NA | NA | NA |
| Schneider, C. B., 2010 | Europe | Clinical Exam |  | 215 | 68.2±8.9 | 7.4±6.6 | NA | 55 | 20.2 |  | NA | NA | NA | NA |
| Schneider, J. S., 2010 | North America | GDS-15>5 |  | 413 | 61.6 | 0.66 | 15.8 | 64.2 | 13.8 |  | NA | NA | NA | NA |
| Schrag, A., 2002 | Europe | BDI>17 |  | 124 | 72±10.9 | 6±4.6 | 22.7±11.1 | 52.3 | 20 |  | NA | NA | NA | NA |
| Shearer, J., 2012 | Europe | GDS15>=5 |  | 162 | 72.2±10 | 1.96±1.87 | 24±11.6 | 57 | 32.5 |  | NA | NA | NA | NA |
| Shine, J. M., 2012 | Australia | BDI-II>=14 |  | 96 | 65.4 | NA | 24.6±12.8 | 58.3 | 29 |  | NA | NA | NA | NA |
| Shulman, L. M., 2001 | North America | BDI>=10 |  | 99 | 67.4±8 | 6.9±5.7 | 22±9 | 53 | 36 |  | NA | NA | NA | NA |
| Shulman, L. M., 2002 | North America | BDI>=10 |  | 101 | 68.06±9.43 | 7.76±5.51 | 23.2±10.01 | 48 | 44 |  | NA | NA | NA | NA |
| Siri, C., 2010 | Europe | SCL-90R>1 |  | 486 | 65.82±10.25 | 12.06±5.93 | 20.66±11.3 | 58.6 | 47 |  | NA | NA | NA | NA |
| Skeie, G. O., 2012 | Europe | Self-report Symptom |  | 212 | NA | NA | NA | NA | 13.7 |  | 175 | NA | NA | 5.1 |
| Solla, P., 2011 | Europe | Clinical Exam |  | 349 | 71.7±9.6 | 9.6±6.1 | 34.1±15.5 | 53 | 30.9 |  | NA | NA | NA | NA |
| Solla, P., 2012 | Europe | Clinical Exam |  | 156 | 69.3±8.5 | 6.3±4.4 | 30.2±15.3 | 58.3 | 25 |  | NA | NA | NA | NA |
| Spica, V., 2012 | Europe | NMSQ |  | 107 | 69.1±6.0 | 7.1±4.4 | 35.1±12.3 | 68.2 | 49.5 |  | NA | NA | NA | NA |
| Starkstein, S., 2011 | International | Clinical Exam |  | 259 | 66.8 | 7.2 | 25.5 | 58.3 | 59 |  | NA | NA | NA | NA |
| Stella, F., 2009 | Latin America | Clinical Exam |  | 50 | 68 | 10.1 | NA | 56 | 34 |  | NA | NA | NA | NA |
| Stern, Y., 1993 | North America | Clinical Exam |  | 250 | 68.4 | 7.99 | NA | NA | 48.2 |  | NA | NA | NA | NA |
| Strutt, A. M., 2012 | North America | BDI |  | 22 | 64.7±8.12 | 5.50±4.35 | 22.3±9.01 | 72.7 | 13 |  | NA | NA | NA | NA |
| Surdhar, I., 2012 | North America | GDS-15>=5 or medication |  | 33 | NA | NA | NA | NA | 18.2 |  | 6 | 70.69±2.4 | 16.7 | NA |
| Thompson, A. W., 2011 | North America | Clinical Exam |  | 214 | 72.5±9.6 | 5.2±2.35 | 17.3±10.3 | 57.9 | 21.5 |  | NA | NA | NA | NA |
| Tremblay, C., 2012 | North America | BDI-II>=9 |  | 25 | 63±6 | 14±3 | 29.92±9.05 | 64 | 52 |  | 13 | 61.94±4.81 | 30.8 | NA |
| Valko, P. O., 2010 | Europe | BDI>=12 |  | 88 | 67.5±9.7 | 9.8±5.4 | 21.5±10.1 | 69 | 42 |  | NA | NA | NA | NA |
| Vanderheyden, J. E., 2010 | Europe | Clinical Exam |  | 1086 | 71.4 | 5.19 | 34.8 | 54.5 | 15.6 |  | NA | NA | NA | NA |
| Veazey, C., 2009 | North America | PRIME-MD |  | 80 | 70.8±8.6 | NA | NA | 98.8 | 52.5 |  | NA | NA | NA | NA |
| Veiga, B. A., 2009 | Latin America | Clinical Exam |  | 50 | NA | NA | NA | 50 | 42 |  | 50 | NA | NA | 10 |
| Velez Feijo, A., 2008 | Latin America | BDI>=14 |  | 35 | 63.74 | 6.94±4.17 | NA | 51.4 | 40 |  | 65 | 63.62±9.06 | 46.2 | 9 |
| Verbaan, D., 2008 | Europe | BDI>=14 |  | 419 | 61.1±11.5 | 10.5±6.5 | NA | 64 | 21 |  | 150 | 60.9±9.9 | 55 | NA |
| Vibha, D., 2011 | Asia | N/A |  | 134 | 58.3 | 5.4 | 29.4 | 67 | 14.9 |  | NA | NA | NA | NA |
| Wang, G., 2010 | Asia | NMSQ |  | 117 | 64.81±9.42 | 5.27±4.03 | NA | 64.1 | 43.2 |  | NA | NA | NA | NA |
| Wei, Y. J., 2010 | North America | Self-report diagnosis |  | 571 | NA | NA | NA | 45 | 39.4 |  | NA | NA | NA | NA |
| Weintraub, D., 2004 | North America | Clinical Exam |  | 114 | 72.5±7.5 | 7.4±5.4 | 25.3±12.8 | 99.1 | 29.7 |  | NA | NA | NA | NA |
| Winter, Y., 2011 | Europe | BDI>9 |  | 70 | 65±8.5 | 7.8 | 16.49 | 58.6 | 55.7 |  | NA | NA | NA | NA |
| Wolz, M., 2009 | Europe | Medical Records |  | 274 | 66.2±8.4 | 9.2±6.6 | NA | 65.3 | 24.1 |  | 234 | 64.2±8.8 | 29.5 | 9.8 |
| Young, A., 2002 | Australia | BDI>16 |  | 18 | 69.5 | NA | NA | 55.6 | 44.4 |  | NA | NA | NA | NA |
| Yu, B., 2010 | Asia | NMSQ |  | 90 | 61.39±9.97 | NA | NA | 59 | 67.8 |  | 270 | 62.26±12.10 | 58 | 32.2 |
| Zahodne, L. B., 2012 | North America | BDI>15 |  | 186 | 65.9 | 8.2 | 28.6±10.8 | 66 | 17.6 |  | NA | NA | NA | NA |
| Zahodne, L. B., 2012 | North America | BDI>=15 |  | 181 | 66.2±9.7 | 8.66 | NA | 66.3 | 17 |  | NA | NA | NA | NA |
| Zahodne, L. B., 2012 | Europe | Clinical Exam |  | 95 | 66.24±9.89 | 6.53±4.96 | NA | 68.4 | 28.4 |  | NA | NA | NA | NA |
| Zampieri, M., 2011 | Latin America | GDS-15>=5 |  | 30 | 57.93±9.08 | 9.07±5.11 | NA | 56.7 | 46.7 |  | NA | NA | NA | NA |
| Zhang, J. L., 2009 | Asia | CESD>=16 |  | 306 | NA | NA | 31.24±15.82 | 62.1 | 47.4 |  | NA | NA | NA | NA |
| Zheng, J., 2009 | Asia | HAM-D>=10 |  | 131 | 62±9.5 | 5.5±3.2 | NA | 67.9 | 74.8 |  | NA | NA | NA | NA |
| Ziropadja, Lj, 2012 | Europe | HAM-D>=14 |  | 360 | 63.5 | 7.23 | 50.91 | 65 | 41.4 |  | NA | NA | NA | NA |
| van der Hoek, T. C., 2011 | Europe | BDI>=10 |  | 256 | 65.12±9.6 | 7.9±5.9 | NA | 60.1 | 49.2 |  | NA | NA | NA | NA |
| Total Number of Cases and Controls |  |  |  | 35662 |  |  |  |  |  |  | 6990 |  |  |  |
| **Studies with symptoms before PD diagnosis (N = 7)** | | | | | | | | | | | | | | |
| Chaudhuri, K. R., 2008 | International | Self-report Symptom |  | 54 | 66.7±10.04 | NA | NA | 57.7 | 44.4 |  | NA | NA | NA | NA |
| Fang, F., 2010 | North America | Self-report diagnosis |  | 992 | NA | NA | NA | 75 | 13.4 |  | 280000 | NA | 58.8 | 10.4 |
| Gaenslen, A., 2011 | Europe | Self-report Symptom |  | 93 | 67.9±7.3 | NA | NA | 64.5 | 23.7 |  | 93 | 67.7±7.2 | 64.5 | 13 |
| Henderson, R., 1992 | North America | Self-report Symptom |  | 164 | NA | NA | NA | 53.7 | 13.4 |  | 150 | NA | 35.3 | NA |
| Ishihara-Paul, L., 2008 | Europe | Structured Questionnaire |  | 175 | 63.9±8.8 | NA | NA | 52 | 23.4 |  | 20680 | 58.8±9.3 | 43.4 | 15.7 |
| Sanchez-Ferro, A., 2011 | Europe | Self-report symptom and medication |  | 23 | 75.7±6.3 | NA | NA | 43.5 | 30.4 |  | 92 | 75.3±7.5 | 41.3 | 23.9 |
| Shiba, M., 2000 | North America | Medical Records |  | 196 | 71 | NA | NA | 62 | 24 |  | 196 | 71 | 62 | 14.8 |
| Total Number of Cases and Controls |  |  |  | 1697 |  |  |  |  |  |  | 301000 |  |  |  |

| **Table S5. Study characteristics on EDS among PD patients and controls after and prior to disease diagnosis** | | | | | | | | | | | | | | |
| --- | --- | --- | --- | --- | --- | --- | --- | --- | --- | --- | --- | --- | --- | --- |
|  |  |  |  |  |  |  |  |  |  |  |  |  |  |  |
|  | | | | Symptom Assessment Among Cases | | | | | |  | Symptom Assessment Among Controls | | | |
| Authors, Year | Continent | Method |  | N | Mean Age at Assessment | Mean Duration (years) | Mean UPDRS-III | Men, % | Prevalence, % |  | N | Mean Age at Assessment | Men, % | Prevalence, % |
| **Studies with symptoms after PD diagnosis (N = 74)** | | | | | | | | | | | | | | |
| Adler, C. H., 2011 | North America | ESS>10 |  | 49 | 72.8±8.6 | NA | NA | 61 | 48 |  | 175 | 79.4±7.3 | 45 | 11 |
| Alves, G., 2005 | Europe | Self-report Symptom |  | 232 | 73.5±8.5 | 9.1±5.7 | 28.5±15.8 | NA | 17.7 |  | NA | NA | NA | NA |
| Amick, M. M., 2007 | North America | ESS>10 |  | 21 | 68.1 | 5.94 | 28.4 | 66.7 | 24 |  | NA | NA | NA | NA |
| Barone, P., 2009 | Europe | Self-report Symptom |  | 1072 | 67.4±9.4 | 5.1 | 24.2±13.1 | 60.4 | 21.2 |  | NA | NA | NA | NA |
| Boddy, F., 2007 | Europe | ESS>10 |  | 39 | 75±6 | 6.59 | 25±10 | 74.4 | 41 |  | 41 | 75±7 | 53.7 | 10 |
| Borek, L. L., 2006 | Europe | ESS>10 |  | 120 | 71±10.8 | 7.3±4.7 | NA | 62.5 | 39.8 |  | NA | NA | NA | NA |
| Braga-Neto, P., 2004 | Latin America | ESS>=10 |  | 86 | 65.8±10.4 | 6.0±4.6 | 18.5 | 64 | 33.7 |  | NA | NA | NA | NA |
| Brodsky, M. A., 2003 | North America | ESS>=10 |  | 101 | 65.4±11 | NA | NA | NA | 40.6 |  | 100 | 61.4±17 | NA | 19 |
| Bryant, M. S., 2012 | North America | ESS>=10 |  | 54 | 70.76 | 8.75 | 20.82 | 75.9 | 62.9 |  | NA | NA | NA | NA |
| Buskova, J., 2011 | Europe | ESS>=10 |  | 20 | 60.7±11 | 2.46 | 18.5±7 | 85 | 10 |  | 15 | 60.2±10 | 93.3 | NA |
| Chaudhuri, K. R., 2010 | Europe | NMSQ |  | 242 | 68.0±10.0 | 8.0±5.8 | NA | 63.2 | 34.7 |  | NA | NA | NA | NA |
| Cheon, S. M., 2008 | Asia | NMSQ |  | 74 | 64.9±8.6 | 6.4±6.1 | NA | 37.8 | 26.4 |  | NA | NA | NA | NA |
| Cochen De Cock, V., 2010 | Europe | ESS>10 |  | 50 | 62.1±9.8 | 6.8±4.0 | NA | 70 | 36 |  | 50 | 62.4±13.8 | 70 | 12 |
| Coelho, M., 2010 | Europe | Self-report Symptom |  | 50 | 74.1±7.0 | 17.94±6.3 | 49.18±13.1 | 46 | 36 |  | NA | NA | NA | NA |
| Compta, Y., 2009 | Europe | ESS>10 |  | 41 | 70.6 | 10.17 | 32.28 | 51.2 | 53.7 |  | 22 | 70.4±9 | 45 | NA |
| De Cock, V. C., 2007 | Europe | ESS>10 |  | 100 | 63.6 | 7.3 | 20 | 65.9 | 36.1 |  | NA | NA | NA | NA |
| Diederich, N. J., 2005 | Europe | ESS>10 or self-report symptom |  | 46 | 64.26±9.4 | 7.01±4.60 | NA | 78.3 | 41 |  | NA | NA | NA | NA |
| Erro, R., 2012 | Europe | NMSQ |  | 66 | 58.2±8.6 | 0.5 | 15.1±6.9 | 60.6 | NA |  | NA | NA | NA | NA |
| Erro, R., 2012 | Europe | NMSQ |  | 97 | 57.6±8.5 | 1.14±0.48 | 16.2±7.4 | 60.8 | 3.3 |  | NA | NA | NA | NA |
| Fabbrini, G., 2002 | Europe | ESS>=10 |  | 75 | 64.7 | 6 | 21.8 | 60 | 53 |  | 25 | 64.5±7.8 | 60 | 4 |
| Ferreira, J. J., 2006 | Europe | ESS>=10 |  | 176 | 65±10 | 10±5.9 | 19.8±11.1 | 61.4 | 33.5 |  | 174 | 64±10 | 46.6 | 16.1 |
| Frauscher, B., 2004 | Europe | ESS>10 |  | 46 | 68.4±9.9 | 12.2±5.2 | NA | 58.7 | 32.6 |  | NA | NA | NA | NA |
| Furumoto, H., 2004 | Asia | ESS>=10 |  | 53 | 70.6±9.2 | 6.2±5.0 | NA | 39.6 | 11.3 |  | 17 | 74.1±8.4 | 41.2 | NA |
| Gerbin, M., 2012 | North America | ESS>10 |  | 40 | 68.9±8.0 | 9.3±5.6 | NA | 65 | 25 |  | 120 | 70.3±6.0 | 36.7 | 9.2 |
| Ghorayeb, I., 2007 | Europe | ESS>=10 |  | 1625 | 69.5±9.3 | 6.1±4.6 | NA | 57 | 29 |  | NA | NA | NA | NA |
| Goetz, C. G., 2010 | North America | PSQI |  | 89 | 67.7±9.5 | 10.3±6.9 | 28.35 | 54 | 36 |  | NA | NA | NA | NA |
| Goulart, F. O., 2009 | Latin America | ESS>=10 |  | 50 | 70.5 | 6.3 | 31.5 | 44 | 44 |  | 50 | 73.9 | 14 | 16 |
| Hagell, P., 2007 | Europe | Self-report Symptom |  | 118 | 63.9±9.6 | 8.4±5.7 | 17 | 54 | 25.4 |  | NA | NA | NA | NA |
| Havlikova, E., 2008 | Europe | ESS>10 |  | 78 | 68.8±8.7 | 7.2±6.8 | NA | 52.6 | 25.6 |  | NA | NA | NA | NA |
| Henderson, J. M., 2003 | Australia | Self-report Symptom |  | 38 | 72±1 | 8.2±1 | NA | 45 | 45 |  | 32 | 74±2 | 34.3 | 6 |
| Hobson, D. E., 2002 | North America | ESS>7 |  | 638 | 65.7±10.6 | 8.1±5.4 | NA | NA | 51 |  | NA | NA | NA | NA |
| Hogl, B., 2003 | Australia | ESS>=10 |  | 99 | 67.7±10.3 | 7.4±6.7 | NA | 58.6 | 33 |  | 44 | 65.6±10.0 | 54.5 | 11.4 |
| Hu, M., 2011 | Europe | PD Sleep Scale |  | 197 | 71.8 | 9.1 | NA | 65.8 | 59.4 |  | NA | NA | NA | NA |
| Kagi, G., 2010 | Europe | NMSQ |  | 27 | 68.2±5.9 | 13.3±3 | NA | 59.3 | 48.1 |  | NA | NA | NA | NA |
| Kashihara, K., 2008 | Asia | Self-report Symptom |  | 194 | 70.3±9.4 | 5.0±4.6 | NA | 42 | 36.1 |  | 43 | 69.0±10.3 | 69.8 | 4.7 |
| Khedr, E. M., 2012 | Africa | NMSQ |  | 112 | 60.96±12.1 | 6.2±5.9 | NA | 68.8 | 39.3 |  | NA | NA | NA | NA |
| Kim, H. J., 2009 | Asia | NMSS |  | 23 | 64.3±8.5 | 0.8 | 16.5±9.0 | 47.8 | 8.7 |  | 23 | 65.3±9.1 | 47.8 | 4.3 |
| Kulisevsky, J., 2008 | Europe | ESS>10 |  | 1351 | 70.61±9.09 | 5.65±4.94 | NA | 55.6 | 26.4 |  | NA | NA | NA | NA |
| Kumar, S., 2003 | Asia | ESS>=8 |  | 149 | 58.37±10.45 | 5.7±3.85 | 31.2 | 84.6 | 21.5 |  | 115 | 56.5±11.45 | NA | 3 |
| Lavault, S., 2010 | Europe | ESS>10 |  | 61 | 64.3 | 6.89 | 18.9 | 64 | 36 |  | NA | NA | NA | NA |
| Marinus, J., 2003 | Europe | ESS>10 |  | 142 | 65.6±10.8 | 9.9±5.4 | NA | 60.5 | 26.7 |  | 100 | 61.4±11.2 | 48 | 3 |
| Martinez-Martin, P., 2007 | International | NMSQ |  | 525 | 67.66±10.46 | 6.96±5.3 | NA | 58 | 31.1 |  | NA | NA | NA | NA |
| Martinez-Martin, P., 2011 | International | NMSS |  | 411 | 64.48±9.92 | 8.07±5.75 | NA | 61.3 | 47.4 |  | NA | NA | NA | NA |
| Martinez-Martin, P., 2012 | International | NMSS |  | 950 | 64.43±9.90 | 7.99±5.78 | NA | 62.6 | 47 |  | NA | NA | NA | NA |
| Medeiros, C. A., 2007 | Latin America | ESS>10 |  | 20 | 61.8±7.13 | 7.05±4.87 | NA | 88.9 | 40 |  | NA | NA | NA | NA |
| Monaca, C., 2006 | Europe | ESS>=10 |  | 222 | 65.5±9.6 | 9.5±6.5 | 15±9 | 43.2 | 43.2 |  | NA | NA | NA | NA |
| Moreno-Lopez, C., 2011 | International | ESS>10 |  | 86 | 62.9±7.9 | 11.7±7.8 | NA | 48.8 | 29 |  | 86 | 62.5±7.7 | 48.8 | 2 |
| Nazzaro, J. M., 2011 | North America | NMSQ |  | 24 | 64.2±6.5 | 10.6±3.5 | 23.5±6.1 | 67 | 29.2 |  | NA | NA | NA | NA |
| O'Suilleabhain, P. E., 2002 | North America | ESS>=10 |  | 368 | 66.8±10.3 | 7.9±6.0 | 23±15 | NA | 54.6 |  | 243 | NA | NA | NA |
| Oberholzer, M., 2011 | Europe | ESS>=10 |  | 417 | 69.4±9.1 | 10.5±7.3 | NA | 68 | 47 |  | NA | NA | NA | NA |
| Pacchetti, C., 2004 | Europe | ESS>10 |  | 289 | 68.3 | NA | 24.41±12.35 | 53.6 | 26.6 |  | NA | NA | NA | NA |
| Pacchetti, C., 2005 | Europe | ESS>10 |  | 289 | 68.3 | 8.37±5.35 | 21.63±11.99 | 53.6 | 24 |  | NA | NA | NA | NA |
| Perez Lloret, S., 2008 | Latin America | Self-report Symptom |  | 70 | 69±8 | 7.9±5.7 | NA | 49 | 55.7 |  | NA | NA | NA | NA |
| Poryazova, R., 2010 | Europe | ESS>10 |  | 30 | 65±10 | 8.2±6.6 | 25±10 | 80 | 57 |  | NA | NA | NA | NA |
| Raggi, A., 2011 | Europe | NMSQ |  | 86 | 63.9±11.6 | NA | NA | 64 | 17.7 |  | NA | NA | NA | NA |
| Razmy, A., 2004 | North America | ESS>=7 |  | 80 | 64.3±10.3 | 9.3±5.9 | NA | 65 | 43 |  | NA | NA | NA | NA |
| Rodriguez-Violante, M., 2011 | Latin America | NMSQ |  | 232 | 63.3±11.3 | 6.6±5.2 | NA | 56 | 28.6 |  | NA | NA | NA | NA |
| Romenets, S. R., 2012 | North America | ESS>10 or SCOPA-SLEEP DS>5 |  | 70 | 66.7±9.3 | 3.8±2.8 | 26.7±13.1 | 64.3 | 48.6 |  | NA | NA | NA | NA |
| Schlesinger, I., 2003 | North America | Structured Questionnaire |  | 70 | 63.2 | 8.91 | NA | 65.7 | 34.3 |  | NA | NA | NA | NA |
| Shearer, J., 2012 | Europe | N/A |  | 162 | 72.2±10 | 1.96±1.87 | 24±11.6 | 57 | 17.3 |  | NA | NA | NA | NA |
| Shpirer, I., 2006 | Asia | ESS>10 |  | 46 | 67.3±9.3 | 8.3±5.8 | NA | 50 | 50 |  | 30 | 65.2±5.4 | 60 | NA |
| Solla, P., 2012 | Europe | NMSS |  | 156 | 69.3±8.5 | 6.3±4.4 | 30.2±15.3 | 58.3 | 53.2 |  | NA | NA | NA | NA |
| Spica, V., 2012 | Europe | NMSQ |  | 107 | 69.1±6.0 | 7.1±4.4 | 35.1±12.3 | 68.2 | 20.6 |  | NA | NA | NA | NA |
| Stevens, S., 2004 | North America | MSLT<5 |  | 19 | 60±8.7 | 7.4±5.4 | 24.6±9.7 | 73.7 | 47 |  | NA | NA | NA | NA |
| Suzuki, K., 2008 | Asia | ESS>10 |  | 188 | 66.4±8.7 | 6.9±5.3 | 32.9±18.1 | 45.2 | 21.3 |  | 144 | 65.1±6.8 | 44.4 | NA |
| Svensson, E., 2012 | Europe | Self-report Symptom |  | 176 | 68.5±8.8 | 7.3±5.3 | 22.3±11.7 | 59 | 28 |  | NA | NA | NA | NA |
| Tan, E. K., 2002 | Asia | ESS>=10 |  | 201 | 63.2±10 | 5.5 | NA | 62.6 | 19.9 |  | 214 | 61.4±6.8 | 61.2 | 9.8 |
| Tandberg, E., 1999 | Europe | Self-report Symptom |  | 239 | 73.8±8.4 | 9.1±5.8 | 28.28 | NA | 26.8 |  | 100 | 72.8±8.2 | NA | 10 |
| Valko, P. O., 2010 | Europe | ESS>=10 |  | 88 | 67.5±9.7 | 9.8±5.4 | 21.5±10.1 | 69 | 48 |  | NA | NA | NA | NA |
| Verbaan, D., 2008 | Europe | SCOPA-SLEEP DS>=5 |  | 419 | 61.1±11.5 | 10.5±6.5 | NA | 64 | 43 |  | 150 | 60.9±9.9 | 55 | 10 |
| Vibha, D., 2011 | Asia | ESS |  | 134 | 58.3 | 5.4 | 29.4 | 67 | 11.2 |  | NA | NA | NA | NA |
| Wang, G., 2010 | Asia | NMSQ |  | 117 | 64.81±9.42 | 5.27±4.03 | NA | 64.1 | 22.5 |  | NA | NA | NA | NA |
| Yong, M. H., 2011 | Asia | ESS>=10 |  | 56 | 65.4±9.1 | 6.4±4.1 | 21.5±11.8 | 60.7 | 19.1 |  | 68 | 59.3±9.1 | 55.9 | 2.9 |
| Yu, B., 2010 | Asia | NMSQ |  | 90 | 61.39±9.97 | NA | NA | 59 | 25.5 |  | 270 | 62.26±12.10 | 58 | 32.2 |
| Total Number of Cases and Controls |  |  |  | 14378 |  |  |  |  |  |  | 2451 |  |  |  |
| **Studies with symptoms before PD diagnosis (N = 1)** | | | | | | | | | | | | | | |
| Abbott, R. D., 2005 | North America | Self-report Symptom |  | 43 | NA | NA | NA | 100 | 20.9 |  | 3035 | 77.1 | 100 | 8 |
| Total Number of Cases and Controls |  |  |  | 43 |  |  |  |  |  |  | 3035 |  |  |  |

| **Table S6. Study characteristics on hyposmia among PD patients and controls after and prior to disease diagnosis** | | | | | | | | | | | | | | |
| --- | --- | --- | --- | --- | --- | --- | --- | --- | --- | --- | --- | --- | --- | --- |
|  |  |  |  |  |  |  |  |  |  |  |  |  |  |  |
|  | | | | Symptom Assessment Among Cases | | | | | |  | Symptom Assessment Among Controls | | | |
| Authors, Year | Continent | Method |  | N | Mean Age at Assessment | Mean Duration (years) | Mean UPDRS-III | Men, % | Prevalence, % |  | N | Mean Age at Assessment | Men, % | Prevalence, % |
| **Studies with symptoms after PD diagnosis (N = 39)** | | | | | | | | | | | | | | |
| Ansari, K. A., 1975 | North America | Smell test |  | 22 | 58 | 7 | NA | 100 | 45.5 |  | NA | NA | NA | NA |
| Baba, T., 2011 | Asia | OSIT-J<=7 |  | 69 | 64.6 | 5.4 | 17.6 | NA | 75.4 |  | NA | NA | NA | NA |
| Barone, P., 2009 | Europe | Self-report Symptom |  | 1072 | 67.4±9.4 | 5.1 | 24.2±13.1 | 60.4 | 26.9 |  | NA | NA | NA | NA |
| Berendse, H. W., 2011 | Europe | UPSIT |  | 96 | 64.9±9.7 | 4.8±5.3 | NA | 61.5 | 94 |  | 0 | NA | NA | NA |
| Boesveldt, S., 2008 | Europe | SS-TDI-I<95% prediction interval |  | 400 | 59.2 | NA | NA | 62.5 | 65 |  | 150 | NA | 58 | NA |
| Brodoehl, S., 2012 | Europe | SS-TDI<=27 |  | 16 | 66±8.2 | 6.8±3.86 | 16±8.6 | 50 | 100 |  | 16 | 66±5.6 | 50 | NA |
| Busse, K., 2012 | Europe | SS-12<8 |  | 453 | 64.8 | 8.63 | 30.9 | 67.5 | 71.9 |  | NA | NA | NA | NA |
| Chen, W., 2012 | Asia | SS-16<95% confidence interval |  | 110 | 64.6±7.1 | 4.3±3.7 | 17.5±9.0 | 60 | 66.4 |  | 110 | 64.4±7.8 | 60 | NA |
| Damholdt, M. F., 2011 | Europe | B-SIT<9 |  | 63 | 69.4±6.3 | 7.24 | 25.4 | 66.7 | 90.5 |  | 28 | 68.35 | 46.4 | 15 |
| Double, K. L., 2003 | Australia | B-SIT |  | 49 | 68±8 | 5±1 | 16±1.3 | 53 | 82 |  | 52 | 71±10 | 41.5 | 23 |
| Goldstein, D. S., 2010 | North America | UPSIT |  | 23 | 63.1 | NA | NA | 72.2 | 97 |  | 0 | NA | NA | NA |
| Haehner, A., 2009 | International | SS-TDI<30.5 |  | 400 | 64.3 | 6.6 | 22.7 | 65.8 | 74.5 |  | NA | NA | NA | NA |
| Henderson, J. M., 2003 | Australia | Self-report Symptom |  | 38 | 72±1 | 8.2±1 | NA | 45 | 68 |  | 32 | 74±2 | 34.3 | 3 |
| Herting, B., 2008 | Europe | SS-TDI |  | 27 | 49 | 7 | 24.5 | 81 | 100 |  | NA | NA | NA | NA |
| Hummel, T., 2005 | Europe | SS-TDI |  | 11 | 57.3 | 14.7 | 13.4 | 55 | 100 |  | NA | NA | NA | NA |
| Ibarretxe-Bilbao, N., 2010 | Europe | UPSIT<=33 |  | 24 | 56.1 | 3.05 | 14.7 | 69.6 | 91.7 |  | 24 | 57.3±11.3 | NA | 62.5 |
| Iijima, M., 2008 | Asia | Self-report Symptom |  | 54 | 69.7±8.1 | 4.7 | 14.5 | 61.1 | 51.9 |  | 50 | 69.3±7.3 | 60 | NA |
| Iijima, M., 2010 | Asia | OSIT-J<2 SD below mean |  | 40 | 66.6±10.6 | 4.83±4.32 | 17.2 | 62.5 | 50 |  | 40 | 67.8±7.0 | NA | NA |
| Izawa, M. O., 2012 | Asia | OSIT-J<=8 |  | 33 | 64.8±8.4 | 4.6±3.8 | 14.7±10.5 | 51.5 | 85 |  | 32 | 57.3±13.9 | 53 | 22 |
| Kim, J. Y., 2007 | Asia | B-SIT<=10 |  | 59 | 63.4±12.1 | NA | 7.2 | 63 | 86.4 |  | 25 | 59.9±11.6 | 40 | 44 |
| Meusel, T., 2010 | Europe | SS-TDI<30 |  | 19 | 65 | 11 | NA | 74 | 94.7 |  | 0 | NA | NA | NA |
| Millar Vernetti, P., 2012 | Latin America | SS-TDI<28.4 |  | 72 | 65.3±1.3 | 4.9 | NA | 56 | 82 |  | 25 | 60.9±2.4 | 36 | 16 |
| Morley, J. F., 2011 | North America | UPSIT<25th percentile |  | 248 | 64±10 | 6.6±5.4 | 22±10 | 75 | 85 |  | NA | NA | NA | NA |
| Muller, A., 2002 | Europe | SS-TDI<=30 |  | 37 | NA | NA | NA | NA | 100 |  | NA | NA | NA | NA |
| Muller, B., 2011 | Europe | Smell test |  | 207 | 67.9 | 2.3±1.8 | 23.2 | 58.9 | 58.9 |  | 175 | 67.5 | 59.4 | 20.6 |
| Parma, V., 2012 | Europe | UPSIT |  | 12 | 67.75 | 2.33 | NA | 50 | 100 |  | 12 | 65.83 | NA | NA |
| Potagas, C., 1998 | Europe | Smell test |  | 80 | 66.1±9.4 | NA | NA | 63.8 | 53.7 |  | 40 | 64.6±9.6 | 27.5 | 12.5 |
| Ramjit, A. L., 2010 | North America | UPSIT |  | 58 | 69.27±6.85 | 10.96±8.66 | 30.76±10.57 | NA | 96 |  | 51 | 66.45±9.17 | NA | 49 |
| Ruiz-Martinez, J., 2011 | Europe | B-SIT |  | 146 | 70.3±9.8 | 7.4±5.6 | 13.4±7.5 | 55.5 | 75 |  | NA | NA | NA | NA |
| Saunders-Pullman, R., 2011 | North America | UPSIT<15th percentile |  | 30 | 63.4 | 9.3 | 13.1 | 56.7 | 83.3 |  | 46 | 57.9 | 48.8 | 6.5 |
| Shah, M., 2009 | Europe | UPSIT |  | 75 | NA | NA | NA | NA | 65.3 |  | 74 | NA | NA | 4.1 |
| Sharma, J. C., 2012 | Europe | UPSIT |  | 99 | 69.2 | 5.57 | 17.8 | 60.7 | 100 |  | NA | NA | NA | NA |
| Shin, H. Y., 2012 | Asia | B-SIT<=10 |  | 30 | 62.3 | 2.83 | 8.4 | 70 | 86.6 |  | 30 | 61.6 | 56.7 | 53.3 |
| Suzuki, M., 2011 | Asia | OSIT-J<=7 |  | 94 | 68.6 | 5.1 | 23.1 | 50 | 80.9 |  | 29 | 66.1±8.8 | 31 | NA |
| Tissingh, G., 2001 | Europe | B-SIT<2 SD below mean |  | 41 | 56.4 | 4.8 | 20.8 | 61 | 71 |  | 18 | 55.3 | 44.4 | NA |
| Verbaan, D., 2008 | Europe | Sniffin' stick -16 |  | 293 | 60.2±10.6 | 11.8±6.3 | NA | 65 | 61 |  | 150 | NA | NA | 7 |
| Ward, C. D., 1983 | Europe | Smell test |  | 72 | 60 | 10 | NA | 62.5 | 39 |  | 53 | 58 | 34 | NA |
| Welge-Lussen, A., 2009 | Europe | SS-TDI<=30 |  | 18 | 60.4 | 4.9 | 28 | 55.6 | 100 |  | NA | NA | NA | NA |
| Wu, X., 2011 | Asia | FOODA>1 |  | 26 | 58.9 | 6.3±6.2 | NA | 53.8 | 46.2 |  | 26 | 58.9 ±9.5 | 53.8 | NA |
| Total Number of Cases and Controls |  |  |  | 4716 |  |  |  |  |  |  | 1288 |  |  |  |
| **Studies with symptoms before PD diagnosis (N = 6)** | | | | | | | | | | | | | | |
| Berg, D., 2012 | Europe | SS-12<9 |  | 10 | 74 | NA | 2 | 70 | 70 |  | 1342 | 62 | 57 | 26.3 |
| Chaudhuri, K. R., 2008 | International | Self-report Symptom |  | 54 | 66.7±10.04 | NA | NA | 57.7 | 37.2 |  | NA | NA | NA | NA |
| Gaenslen, A., 2011 | Europe | Self-report Symptom |  | 93 | 67.9±7.3 | NA | NA | 64.5 | 38.7 |  | 93 | 67.7±7.2 | 64.5 | 14 |
| Gonera, E. G., 1997 | Europe | Medical Records |  | 60 | NA | NA | NA | 51.7 | 3.3 |  | 58 | NA | 50 | 1.7 |
| Muller, A., 2002 | Europe | Self-report Symptom |  | 37 | NA | NA | NA | NA | 24 |  | NA | NA | NA | NA |
| Ross, G. W., 2008 | North America | B-SIT<6 |  | 19 | NA | NA | NA | 100 | 52.6 |  | 2248 | 79.7±4.1 | 100 | 24 |
| Total Number of Cases and Controls |  |  |  | 273 |  |  |  |  |  |  | 3741 |  |  |  |

| **Table S7. Study characteristics on RBD among PD patients and controls after and prior to disease diagnosis** | | | | | | | | | | | | | | |
| --- | --- | --- | --- | --- | --- | --- | --- | --- | --- | --- | --- | --- | --- | --- |
|  |  |  |  |  |  |  |  |  |  |  |  |  |  |  |
|  | | | | Symptom Assessment Among Cases | | | | | |  | Symptom Assessment Among Controls | | | |
| Authors, Year | Continent | Method |  | N | Mean Age at Assessment | Mean Duration (years) | Mean UPDRS-III | Men, % | Prevalence, % |  | N | Mean Age at Assessment | Men, % | Prevalence, % |
| **Studies with symptoms after PD diagnosis (N = 71)** | | | | | | | | | | | | | | |
| Adler, C. H., 2011 | North America | MSQ |  | 49 | 72.8±8.6 | NA | NA | 61 | 69 |  | 175 | 79.4±7.3 | 45 | 13 |
| Arnulf, I., 2005 | Europe | PSG |  | 15 | 67±6 | 12±9 | NA | 46.7 | 20 |  | 15 | 67±10 | 46.7 | NA |
| Barone, P., 2009 | Europe | Self-report Symptom |  | 1072 | 67.4±9.4 | 5.1 | 24.2±13.1 | 60.4 | 29.6 |  | NA | NA | NA | NA |
| Bjornara, K. A., 2012 | Europe | RBDSQ>=6 |  | 107 | 68.2 | 5.4 | 25.5 | 60.7 | 38 |  | NA | NA | NA | NA |
| Bliwise, D. L., 2010 | North America | Clinical Exam |  | 55 | 63.4±10.7 | 9.6±6.8 | NA | 80 | 32.7 |  | NA | NA | NA | NA |
| Boeve, B. F., 2001 | North America | Self-report Symptom |  | 27 | NA | NA | NA | NA | 44.4 |  | NA | NA | NA | NA |
| Boeve, B. F., 2001 | North America | Self-report Symptom |  | 106 | NA | NA | NA | NA | 47 |  | NA | NA | NA | NA |
| Borek, L. L., 2007 | North America | Clinical Exam |  | 120 | 71.06 | 7.29 | NA | 62.5 | 30 |  | NA | NA | NA | NA |
| Bugalho, P., 2011 | Europe | RBDSQ>4 and self-report symptom |  | 75 | 72.6 | 2.8±1.36 | 18.1±11.56 | 42.7 | 55 |  | NA | NA | NA | NA |
| Bugalho, P., 2011 | Europe | RBDSQ>4 |  | 23 | 71.6±7.85 | 2.7±0.49 | NA | 100 | 39.1 |  | 20 | 70.9±7.70 | 100 | NA |
| Chaudhuri, K. R., 2010 | Europe | NMSQ |  | 242 | 68.0±10.0 | 8.0±5.8 | NA | 63.2 | 38.7 |  | NA | NA | NA | NA |
| Cheon, S. M., 2008 | Asia | NMSQ |  | 74 | 64.9±8.6 | 6.4±6.1 | NA | 37.8 | 34.7 |  | NA | NA | NA | NA |
| Chou, K. L., 2007 | North America | Clinical Exam |  | 31 | 67.64 | 6.97 | NA | 100 | 38.7 |  | NA | NA | NA | NA |
| Comella, C. L., 1998 | North America | Clinical Exam |  | 61 | 67.9±10.3 | 9.5±7 | NA | 60.7 | 14.8 |  | NA | NA | NA | NA |
| De Cock, V. C., 2007 | Europe | PSG |  | 100 | 63.6 | 7.3 | 20 | 65.9 | 41 |  | NA | NA | NA | NA |
| Diederich, N. J., 2005 | Europe | PSG |  | 46 | 64.26±9.4 | 7.01±4.60 | NA | 78.3 | 30 |  | NA | NA | NA | NA |
| Erro, R., 2012 | Europe | NMSQ |  | 66 | 58.2±8.6 | 0.5 | 15.1±6.9 | 60.6 | 37.9 |  | NA | NA | NA | NA |
| Erro, R., 2012 | Europe | NMSQ |  | 97 | 57.6±8.5 | 1.14±0.48 | 16.2±7.4 | 60.8 | 32.9 |  | NA | NA | NA | NA |
| Ferri, R., 2012 | Europe | PSG |  | 27 | 67.9±7.45 | 4.3±3.89 | NA | 63 | 59.3 |  | 19 | 67.5±7.28 | 37.8 | NA |
| Gagnon, J. F., 2002 | North America | PSG |  | 33 | 62.9±11.8 | 5.9±4.4 | NA | 63.6 | 33 |  | 16 | 62.3±6.87 | 62.5 | NA |
| Gagnon, J. F., 2004 | North America | PSG |  | 15 | 64.5±8.1 | 5.5±4.4 | NA | 60 | 46.7 |  | 15 | 65.5±6.3 | 60 | NA |
| Garcia-Borreguero, D., 2002 | Europe | Clinical Exam |  | 15 | 73.8±4.9 | 2.8±1.3 | NA | 60 | NA |  | 14 | 69.3±4.9 | NA | NA |
| Gjerstad, M. D., 2006 | Europe | SSQ |  | 232 | 73.5 | 9.1 | 28.47 | 49.1 | 14.7 |  | NA | NA | NA | NA |
| Goetz, C. G., 2010 | North America | PSQI |  | 89 | 67.7±9.5 | 10.3±6.9 | 28.35 | 54 | 12 |  | NA | NA | NA | NA |
| Kagi, G., 2010 | Europe | NMSQ |  | 27 | 68.2±5.9 | 13.3±3 | NA | 59.3 | 48.1 |  | NA | NA | NA | NA |
| Khedr, E. M., 2012 | Africa | NMSQ |  | 112 | 60.96±12.1 | 6.2±5.9 | NA | 68.8 | 15.2 |  | NA | NA | NA | NA |
| Kotagal, V., 2012 | North America | MSQ |  | 80 | 64.7 | 6±3.9 | 25.94 | 72.5 | 33.8 |  | NA | NA | NA | NA |
| Lavault, S., 2010 | Europe | Structured Questionnaire |  | 61 | 64.3 | 6.89 | 18.9 | 64 | 64 |  | NA | NA | NA | NA |
| Lee, A. H., 2012 | North America | PDSS |  | 191 | 63.1±10 | 6.4±5.4 | 21.64 | 71.7 | 50 |  | NA | NA | NA | NA |
| Lee, J. E., 2010 | Europe | Clinical Exam |  | 447 | 63.8±9.2 | 6.13 | 18.97 | 46.3 | 36.7 |  | NA | NA | NA | NA |
| Marion, M. H., 2008 | Europe | MSQ |  | 65 | 68.06±11.18 | 7.44±6.64 | NA | 63.1 | 36.9 |  | NA | NA | NA | NA |
| Martinez-Martin, P., 2007 | International | NMSQ |  | 525 | 67.66±10.46 | 6.96±5.3 | NA | 58 | 35.7 |  | NA | NA | NA | NA |
| Matuja, W. B., 2008 | Africa | Self-report Symptom |  | 42 | 61.5 | NA | NA | 59.5 | 28.6 |  | NA | NA | NA | NA |
| Meral, H., 2007 | Asia | Clinical Exam |  | 79 | 67.1 | 6.36 | 20.14 | 63.3 | 46 |  | NA | NA | NA | NA |
| Morgante, L., 2011 | Europe | Clinical Exam |  | 442 | 66.1±9.0 | 3.3 | 18.1±9.1 | 62.5 | 23.5 |  | NA | NA | NA | NA |
| Morgante, L., 2012 | Europe | Clinical Exam |  | 480 | 66.27 | 3.37 | 17.87 | 62.7 | 24.2 |  | NA | NA | NA | NA |
| Munhoz, R. P., 2010 | Latin America | Clinical Exam |  | 1142 | 67.1±10.9 | 7.7±5.3 | NA | 55.2 | 49.9 |  | NA | NA | NA | NA |
| Naismith, S. L., 2010 | Australia | RBDSQ>=5 |  | 22 | 63.4±7.5 | NA | NA | 50 | 59.1 |  | NA | NA | NA | NA |
| Nihei, Y., 2012 | Asia | RBDSQ-J>=5 |  | 469 | 71±8.3 | 6.8±5.5 | 10.9±4.4 | 46.7 | 31.1 |  | NA | NA | NA | NA |
| Nomura, T., 2011 | Asia | Clinical Exam |  | 45 | 72.9±9.1 | 8.6±7.2 | NA | 48.9 | 42 |  | NA | NA | NA | NA |
| Nomura, T., 2011 | Asia | PSG |  | 49 | 70.3±11.2 | NA | NA | 42.9 | 34.7 |  | NA | NA | NA | NA |
| Nomura, T., 2012 | Asia | PSG |  | 93 | 73.4±7.9 | 7.3±6.9 | NA | 41.9 | 32.3 |  | NA | NA | NA | NA |
| O'Sullivan, S. S., 2011 | Europe | Clinical Interview |  | 55 | 64.4±9.9 | 10.5±6.7 | NA | 76.1 | 34.5 |  | 48 | 57.9±10.6 | 81.3 | NA |
| Onofrj, M., 2002 | Europe | PSG |  | 80 | NA | NA | NA | NA | 6.3 |  | NA | NA | NA | NA |
| Onofrj, M., 2011 | Europe | N/A |  | 98 | 69.8±5.1 | NA | 18.2 | 60 | 24 |  | NA | NA | NA | NA |
| Oudiette, D., 2009 | Europe | PSG |  | 100 | NA | NA | NA | NA | 60 |  | NA | NA | NA | NA |
| Pacchetti, C., 2004 | Europe | QSMDPD |  | 289 | 68.3 | NA | 24.41±12.35 | 53.6 | 66.1 |  | NA | NA | NA | NA |
| Pacchetti, C., 2005 | Europe | Clinical Exam |  | 289 | 68.3 | 8.37±5.35 | 21.63±11.99 | 53.6 | 26.6 |  | NA | NA | NA | NA |
| Perez Lloret, S., 2008 | Latin America | NMSQ |  | 70 | 69±8 | 7.9±5.7 | NA | 49 | 30 |  | NA | NA | NA | NA |
| Postuma, R. B., 2008 | North America | PSG |  | 36 | 67 | 6.37 | 21.53 | 69.4 | 58.3 |  | NA | NA | NA | NA |
| Postuma, R. B., 2011 | North America | PSG |  | 53 | 65.9±9.5 | 5.4±3.6 | 19.1±10.4 | 72 | 60.3 |  | 31 | 67.3±8.0 | 74 | NA |
| Raggi, A., 2011 | Europe | NMSQ |  | 86 | 63.9±11.6 | NA | NA | 64 | 43.8 |  | NA | NA | NA | NA |
| Ratti, P. L., 2012 | Europe | PSG |  | 41 | 63.6±9.5 | 6.1±6.0 | 17.9±9.7 | 61 | 36.6 |  | NA | NA | NA | NA |
| Rodriguez-Violante, M., 2011 | Latin America | NMSQ |  | 232 | 63.3±11.3 | 6.6±5.2 | NA | 56 | 33.2 |  | NA | NA | NA | NA |
| Romenets, S. R., 2012 | North America | PSG |  | 98 | 66.3 | 5.75 | 23.78 | 66.3 | 55.1 |  | NA | NA | NA | NA |
| Romenets, S. R., 2012 | North America | Clinical Exam |  | 70 | 66.7±9.3 | 3.8±2.8 | 26.7±13.1 | 64.3 | 48.5 |  | NA | NA | NA | NA |
| Ruiz-Martinez, J., 2011 | Europe | Medical Records |  | 146 | 70.3±9.8 | 7.4±5.6 | 13.4±7.5 | 55.5 | 42.5 |  | NA | NA | NA | NA |
| Scaglione, C., 2005 | Europe | ICSD-R |  | 195 | 64.5±8.7 | 8.1±5.1 | 27.6 | 58.5 | 32.8 |  | NA | NA | NA | NA |
| Sinforiani, E., 2006 | Europe | Clinical Exam |  | 110 | 66.6 | 10.04 | 25.61 | 59.1 | 71.8 |  | NA | NA | NA | NA |
| Sixel-Doring, F., 2009 | Europe | PSG |  | 20 | 69±5 | 8±5 | NA | 65 | 65 |  | NA | NA | NA | NA |
| Spica, V., 2012 | Europe | NMSQ |  | 107 | 69.1±6.0 | 7.1±4.4 | 35.1±12.3 | 68.2 | 21.5 |  | NA | NA | NA | NA |
| Valko, P. O., 2010 | Europe | Clinical Exam |  | 88 | 67.5±9.7 | 9.8±5.4 | 21.5±10.1 | 69 | 27 |  | NA | NA | NA | NA |
| Vibha, D., 2010 | Asia | IAQ |  | 104 | 57.43±14.94 | 5.52±4.08 | NA | 75 | 25 |  | NA | NA | NA | NA |
| Vibha, D., 2011 | Asia | IAQ |  | 134 | 58.3 | 5.4 | 29.4 | 67 | 19.4 |  | NA | NA | NA | NA |
| Wallace, D. M., 2012 | North America | RBDSQ>=5 |  | 50 | 68±12 | 9±4 | NA | 68 | 44 |  | NA | NA | NA | NA |
| Wang, G., 2010 | Asia | Clinical Exam |  | 128 | 64.82±8.74 | 5.87±4.57 | 22.75±5.98 | 58.6 | 27.3 |  | NA | NA | NA | NA |
| Wetter, T. C., 2000 | Europe | PSG |  | 10 | 65.2±5.6 | 5.5±4.2 | 20.4±6.3 | 70 | 30 |  | 10 | 64.1±5.9 | 50 | NA |
| Wetter, T. C., 2001 | International | PSG |  | 45 | 61.58 | 5.66 | NA | 62.2 | 16 |  | NA | NA | NA | NA |
| Yong, M. H., 2011 | Asia | Clinical Exam |  | 56 | 65.4±9.1 | 6.4±4.1 | 21.5±11.8 | 60.7 | 22.6 |  | 68 | 59.3±9.1 | 55.9 | NA |
| Yoritaka, A., 2009 | Asia | Clinical Exam |  | 150 | 68.5±9.8 | 6.4±4.6 | NA | 46.7 | 54 |  | NA | NA | NA | NA |
| Yu, B., 2010 | Asia | NMSQ |  | 90 | 61.39±9.97 | NA | NA | 59 | 23.3 |  | 270 | 62.26±12.10 | 58 | 7.8 |
| Total Number of Cases and Controls |  |  |  | 10178 |  |  |  |  |  |  | 701 |  |  |  |
| **Studies with symptoms before PD diagnosis (N = 8)** | | | | | | | | | | | | | | |
| Boot, B. P., 2012 | North America | MSQ |  | 5 | NA | NA | NA | NA | 20 |  | 646 | 74.82 | 69.9 | 6.7 |
| Bugalho, P., 2011 | Europe | RBDSQ>4 |  | 75 | 72.6 | NA | 18.1±11.56 | 42.7 | 29.3 |  | NA | NA | NA | NA |
| Chaudhuri, K. R., 2008 | International | Self-report Symptom |  | 54 | 66.7±10.04 | NA | NA | 57.7 | 16.7 |  | NA | NA | NA | NA |
| Marion, M. H., 2008 | Europe | MSQ |  | 65 | 68.06±11.18 | NA | NA | 63.1 | 6.2 |  | NA | NA | NA | NA |
| Nomura, T., 2011 | Asia | Self-report Symptom |  | 49 | 70.3±11.2 | NA | NA | 42.9 | 10.2 |  | NA | NA | NA | NA |
| Postuma, R. B., 2008 | North America | PSG |  | 36 | 67 | NA | 21.53 | 69.4 | 25 |  | NA | NA | NA | NA |
| Scaglione, C., 2005 | Europe | ICSD-R |  | 195 | 64.5±8.7 | NA | 27.6 | 58.5 | 9 |  | NA | NA | NA | NA |
| Yoritaka, A., 2009 | Asia | Clinical Exam |  | 150 | 68.5±9.8 | NA | NA | 46.7 | 26 |  | NA | NA | NA | NA |
| Total Number of Cases and Controls |  |  |  | 629 |  |  |  |  |  |  | 646 |  |  |  |

| **Table S8. Summary of symptom assessment in previous studies** | | | | | | |  |  |  |  |  |  |
| --- | --- | --- | --- | --- | --- | --- | --- | --- | --- | --- | --- | --- |
|  |  |  |  |  |  |  |  |  |  |  |  |  |
|  | Cases | | | | | | Controls | | | | | |
| Symptom Assessment | ANX | CST | DEP | EDS | HYP | RBD | ANX | CST | DEP | EDS | HYP | RBD |
| **After Diagnosis** | | | | | | | | | | | | |
| Clinical exam / diagnosis | 8 | 1 | 44 | 1 | 0 | 37 | 0 | 0 | 5 | 0 | 0 | 4 |
| Structured questionnaire | 25 | 7 | 92 | 44 | 0 | 14 | 2 | 2 | 12 | 17 | 0 | 1 |
| Smell test | 0 | 0 | 0 | 0 | 36 | 0 | 0 | 0 | 0 | 0 | 16 | 0 |
| Medical records | 0 | 2 | 3 | 0 | 0 | 1 | 0 | 1 | 2 | 0 | 0 | 0 |
| Self-report diagnosis | 0 | 0 | 2 | 0 | 0 | 0 | 0 | 0 | 0 | 0 | 0 | 0 |
| Self-report symptom | 21 | 39 | 25 | 28 | 3 | 18 | 3 | 11 | 5 | 5 | 1 | 1 |
| N/A | 0 | 1 | 4 | 1 | 0 | 1 | 0 | 0 | 1 | 0 | 0 | 0 |
| **Before Diagnosis** | | | | | | | | | | | | |
| Clinical exam | 0 | 0 | 0 | 0 | 0 | 3 | 0 | 0 | 0 | 0 | 0 | 0 |
| Structured questionnaire | 1 | 0 | 1 | 0 | 0 | 3 | 1 | 0 | 1 | 0 | 0 | 1 |
| Smell test | 0 | 0 | 0 | 0 | 2 | 0 | 0 | 0 | 0 | 0 | 2 | 0 |
| Medical records | 1 | 1 | 1 | 0 | 1 | 0 | 1 | 1 | 1 | 0 | 1 | 0 |
| Self-report diagnosis | 0 | 0 | 1 | 0 | 0 | 0 | 0 | 0 | 1 | 0 | 0 | 0 |
| Self-report symptom | 2 | 3 | 4 | 1 | 3 | 2 | 1 | 2 | 2 | 1 | 1 | 0 |

**Table S9.** Abbreviations of assessment methods

| **Abbreviation** | **Assessment Methods** |
| --- | --- |
| BAI | Beck Anxiety Inventory |
| BDI | Beck Depression Inventory |
| BM | Bowel movement |
| B-SIT | Brief Smell Identification Test |
| CESD | Center for Epidemiologic Studies Depression Scale |
| CIDI-SFMD | Composite International Diagnostic Interview - Short Form for Major Depression |
| DSM-IV | Diagnostic and Statistical Manual of Mental Disorders - Fourth Edition |
| ESS | Epworth Sleepiness Scale |
| FOODA | Five Odors Olfactory Detection Arrays |
| GDS | Geriatric Depression Scale |
| HADS | Hospital Anxiety and Depression Scale |
| HAMA | Hamilton Anxiety Scale |
| HAMD | Hamilton Rating Scale for Depression |
| HANDS | The Harvard Department of Psychiatry National Depression Screening Day Scale |
| IAQ | Insomnia Acceptance Questionnaire |
| ICSD-R | International Classification of Sleep Disorders Revised |
| MAACL DYS | Multiple Affect Adjective Check List - Dysphoria |
| MADRS | Montgomery-Asberg Depression Scale |
| MSLT | Multiple Sleep Latency Test |
| MSQ | Mayo Sleep Questionnaire |
| NMSQ | Nonmotor Symptoms Questionnaire (NMSQuest) |
| NMSS | Nonmotor Symptoms Scale |
| NPI | Neuropsychiatric Inventory |
| OSIT-J | Odor Stick Identification Test for the Japanese |
| PACA | Patients Anxiety's and Concerns Assessment |
| PDQ | Parkinson's Disease Questionnaire |
| PDSS | Parkinson's Disease Sleep Scale |
| PRIME-MD | Primary Care Evaluation of Mental Disorders |
| PSG | Polysomnography |
| PSQI | Pittsburgh Sleep Quality Index |
| QSMDPD | Questionnaire on Sleep and Mendal Disorders in Parkinson's Disease |
| RBDSQ | REM Sleep Behavior Disorder Screening Questionnaire |
| RBDSQ-J | REM Sleep Behavior Disorder Screening Questionnaire - Japanse version |
| SAI | State-Anxiety Inventory |
| SCL-90R | Symptom Checklist 90 Revised |
| SCOPA-AUT | Scales for Outcomes in Parkinson's disease - Autonomic |
| SCOPA-SLEEP DS | Scales for Outcomes in Parkinson's Disease-Sleep Scale |
| SDS | Zung Self-Rating Depression Scale |
| SRQ-D | Self-Rating Questionnaire for Depression |
| SS | Sniffin' Sticks test |
| SSQ | Sleep and Settle Questionnaire |
| SS-TDI | Sniffin' Sticks odor Threshold, odor Discrimination, and odor Identification test |
| UPDRS | Unified Parkinson's Disease Rating Scale |
| UPSIT | University of Pennsylvania Smell Identification Test |
| ZDS | Zagazig Depression Scale |

**References in Supplemental Tables**

Abbott RD, Petrovitch H, White LR, et al. Frequency of bowel movements and the future risk of Parkinson's disease. Neurology 2001;57:456-462.

Abbott RD, Ross GW, White LR, et al. Excessive daytime sleepiness and subsequent development of Parkinson disease. Neurology 2005;65:1442-1446.

Adler CH, Hentz JG, Shill HA, et al. Probable RBD is increased in Parkinson's disease but not in essential tremor or restless legs syndrome. Parkinsonism Relat Disord 2011;17:456-458.

Alves G, Wentzel-Larsen T, Aarsland D, Larsen JP. Progression of motor impairment and disability in Parkinson disease: a population-based study. Neurology 2005;65:1436-1441.

Amick MM, D'Abreu A, Moro-de-Casillas ML, Chou KL, Ott BR. Excessive daytime sleepiness and on-road driving performance in patients with Parkinson's disease. J Neurol Sci 2007;252:13-15.

Anderson KE. Nonrecognition of depression in Parkinson's disease. Curr Neurol Neurosci Rep 2002;2:293-295.

Andreadou E, Anagnostouli M, Vasdekis V, et al. The impact of comorbidity and other clinical and sociodemographic factors on health-related quality of life in Greek patients with Parkinson's disease. Aging Ment Health 2011;15:913-921.

Ansari KA, Johnson A. Olfactory function in patients with Parkinson's disease. Journal of chronic diseases 1975;28:493-497.

Araujo Lima AM, Cordeiro Hirata Fde C, Sales de Bruin G, Salani Mota RM, Bruin VM. The influence of playing a non-reward game on motor ability and executive function in Parkinson's disease. Behav Neurol 2012;25:119-125.

Arnulf I, Merino-Andreu M, Bloch F, et al. REM sleep behavior disorder and REM sleep without atonia in patients with progressive supranuclear palsy. Sleep 2005;28:349-354.

Arun MP, Bharath S, Pal PK, Singh G. Relationship of depression, disability, and quality of life in Parkinson's disease: a hospital-based case-control study. Neurol India 2011;59:185-189.

Assogna F, Palmer K, Pontieri FE, et al. Alexithymia is a non-motor symptom of Parkinson disease. The American journal of geriatric psychiatry : official journal of the American Association for Geriatric Psychiatry 2012;20:133-141.

Baba T, Takeda A, Kikuchi A, et al. Association of olfactory dysfunction and brain. Metabolism in Parkinson's disease. Movement disorders : official journal of the Movement Disorder Society 2011;26:621-628.

Barone P, Antonini A, Colosimo C, et al. The PRIAMO study: A multicenter assessment of nonmotor symptoms and their impact on quality of life in Parkinson's disease. Mov Disord 2009;24:1641-1649.

Bassotti G, Maggio D, Battaglia E, et al. Manometric investigation of anorectal function in early and late stage Parkinson's disease. J Neurol Neurosurg Psychiatry 2000;68:768-770.

Becker C, Brobert GP, Johansson S, Jick SS, Meier CR. Risk of incident depression in patients with Parkinson disease in the UK. Eur J Neurol 2011;18:448-453.

Benito-Leon J, Louis ED, Bermejo-Pareja F. Population-based case-control study of morale in Parkinson's disease. Eur J Neurol 2009;16:330-336.

Benkler M, Agmon-Levin N, Hassin-Baer S, et al. Immunology, autoimmunity, and autoantibodies in Parkinson's disease. Clin Rev Allergy Immunol 2012;42:164-171.

Berendse HW, Roos DS, Raijmakers P, Doty RL. Motor and non-motor correlates of olfactory dysfunction in Parkinson's disease. J Neurol Sci 2011.

Berg D, Godau J, Seppi K, et al. The PRIPS study: screening battery for subjects at risk for Parkinson's disease. European journal of neurology : the official journal of the European Federation of Neurological Societies 2012.

Bjornara KA, Dietrichs E, Toft M. REM sleep behavior disorder in Parkinson's disease - Is there a gender difference? Parkinsonism Relat Disord 2012.

Bliwise DL, Trotti LM, Greer SA, Juncos JJ, Rye DB. Phasic muscle activity in sleep and clinical features of Parkinson disease. Ann Neurol 2010;68:353-359.

Boddy F, Rowan EN, Lett D, O'Brien JT, McKeith IG, Burn DJ. Subjectively reported sleep quality and excessive daytime somnolence in Parkinson's disease with and without dementia, dementia with Lewy bodies and Alzheimer's disease. Int J Geriatr Psychiatry 2007;22:529-535.

Boesveldt S, Verbaan D, Knol DL, et al. A comparative study of odor identification and odor discrimination deficits in Parkinson's disease. Movement disorders : official journal of the Movement Disorder Society 2008;23:1984-1990.

Boeve BF, Silber MH, Ferman TJ, Lucas JA, Parisi JE. Association of REM sleep behavior disorder and neurodegenerative disease may reflect an underlying synucleinopathy. Mov Disord 2001;16:622-630.

Boller F, Marcie P, Starkstein S, Traykov L. Memory and depression in Parkinson's disease. Eur J Neurol 1998;5:291-295.

Bolluk B, Ozel-Kizil ET, Akbostanci MC, Atbasoglu EC. Social anxiety in patients with Parkinson's disease. J Neuropsychiatry Clin Neurosci 2010;22:390-394.

Boot BP, Boeve BF, Roberts RO, et al. Probable rapid eye movement sleep behavior disorder increases risk for mild cognitive impairment and Parkinson disease: a population-based study. Ann Neurol 2012;71:49-56.

Borek LL, Kohn R, Friedman JH. Mood and sleep in Parkinson's disease. The Journal of clinical psychiatry 2006;67:958-963.

Borek LL, Kohn R, Friedman JH. Phenomenology of dreams in Parkinson's disease. Mov Disord 2007;22:198-202.

Bouwmans AE, Weber WE. Neurologists' diagnostic accuracy of depression and cognitive problems in patients with parkinsonism. BMC neurology 2012;12:37.

Braga-Neto P, da Silva-Junior FP, Sueli Monte F, de Bruin PF, de Bruin VM. Snoring and excessive daytime sleepiness in Parkinson's disease. J Neurol Sci 2004;217:41-45.

Brockmann K, Srulijes K, Hauser AK, et al. GBA-associated PD presents with nonmotor characteristics. Neurology 2011;77:276-280.

Brodoehl S, Klingner C, Volk GF, Bitter T, Witte OW, Redecker C. Decreased olfactory bulb volume in idiopathic Parkinson's disease detected by 3.0-Tesla magnetic resonance imaging. Movement disorders : official journal of the Movement Disorder Society 2012;27:1019-1025.

Brodsky MA, Godbold J, Roth T, Olanow CW. Sleepiness in Parkinson's disease: a controlled study. Mov Disord 2003;18:668-672.

Brown RG, Landau S, Hindle JV, et al. Depression and anxiety related subtypes in Parkinson's disease. J Neurol Neurosurg Psychiatry 2011;82:803-809.

Bryant MS, Rintala DH, Hou JG, et al. The relation of falls to fatigue, depression and daytime sleepiness in Parkinson's disease. European neurology 2012;67:326-330.

Bugalho P, da Silva JA, Cargaleiro I, Serra M, Neto B. Psychiatric symptoms screening in the early stages of Parkinson's disease. J Neurol 2012;259:124-131.

Bugalho P, da Silva JA, Neto B. Clinical features associated with REM sleep behavior disorder symptoms in the early stages of Parkinson's disease. J Neurol 2011;258:50-55.

Bugalho P, Paiva T. Dream features in the early stages of Parkinson's disease. Journal of neural transmission (Vienna, Austria : 1996) 2011;118:1613-1619.

Buskova J, Klempir J, Majerova V, et al. Sleep disturbances in untreated Parkinson's disease. J Neurol 2011;258:2254-2259.

Busse K, Heilmann R, Kleinschmidt S, et al. Value of combined midbrain sonography, olfactory and motor function assessment in the differential diagnosis of early Parkinson's disease. Journal of neurology, neurosurgery, and psychiatry 2012;83:441-447.

Butterfield LC, Cimino CR, Oelke LE, Hauser RA, Sanchez-Ramos J. The independent influence of apathy and depression on cognitive functioning in Parkinson's disease. Neuropsychology 2010;24:721-730.

Byrne KG, Pfeiffer R, Quigley EM. Gastrointestinal dysfunction in Parkinson's disease. A report of clinical experience at a single center. J Clin Gastroenterol 1994;19:11-16.

Caap-Ahlgren M, Dehlin O. Insomnia and depressive symptoms in patients with Parkinson's disease. Relationship to health-related quality of life. An interview study of patients living at home. Arch Gerontol Geriatr 2001;32:23-33.

Ceravolo R, Frosini D, Poletti M, et al. Mild affective symptoms in de novo Parkinson's disease patients: relationship with dopaminergic dysfunction. Eur J Neurol 2012.

Chagas MH, Crippa JA, Loureiro SR, et al. Validity of the PHQ-2 for the screening of major depression in Parkinson's disease: two questions and one important answer. Aging Ment Health 2011;15:838-843.

Chagas MH, Tumas V, Loureiro SR, et al. Validity of a Brazilian version of the Zung self-rating depression scale for screening of depression in patients with Parkinson's disease. Parkinsonism & related disorders 2010;16:42-45.

Chaudhuri KR, Martinez-Martin P. Quantitation of non-motor symptoms in Parkinson's disease. Eur J Neurol 2008;15 Suppl 2:2-7.

Chaudhuri KR, Martinez-Martin P, Schapira AH, et al. International multicenter pilot study of the first comprehensive self-completed nonmotor symptoms questionnaire for Parkinson's disease: the NMSQuest study. Mov Disord 2006;21:916-923.

Chaudhuri KR, Prieto-Jurcynska C, Naidu Y, et al. The nondeclaration of nonmotor symptoms of Parkinson's disease to health care professionals: an international study using the nonmotor symptoms questionnaire. Mov Disord 2010;25:704-709.

Chen W, Chen S, Kang WY, et al. Application of odor identification test in Parkinson's disease in China: a matched case-control study. Journal of the neurological sciences 2012;316:47-50.

Chen YK, Lu JY, Chan DM, et al. Anxiety disorders in Chinese patients with Parkinson's disease. Int J Psychiatry Med 2010;40:97-107.

Cheon SM, Ha MS, Park MJ, Kim JW. Nonmotor symptoms of Parkinson's disease: prevalence and awareness of patients and families. Parkinsonism Relat Disord 2008;14:286-290.

Cheon SM, Park MJ, Kim WJ, Kim JW. Non-motor off symptoms in Parkinson's disease. J Korean Med Sci 2009;24:311-314.

Cho JW, Baik JS, Lee MS. Mesencephalic midline change on transcranial sonography in early Parkinson's disease patients with depression. J Neurol Sci 2011;310:50-52.

Chou KL, Moro-De-Casillas ML, Amick MM, Borek LL, Friedman JH. Testosterone not associated with violent dreams or REM sleep behavior disorder in men with Parkinson's. Mov Disord 2007;22:411-414.

Cimino CR, Siders CA, Zesiewicz TA. Depressive symptoms in Parkinson disease: degree of association and rate of agreement of clinician-based and self-report measures. J Geriatr Psychiatry Neurol 2011;24:199-205.

Cochen De Cock V, Abouda M, Leu S, et al. Is obstructive sleep apnea a problem in Parkinson's disease? Sleep Med 2010;11:247-252.

Coelho M, Marti MJ, Tolosa E, et al. Late-stage Parkinson's disease: the Barcelona and Lisbon cohort. J Neurol 2010;257:1524-1532.

Comella CL, Nardine TM, Diederich NJ, Stebbins GT. Sleep-related violence, injury, and REM sleep behavior disorder in Parkinson's disease. Neurology 1998;51:526-529.

Compta Y, Santamaria J, Ratti L, et al. Cerebrospinal hypocretin, daytime sleepiness and sleep architecture in Parkinson's disease dementia. Brain 2009;132:3308-3317.

Cubo E, Benito-Leon J, Coronell C, Armesto D. Clinical correlates of apathy in patients recently diagnosed with Parkinson's disease: the ANIMO study. Neuroepidemiology 2012;38:48-55.

Damholdt MF, Borghammer P, Larsen L, Ostergaard K. Odor identification deficits identify Parkinson's disease patients with poor cognitive performance. Movement disorders : official journal of the Movement Disorder Society 2011;26:2045-2050.

De Cock VC, Vidailhet M, Leu S, et al. Restoration of normal motor control in Parkinson's disease during REM sleep. Brain 2007;130:450-456.

Defazio G, Berardelli A, Fabbrini G, et al. Pain as a nonmotor symptom of Parkinson disease: evidence from a case-control study. Arch Neurol 2008;65:1191-1194.

Di Giuda D, Camardese G, Bentivoglio AR, et al. Dopaminergic dysfunction and psychiatric symptoms in movement disorders: a (123)I-FP-CIT SPECT study. Eur J Nucl Med Mol Imaging 2012;39:1937-1948.

Dias FM, Kummer A, Doyle FC, et al. Psychiatric disorders in primary focal dystonia and in Parkinson's disease. Neuropsychiatr Dis Treat 2011;7:111-116.

Diederich NJ, Vaillant M, Mancuso G, Lyen P, Tiete J. Progressive sleep 'destructuring' in Parkinson's disease. A polysomnographic study in 46 patients. Sleep Med 2005;6:313-318.

Dissanayaka NN, Sellbach A, Matheson S, et al. Anxiety disorders in Parkinson's disease: prevalence and risk factors. Mov Disord 2010;25:838-845.

Dissanayaka NN, Sellbach A, Silburn PA, O'Sullivan JD, Marsh R, Mellick GD. Factors associated with depression in Parkinson's disease. J Affect Disord 2011;132:82-88.

Djaldetti R, Hassin-Baer S, Farrer MJ, et al. Clinical characteristics of Parkinson's disease among Jewish Ethnic groups in Israel. J Neural Transm 2008;115:1279-1284.

Doi H, Sakakibara R, Sato M, et al. Plasma levodopa peak delay and impaired gastric emptying in Parkinson's disease. J Neurol Sci 2012;319:86-88.

Dotchin CL, Jusabani A, Walker RW. Non-motor symptoms in a prevalent population with Parkinson's disease in Tanzania. Parkinsonism Relat Disord 2009;15:457-460.

Double KL, Rowe DB, Hayes M, et al. Identifying the pattern of olfactory deficits in Parkinson disease using the brief smell identification test. Archives of neurology 2003;60:545-549.

Drijgers RL, Dujardin K, Reijnders JS, Defebvre L, Leentjens AF. Validation of diagnostic criteria for apathy in Parkinson's disease. Parkinsonism Relat Disord 2010;16:656-660.

Eadie MJ, Tyrer JH. ALIMENTARY DISORDER IN PARKINSONISM. Australas Ann Med 1965;14:13-22.

Edwards LL, Pfeiffer RF, Quigley EM, Hofman R, Balluff M. Gastrointestinal symptoms in Parkinson's disease. Mov Disord 1991;6:151-156.

Edwards LL, Quigley EM, Harned RK, Hofman R, Pfeiffer RF. Defecatory function in Parkinson's disease: response to apomorphine. Ann Neurol 1993;33:490-493.

Edwards LL, Quigley EM, Harned RK, Hofman R, Pfeiffer RF. Characterization of swallowing and defecation in Parkinson's disease. Am J Gastroenterol 1994;89:15-25.

Ehrt U, Broich K, Larsen JP, Ballard C, Aarsland D. Use of drugs with anticholinergic effect and impact on cognition in Parkinson's disease: a cohort study. J Neurol Neurosurg Psychiatry 2010;81:160-165.

Erro R, Pappata S, Amboni M, et al. Anxiety is associated with striatal dopamine transporter availability in newly diagnosed untreated Parkinson's disease patients. Parkinsonism & related disorders 2012;18:1034-1038.

Erro R, Picillo M, Vitale C, et al. Non-motor symptoms in early Parkinson's disease: a 2-year follow-up study on previously untreated patients. Journal of neurology, neurosurgery, and psychiatry 2012.

Erro R, Santangelo G, Picillo M, et al. Link between non-motor symptoms and cognitive dysfunctions in de novo, drug-naive PD patients. Journal of neurology 2012;259:1808-1813.

Evans D, Norman P. Illness representations, coping and psychological adjustment to Parkinson's disease. Psychol Health 2009;24:1181-1196.

Fabbrini G, Barbanti P, Aurilia C, Vanacore N, Pauletti C, Meco G. Excessive daytime sleepiness in de novo and treated Parkinson's disease. Mov Disord 2002;17:1026-1030.

Factor SA, Steenland NK, Higgins DS, et al. Disease-related and genetic correlates of psychotic symptoms in Parkinson's disease. Mov Disord 2011.

Fang F, Xu Q, Park Y, et al. Depression and the subsequent risk of Parkinson's disease in the NIH-AARP Diet and Health Study. Mov Disord 2010;25:1157-1162.

Farabaugh AH, Locascio JJ, Yap L, et al. Assessing depression and factors possibly associated with depression during the course of Parkinson's disease. Ann Clin Psychiatry 2011;23:171-177.

Fernandez HH, See RH, Gary MF, et al. Depressive symptoms in Parkinson disease correlate with impaired global and specific cognitive performance. J Geriatr Psychiatry Neurol 2009;22:223-227.

Ferreira JJ, Desboeuf K, Galitzky M, et al. Sleep disruption, daytime somnolence and 'sleep attacks' in Parkinson's disease: a clinical survey in PD patients and age-matched healthy volunteers. Eur J Neurol 2006;13:209-214.

Ferri R, Fulda S, Cosentino FI, Pizza F, Plazzi G. A preliminary quantitative analysis of REM sleep chin EMG in Parkinson's disease with or without REM sleep behavior disorder. Sleep Med 2012;13:707-713.

Frauscher B, Hogl B, Maret S, et al. Association of daytime sleepiness with COMT polymorphism in patients with parkinson disease: a pilot study. Sleep 2004;27:733-736.

Fujiwara S, Kimura F, Hosokawa T, Ishida S, Sugino M, Hanafusa T. Anhedonia in Japanese patients with Parkinson's disease. Geriatr Gerontol Int 2011;11:275-281.

Furumoto H. Excessive daytime somnolence in Japanese patients with Parkinson's disease. Eur J Neurol 2004;11:535-540.

Gabrielli M, Bonazzi P, Scarpellini E, et al. Prevalence of small intestinal bacterial overgrowth in Parkinson's disease. Mov Disord 2011;26:889-892.

Gaenslen A, Swid I, Liepelt-Scarfone I, Godau J, Berg D. The patients' perception of prodromal symptoms before the initial diagnosis of Parkinson's disease. Mov Disord 2011;26:653-658.

Gagnon JF, Bedard MA, Fantini ML, et al. REM sleep behavior disorder and REM sleep without atonia in Parkinson's disease. Neurology 2002;59:585-589.

Gagnon JF, Fantini ML, Bedard MA, et al. Association between waking EEG slowing and REM sleep behavior disorder in PD without dementia. Neurology 2004;62:401-406.

Gallagher DA, Lees AJ, Schrag A. What are the most important nonmotor symptoms in patients with Parkinson's disease and are we missing them? Mov Disord 2010;25:2493-2500.

Garcia-Borreguero D, Caminero AB, De La Llave Y, et al. Decreased phasic EMG activity during rapid eye movement sleep in treatment-naive Parkinson's disease: effects of treatment with levodopa and progression of illness. Mov Disord 2002;17:934-941.

Gerbin M, Viner AS, Louis ED. Sleep in essential tremor: a comparison with normal controls and Parkinson's disease patients. Parkinsonism & related disorders 2012;18:279-284.

Ghorayeb I, Loundou A, Auquier P, Dauvilliers Y, Bioulac B, Tison F. A nationwide survey of excessive daytime sleepiness in Parkinson's disease in France. Mov Disord 2007;22:1567-1572.

Ghys L, Surmann E, Whitesides J, Boroojerdi B. Effect of rotigotine on sleep and quality of life in Parkinson's disease patients: post hoc analysis of RECOVER patients who were symptomatic at baseline. Expert opinion on pharmacotherapy 2011;12:1985-1998.

Giladi N, Treves TA, Paleacu D, et al. Risk factors for dementia, depression and psychosis in long-standing Parkinson's disease. J Neural Transm 2000;107:59-71.

Gjerstad MD, Alves G, Wentzel-Larsen T, Aarsland D, Larsen JP. Excessive daytime sleepiness in Parkinson disease: is it the drugs or the disease? Neurology 2006;67:853-858.

Go CL, Rosales RL, Joya-Tanglao M, Fernandez HH. Untreated depressive symptoms among cognitively-intact, community dwelling Filipino patients with Parkinson disease. Int J Neurosci 2011;121:137-141.

Goetz CG, Ouyang B, Negron A, Stebbins GT. Hallucinations and sleep disorders in PD: ten-year prospective longitudinal study. Neurology 2010;75:1773-1779.

Goldstein DS, Sewell L, Holmes C. Association of anosmia with autonomic failure in Parkinson disease. Neurology 2010;74:245-251.

Gomez-Esteban JC, Tijero B, Somme J, et al. Application of depression criteria (DSM-IV) in patients with Parkinson's disease. Clin Neurol Neurosurg 2009;111:665-669.

Gonera EG, van't Hof M, Berger HJ, van Weel C, Horstink MW. Symptoms and duration of the prodromal phase in Parkinson's disease. Mov Disord 1997;12:871-876.

Goulart FO, Godke BA, Borges V, et al. Fatigue in a cohort of geriatric patients with and without Parkinson's disease. Braz J Med Biol Res 2009;42:771-775.

Gupta A, Bhatia S. Psychological functioning in patients with Parkinson's disease. Parkinsonism Relat Disord 2000;6:185-190.

Haehner A, Boesveldt S, Berendse HW, et al. Prevalence of smell loss in Parkinson's disease--a multicenter study. Parkinsonism & related disorders 2009;15:490-494.

Hagell P, Broman JE. Measurement properties and hierarchical item structure of the Epworth Sleepiness Scale in Parkinson's disease. J Sleep Res 2007;16:102-109.

Han M, Ohnishi H, Nonaka M, et al. Relationship between dysphagia and depressive states in patients with Parkinson's disease. Parkinsonism Relat Disord 2011;17:437-439.

Hanna KK, Cronin-Golomb A. Impact of anxiety on quality of life in Parkinson's disease. Parkinsons Dis 2012;2012:640707.

Happe S, Schrodl B, Faltl M, Muller C, Auff E, Zeitlhofer J. Sleep disorders and depression in patients with Parkinson's disease. Acta Neurol Scand 2001;104:275-280.

Hatano T, Kubo SI, Shimo Y, Nishioka K, Hattori N. Unmet needs of patients with Parkinson's disease: interview survey of patients and caregivers. The Journal of international medical research 2009;37:717-726.

Havlikova E, van Dijk JP, Rosenberger J, et al. Fatigue in Parkinson's disease is not related to excessive sleepiness or quality of sleep. J Neurol Sci 2008;270:107-113.

Henderson JM, Lu Y, Wang S, Cartwright H, Halliday GM. Olfactory deficits and sleep disturbances in Parkinson's disease: a case-control survey. Journal of neurology, neurosurgery, and psychiatry 2003;74:956-958.

Henderson R, Kurlan R, Kersun JM, Como P. Preliminary examination of the comorbidity of anxiety and depression in Parkinson's disease. J Neuropsychiatry Clin Neurosci 1992;4:257-264.

Herlofson K, Ongre SO, Enger LK, Tysnes OB, Larsen JP. Fatigue in early Parkinson's disease. Minor inconvenience or major distress? Eur J Neurol 2012;19:963-968.

Herting B, Schulze S, Reichmann H, Haehner A, Hummel T. A longitudinal study of olfactory function in patients with idiopathic Parkinson's disease. Journal of neurology 2008;255:367-370.

Herzallah MM, Moustafa AA, Misk AJ, et al. Depression impairs learning whereas anticholinergics impair transfer generalization in Parkinson patients tested on dopaminergic medications. Cogn Behav Neurol 2010;23:98-105.

Hesse S, Meyer PM, Strecker K, et al. Monoamine transporter availability in Parkinson's disease patients with or without depression. Eur J Nucl Med Mol Imaging 2009;36:428-435.

Hinnell C, Hurt CS, Landau S, Brown RG, Samuel M. Nonmotor versus motor symptoms: how much do they matter to health status in Parkinson's disease? Movement disorders : official journal of the Movement Disorder Society 2012;27:236-241.

Hobson DE, Lang AE, Martin WR, Razmy A, Rivest J, Fleming J. Excessive daytime sleepiness and sudden-onset sleep in Parkinson disease: a survey by the Canadian Movement Disorders Group. JAMA : the journal of the American Medical Association 2002;287:455-463.

Hogl B, Seppi K, Brandauer E, et al. Increased daytime sleepiness in Parkinson's disease: a questionnaire survey. Mov Disord 2003;18:319-323.

Hu M, Cooper J, Beamish R, et al. How well do we recognise non-motor symptoms in a British Parkinson's disease population? J Neurol 2011;258:1513-1517.

Hummel T, Jahnke U, Sommer U, Reichmann H, Muller A. Olfactory function in patients with idiopathic Parkinson's disease: effects of deep brain stimulation in the subthalamic nucleus. Journal of neural transmission (Vienna, Austria : 1996) 2005;112:669-676.

Hurt CS, Landau S, Burn DJ, et al. Cognition, coping, and outcome in Parkinson's disease. International psychogeriatrics / IPA 2012;24:1656-1663.

Hurt CS, Weinman J, Lee R, Brown RG. The relationship of depression and disease stage to patient perceptions of Parkinson's disease. J Health Psychol 2012;17:1076-1088.

Ibarretxe-Bilbao N, Junque C, Marti MJ, et al. Olfactory impairment in Parkinson's disease and white matter abnormalities in central olfactory areas: A voxel-based diffusion tensor imaging study. Movement disorders : official journal of the Movement Disorder Society 2010;25:1888-1894.

Iijima M, Kobayakawa T, Saito S, et al. Smell identification in Japanese Parkinson's disease patients: using the odor stick identification test for Japanese subjects. Internal medicine (Tokyo, Japan) 2008;47:1887-1892.

Iijima M, Osawa M, Momose M, et al. Cardiac sympathetic degeneration correlates with olfactory function in Parkinson's disease. Movement disorders : official journal of the Movement Disorder Society 2010;25:1143-1149.

Imamura K, Okayasu N, Nagatsu T. The relationship between depression and regional cerebral blood flow in Parkinson's disease and the effect of selegiline treatment. Acta Neurol Scand 2011;124:28-39.

Inoue T, Kitagawa M, Tanaka T, Nakagawa S, Koyama T. Depression and major depressive disorder in patients with Parkinson's disease. Mov Disord 2010;25:44-49.

Ishihara-Paul L, Wainwright NW, Khaw KT, et al. Prospective association between emotional health and clinical evidence of Parkinson's disease. Eur J Neurol 2008;15:1148-1154.

Izawa MO, Miwa H, Kajimoto Y, Kondo T. Combination of transcranial sonography, olfactory testing, and MIBG myocardial scintigraphy as a diagnostic indicator for Parkinson's disease. European journal of neurology : the official journal of the European Federation of Neurological Societies 2012;19:411-416.

Jasinska-Myga B, Putzke JD, Wider C, Wszolek ZK, Uitti RJ. Depression in Parkinson's disease. Can J Neurol Sci 2010;37:61-66.

Johnson DK, Galvin JE. Longitudinal changes in cognition in Parkinson's disease with and without dementia. Dement Geriatr Cogn Disord 2011;31:98-108.

Jones CA, Pohar SL, Patten SB. Major depression and health-related quality of life in Parkinson's disease. Gen Hosp Psychiatry 2009;31:334-340.

Jost WH, Schrank B. Defecatory disorders in de novo Parkinsonians--colonic transit and electromyogram of the external anal sphincter. Wien Klin Wochenschr 1998;110:535-537.

Joutsa J, Martikainen K, Vahlberg T, Voon V, Kaasinen V. Impulse control disorders and depression in Finnish patients with Parkinson's disease. Parkinsonism Relat Disord 2012;18:155-160.

Kagi G, Klein C, Wood NW, et al. Nonmotor symptoms in Parkin gene-related parkinsonism. Mov Disord 2010;25:1279-1284.

Kashihara K, Ohno M, Kawada S, Imamura T. Frequent nocturnal vocalization in pure autonomic failure. The Journal of international medical research 2008;36:489-495.

Kasten M, Kertelge L, Tadic V, et al. Depression and quality of life in monogenic compared to idiopathic, early-onset Parkinson's disease. Mov Disord 2012;27:754-759.

Kaye J, Gage H, Kimber A, Storey L, Trend P. Excess burden of constipation in Parkinson's disease: a pilot study. Mov Disord 2006;21:1270-1273.

Khedr EM, El Fetoh NA, Khalifa H, Ahmed MA, El Beh KM. Prevalence of non motor features in a cohort of Parkinson's disease patients. Clinical neurology and neurosurgery 2012.

Kim HJ, Park SY, Cho YJ, et al. Nonmotor symptoms in de novo Parkinson disease before and after dopaminergic treatment. J Neurol Sci 2009;287:200-204.

Kim JS, Oh YS, Kim YI, Koo JS, Yang DW, Lee KS. Transcranial sonography (TCS) in Parkinson's disease (PD) and essential tremor (ET) in relation with putative premotor symptoms of PD. Archives of gerontology and geriatrics 2012;54:e436-439.

Kim JY, Lee WY, Chung EJ, Dhong HJ. Analysis of olfactory function and the depth of olfactory sulcus in patients with Parkinson's disease. Movement disorders : official journal of the Movement Disorder Society 2007;22:1563-1566.

Kirsch-Darrow L, Marsiske M, Okun MS, Bauer R, Bowers D. Apathy and depression: separate factors in Parkinson's disease. J Int Neuropsychol Soc 2011;17:1058-1066.

Klotsche J, Reese JP, Winter Y, et al. Trajectory classes of decline in health-related quality of life in Parkinson's disease: a pilot study. Value Health 2011;14:329-338.

Koerts J, van Beilen M, Leenders KL, Brouwer WH, Tucha L, Tucha O. Complaints about impairments in executive functions in Parkinson's disease: the association with neuropsychological assessment. Parkinsonism Relat Disord 2012;18:194-197.

Kostic VS, Susic V, Przedborski S, Sternic N. Sleep EEG in depressed and nondepressed patients with Parkinson's disease. J Neuropsychiatry Clin Neurosci 1991;3:176-179.

Kotagal V, Albin RL, Muller ML, et al. Symptoms of rapid eye movement sleep behavior disorder are associated with cholinergic denervation in Parkinson disease. Ann Neurol 2012;71:560-568.

Krogh K, Ostergaard K, Sabroe S, Laurberg S. Clinical aspects of bowel symptoms in Parkinson's disease. Acta Neurol Scand 2008;117:60-64.

Kulisevsky J, Pagonabarraga J, Pascual-Sedano B, Garcia-Sanchez C, Gironell A. Prevalence and correlates of neuropsychiatric symptoms in Parkinson's disease without dementia. Mov Disord 2008;23:1889-1896.

Kumar S, Bhatia M, Behari M. Excessive daytime sleepiness in Parkinson's disease as assessed by Epworth Sleepiness Scale (ESS). Sleep Med 2003;4:339-342.

Kummer A, Cardoso F, Teixeira AL. Generalized anxiety disorder and the Hamilton Anxiety Rating Scale in Parkinson's disease. Arq Neuropsiquiatr 2010;68:495-501.

Kummer A, Harsanyi E, Dias FM, Cardoso F, Caramelli P, Teixeira AL. Depression impairs executive functioning in Parkinson disease patients with low educational level. Cogn Behav Neurol 2009;22:167-172.

Larsen JP, Karlsen K, Tandberg E. Clinical problems in non-fluctuating patients with Parkinson's disease: a community-based study. Mov Disord 2000;15:826-829.

Lavault S, Leu-Semenescu S, Tezenas du Montcel S, Cochen de Cock V, Vidailhet M, Arnulf I. Does clinical rapid eye movement behavior disorder predict worse outcomes in Parkinson's disease? J Neurol 2010;257:1154-1159.

Lee AH, Weintraub D. Psychosis in Parkinson's disease without dementia: common and comorbid with other non-motor symptoms. Movement disorders : official journal of the Movement Disorder Society 2012;27:858-863.

Lee JE, Kim KS, Shin HW, Sohn YH. Factors related to clinically probable REM sleep behavior disorder in Parkinson disease. Parkinsonism Relat Disord 2010;16:105-108.

Lee MA, Prentice WM, Hildreth AJ, Walker RW. Measuring symptom load in Idiopathic Parkinson's disease. Parkinsonism Relat Disord 2007;13:284-289.

Leentjens AF, Dujardin K, Marsh L, Richard IH, Starkstein SE, Martinez-Martin P. Anxiety rating scales in Parkinson's disease: a validation study of the Hamilton anxiety rating scale, the Beck anxiety inventory, and the hospital anxiety and depression scale. Mov Disord 2011;26:407-415.

Leiknes I, Tysnes OB, Aarsland D, Larsen JP. Caregiver distress associated with neuropsychiatric problems in patients with early Parkinson's disease: the Norwegian ParkWest study. Acta Neurol Scand 2010;122:418-424.

Leroi I, Pantula H, McDonald K, Harbishettar V. Neuropsychiatric symptoms in Parkinson's disease with mild cognitive impairment and dementia. Parkinsons Dis 2012;2012:308097.

Letro GH, Quagliato EM, Viana MA. Pain in Parkinson's disease. Arq Neuropsiquiatr 2009;67:591-594.

Li W, Liu J, Skidmore F, Liu Y, Tian J, Li K. White matter microstructure changes in the thalamus in Parkinson disease with depression: A diffusion tensor MR imaging study. AJNR Am J Neuroradiol 2010;31:1861-1866.

Liu AA, Boxhorn CE, Klufas MA, et al. Clinical predictors of frequent patient telephone calls in Parkinson's disease. Parkinsonism & related disorders 2011;17:95-99.

Lo RY, Tanner CM, Albers KB, et al. Clinical features in early Parkinson disease and survival. Arch Neurol 2009;66:1353-1358.

Margis R, Donis KC, Schonwald SV, Rieder CR. WHOQOL-OLD assessment of quality of life in elderly patients with Parkinson's disease: influence of sleep and depressive symptoms. Revista brasileira de psiquiatria (Sao Paulo, Brazil : 1999) 2010;32:125-131.

Marinus J, Visser M, van Hilten JJ, Lammers GJ, Stiggelbout AM. Assessment of sleep and sleepiness in Parkinson disease. Sleep 2003;26:1049-1054.

Marion MH, Qurashi M, Marshall G, Foster O. Is REM sleep behaviour disorder (RBD) a risk factor of dementia in idiopathic Parkinson's disease? J Neurol 2008;255:192-196.

Martinez-Martin P, Falup Pecurariu C, Odin P, et al. Gender-related differences in the burden of non-motor symptoms in Parkinson's disease. Journal of neurology 2012;259:1639-1647.

Martinez-Martin P, Rodriguez-Blazquez C, Kurtis MM, Chaudhuri KR. The impact of non-motor symptoms on health-related quality of life of patients with Parkinson's disease. Mov Disord 2011;26:399-406.

Martinez-Martin P, Schapira AH, Stocchi F, et al. Prevalence of nonmotor symptoms in Parkinson's disease in an international setting; study using nonmotor symptoms questionnaire in 545 patients. Mov Disord 2007;22:1623-1629.

Matheson SF, Byrne GJ, Dissanayaka NN, et al. Validity and reliability of the Geriatric Anxiety Inventory in Parkinson's disease. Australas J Ageing 2012;31:13-16.

Matuja WB, Aris EA. Motor and non-motor features of Parkinson's disease. East Afr Med J 2008;85:3-9.

Medeiros CA, Carvalhedo de Bruin PF, Lopes LA, Magalhaes MC, de Lourdes Seabra M, de Bruin VM. Effect of exogenous melatonin on sleep and motor dysfunction in Parkinson's disease. A randomized, double blind, placebo-controlled study. J Neurol 2007;254:459-464.

Meral H, Aydemir T, Ozer F, et al. Relationship between visual hallucinations and REM sleep behavior disorder in patients with Parkinson's disease. Clin Neurol Neurosurg 2007;109:862-867.

Meusel T, Westermann B, Fuhr P, Hummel T, Welge-Lussen A. The course of olfactory deficits in patients with Parkinson's disease--a study based on psychophysical and electrophysiological measures. Neuroscience letters 2010;486:166-170.

Millar Vernetti P, Perez Lloret S, Rossi M, Cerquetti D, Merello M. Validation of a new scale to assess olfactory dysfunction in patients with Parkinson's disease. Parkinsonism & related disorders 2012;18:358-361.

Miwa H, Miwa T. Fatigue in patients with Parkinson's disease: impact on quality of life. Intern Med 2011;50:1553-1558.

Monaca C, Duhamel A, Jacquesson JM, et al. Vigilance troubles in Parkinson's disease: a subjective and objective polysomnographic study. Sleep Med 2006;7:448-453.

Montel S, Bonnet AM, Bungener C. Quality of life in relation to mood, coping strategies, and dyskinesia in Parkinson's disease. J Geriatr Psychiatry Neurol 2009;22:95-102.

Moreno-Lopez C, Santamaria J, Salamero M, et al. Excessive daytime sleepiness in multiple system atrophy (SLEEMSA study). Arch Neurol 2011;68:223-230.

Morgante L, Colosimo C, Antonini A, et al. Psychosis associated to Parkinson's disease in the early stages: relevance of cognitive decline and depression. J Neurol Neurosurg Psychiatry 2011.

Morgante L, Colosimo C, Antonini A, et al. Psychosis associated to Parkinson's disease in the early stages: relevance of cognitive decline and depression. Journal of neurology, neurosurgery, and psychiatry 2012;83:76-82.

Morley JF, Weintraub D, Mamikonyan E, Moberg PJ, Siderowf AD, Duda JE. Olfactory dysfunction is associated with neuropsychiatric manifestations in Parkinson's disease. Mov Disord 2011.

Muller A, Reichmann H, Livermore A, Hummel T. Olfactory function in idiopathic Parkinson's disease (IPD): results from cross-sectional studies in IPD patients and long-term follow-up of de-novo IPD patients. Journal of neural transmission (Vienna, Austria : 1996) 2002;109:805-811.

Muller B, Larsen JP, Wentzel-Larsen T, Skeie GO, Tysnes OB. Autonomic and sensory symptoms and signs in incident, untreated Parkinson's disease: frequent but mild. Mov Disord 2011;26:65-72.

Munhoz RP, Werneck LC, Teive HA. The differential diagnoses of parkinsonism: findings from a cohort of 1528 patients and a 10 years comparison in tertiary movement disorders clinics. Clinical neurology and neurosurgery 2010;112:431-435.

Naismith SL, Rogers NL, Mackenzie J, Hickie IB, Lewis SJ. The relationship between actigraphically defined sleep disturbance and REM sleep behaviour disorder in Parkinson's Disease. Clin Neurol Neurosurg 2010;112:420-423.

Nation DA, Katzen HL, Papapetropoulos S, Scanlon BK, Levin BE. Subthreshold depression in Parkinson's disease. Int J Geriatr Psychiatry 2009;24:937-943.

Nazzaro JM, Pahwa R, Lyons KE. The impact of bilateral subthalamic stimulation on non-motor symptoms of Parkinson's disease. Parkinsonism & related disorders 2011;17:606-609.

Negre-Pages L, Grandjean H, Lapeyre-Mestre M, et al. Anxious and depressive symptoms in Parkinson's disease: the French cross-sectionnal DoPaMiP study. Mov Disord 2010;25:157-166.

Nihei Y, Takahashi K, Koto A, et al. REM sleep behavior disorder in Japanese patients with Parkinson's disease: a multicenter study using the REM sleep behavior disorder screening questionnaire. Journal of neurology 2012;259:1606-1612.

Nomura T, Inoue Y, Hogl B, et al. Comparison of the clinical features of rapid eye movement sleep behavior disorder in patients with Parkinson's disease and multiple system atrophy. Psychiatry Clin Neurosci 2011;65:264-271.

Nomura T, Inoue Y, Kagimura T, Uemura Y, Nakashima K. Utility of the REM sleep behavior disorder screening questionnaire (RBDSQ) in Parkinson's disease patients. Sleep Med 2011;12:711-713.

Nomura T, Inoue Y, Takigawa H, Nakashima K. Comparison of REM sleep behaviour disorder variables between patients with progressive supranuclear palsy and those with Parkinson's disease. Parkinsonism Relat Disord 2012;18:394-396.

Oberholzer M, Poryazova R, Bassetti CL. Sleepwalking in Parkinson's disease: a questionnaire-based survey. J Neurol 2011;258:1261-1267.

Oguru M, Tachibana H, Toda K, Okuda B, Oka N. Apathy and depression in Parkinson disease. J Geriatr Psychiatry Neurol 2010;23:35-41.

Ondo WG, Shinawi L, Davidson A, Lai D. Memantine for non-motor features of Parkinson's disease: a double-blind placebo controlled exploratory pilot trial. Parkinsonism & related disorders 2011;17:156-159.

Onofrj M, Thomas A, D'Andreamatteo G, et al. Incidence of RBD and hallucination in patients affected by Parkinson's disease: 8-year follow-up. Neurol Sci 2002;23 Suppl 2:S91-94.

Onofrj M, Thomas A, Tiraboschi P, et al. Updates on Somatoform Disorders (SFMD) in Parkinson's Disease and Dementia with Lewy Bodies and discussion of phenomenology. J Neurol Sci 2011.

O'Suilleabhain PE, Dewey RB, Jr. Contributions of dopaminergic drugs and disease severity to daytime sleepiness in Parkinson disease. Arch Neurol 2002;59:986-989.

O'Sullivan SS, Loane CM, Lawrence AD, Evans AH, Piccini P, Lees AJ. Sleep disturbance and impulsive-compulsive behaviours in Parkinson's disease. J Neurol Neurosurg Psychiatry 2011;82:620-622.

O'Sullivan SS, Williams DR, Gallagher DA, Massey LA, Silveira-Moriyama L, Lees AJ. Nonmotor symptoms as presenting complaints in Parkinson's disease: a clinicopathological study. Mov Disord 2008;23:101-106.

Oudiette D, De Cock VC, Lavault S, Leu S, Vidailhet M, Arnulf I. Nonviolent elaborate behaviors may also occur in REM sleep behavior disorder. Neurology 2009;72:551-557.

Pacchetti C, Manni R, Zangaglia R, et al. A questionnaire on sleep and mental disorders in Parkinson's disease (QSMDPD): development and application of a new screening tool. Functional neurology 2004;19:83-99.

Pacchetti C, Manni R, Zangaglia R, et al. Relationship between hallucinations, delusions, and rapid eye movement sleep behavior disorder in Parkinson's disease. Mov Disord 2005;20:1439-1448.

Parma V, Bulgheroni M, Scaravilli T, Tirindelli R, Castiello U. Implicit olfactory processing attenuates motor disturbances in idiopathic Parkinson's disease. Cortex; a journal devoted to the study of the nervous system and behavior 2012.

Pedersen KF, Alves G, Aarsland D, Larsen JP. Occurrence and risk factors for apathy in Parkinson disease: a 4-year prospective longitudinal study. J Neurol Neurosurg Psychiatry 2009;80:1279-1282.

Pedersen KF, Alves G, Bronnick K, Aarsland D, Tysnes OB, Larsen JP. Apathy in drug-naive patients with incident Parkinson's disease: the Norwegian ParkWest study. J Neurol 2010;257:217-223.

Peralta CM, Frauscher B, Seppi K, et al. Restless legs syndrome in Parkinson's disease. Mov Disord 2009;24:2076-2080.

Perez Lloret S, Rossi M, Cardinali DP, Merello M. Validation of the sleep related items of the Non-motor Symptoms Questionnaire for Parkinson's disease (NMSQuest). Parkinsonism & related disorders 2008;14:641-645.

Phuong L, Garg S, Duda JE, Stern MB, Weintraub D. Involuntary emotional expression disorder (IEED) in Parkinson's disease. Parkinsonism Relat Disord 2009;15:511-515.

Piccinni A, Marazziti D, Veltri A, et al. Depressive symptoms in Parkinson's disease. Compr Psychiatry 2012;53:727-731.

Pollak L, Prohorov T, Kushnir M, Rabey M. Vestibulocervical reflexes in idiopathic Parkinson disease. Neurophysiol Clin 2009;39:235-240.

Pontone GM, Williams JR, Anderson KE, et al. Anxiety and self-perceived health status in Parkinson's disease. Parkinsonism Relat Disord 2011;17:249-254.

Poryazova R, Benninger D, Waldvogel D, Bassetti CL. Excessive daytime sleepiness in Parkinson's disease: characteristics and determinants. Eur Neurol 2010;63:129-135.

Postuma RB, Gagnon JF, Vendette M, Charland K, Montplaisir J. REM sleep behaviour disorder in Parkinson's disease is associated with specific motor features. J Neurol Neurosurg Psychiatry 2008;79:1117-1121.

Postuma RB, Montplaisir J, Lanfranchi P, et al. Cardiac autonomic denervation in Parkinson's disease is linked to REM sleep behavior disorder. Mov Disord 2011;26:1529-1533.

Potagas C, Dellatolas G, Ziegler M, et al. Clinical assessment of olfactory dysfunction in Parkinson's disease. Movement disorders : official journal of the Movement Disorder Society 1998;13:394-399.

Qin Z, Zhang L, Sun F, et al. Health related quality of life in early Parkinson's disease: impact of motor and non-motor symptoms, results from Chinese levodopa exposed cohort. Parkinsonism & related disorders 2009;15:767-771.

Qin Z, Zhang L, Sun F, Liu H, Fang X, Chan P. Depressive symptoms impacting on health-related quality of life in early Parkinson's disease: results from Chinese L-dopa exposed cohort. Clin Neurol Neurosurg 2009;111:733-737.

Quelhas R, Costa M. Anxiety, depression, and quality of life in Parkinson's disease. J Neuropsychiatry Clin Neurosci 2009;21:413-419.

Raggi A, Leonardi M, Carella F, Soliveri P, Albanese A, Romito LM. Impact of nonmotor symptoms on disability in patients with Parkinson's disease. International journal of rehabilitation research Internationale Zeitschrift fur Rehabilitationsforschung Revue internationale de recherches de readaptation 2011;34:316-320.

Ramjit AL, Sedig L, Leibner J, et al. The relationship between anosmia, constipation, and orthostasis and Parkinson's disease duration: results of a pilot study. The International journal of neuroscience 2010;120:67-70.

Ratti PL, Terzaghi M, Minafra B, et al. REM and NREM sleep enactment behaviors in Parkinson's disease, Parkinson's disease dementia, and dementia with Lewy bodies. Sleep Med 2012;13:926-932.

Ravina B, Camicioli R, Como PG, et al. The impact of depressive symptoms in early Parkinson disease. Neurology 2007;69:342-347.

Ravina B, Tanner C, Dieuliis D, et al. A longitudinal program for biomarker development in Parkinson's disease: a feasibility study. Movement disorders : official journal of the Movement Disorder Society 2009;24:2081-2090.

Razmy A, Lang AE, Shapiro CM. Predictors of impaired daytime sleep and wakefulness in patients with Parkinson disease treated with older (ergot) vs newer (nonergot) dopamine agonists. Arch Neurol 2004;61:97-102.

Ready RE, Friedman J, Grace J, Fernandez H. Testosterone deficiency and apathy in Parkinson's disease: a pilot study. J Neurol Neurosurg Psychiatry 2004;75:1323-1326.

Reiff J, Schmidt N, Riebe B, et al. Subthreshold Depression in Parkinson's Disease. Mov Disord 2011.

Reijnders JS, Lousberg R, Leentjens AF. Assessment of depression in Parkinson's disease: the contribution of somatic symptoms to the clinimetric performance of the Hamilton and Montgomery-Asberg rating scales. J Psychosom Res 2010;68:561-565.

Richard IH, Kurlan R. The under-recognition of depression in Parkinson's disease. Neuropsychiatr Dis Treat 2006;2:349-353.

Richard IH, LaDonna KA, Hartman R, Podgorski C, Kurlan R. The patients' perspective: Results of a survey assessing knowledge about and attitudes toward depression in PD. Neuropsychiatr Dis Treat 2007;3:903-906.

Riedel O, Dodel R, Deuschl G, et al. Depression and care-dependency in Parkinson's disease: results from a nationwide study of 1449 outpatients. Parkinsonism Relat Disord 2012;18:598-601.

Riedel O, Klotsche J, Spottke A, et al. Frequency of dementia, depression, and other neuropsychiatric symptoms in 1,449 outpatients with Parkinson's disease. J Neurol 2010;257:1073-1082.

Rodriguez-Violante M, Cervantes-Arriaga A, Berlanga-Flores C, Ruiz-Chow A. Prevalence and determinants of depression in Mexican patients with Parkinson's disease. Clinical neurology and neurosurgery 2012;114:1293-1296.

Rodriguez-Violante M, Cervantes-Arriaga A, Villar-Velarde A, Corona T. Relationship between the type and side of motor symptoms with the prevalence of non-motor symptoms in Parkinson's disease. Neurologia 2011;26:319-324.

Romenets SR, Gagnon JF, Latreille V, et al. Rapid eye movement sleep behavior disorder and subtypes of Parkinson's disease. Movement disorders : official journal of the Movement Disorder Society 2012;27:996-1003.

Romenets SR, Wolfson C, Galatas C, et al. Validation of the non-motor symptoms questionnaire (NMS-Quest). Parkinsonism & related disorders 2012;18:54-58.

Ross GW, Petrovitch H, Abbott RD, et al. Association of olfactory dysfunction with risk for future Parkinson's disease. Ann Neurol 2008;63:167-173.

Ruiz-Martinez J, Gorostidi A, Goyenechea E, et al. Olfactory deficits and cardiac (123) I-MIBG in Parkinson's disease related to the LRRK2 R1441G and G2019S mutations. Mov Disord 2011.

Sakakibara R, Odaka T, Uchiyama T, et al. Colonic transit time and rectoanal videomanometry in Parkinson's disease. J Neurol Neurosurg Psychiatry 2003;74:268-272.

Sanchez-Ferro A, Benito-Leon J, Mitchell AJ, et al. Premotor cognitive status in a cohort of incident Parkinson disease patients (NEDICES). J Neurol Sci 2011.

Santangelo G, Vitale C, Trojano L, et al. Relationship between depression and cognitive dysfunctions in Parkinson's disease without dementia. J Neurol 2009;256:632-638.

Saunders-Pullman R, Stanley K, Wang C, et al. Olfactory dysfunction in LRRK2 G2019S mutation carriers. Neurology 2011;77:319-324.

Savica R, Carlin JM, Grossardt BR, et al. Medical records documentation of constipation preceding Parkinson disease: A case-control study. Neurology 2009;73:1752-1758.

Scaglione C, Vignatelli L, Plazzi G, et al. REM sleep behaviour disorder in Parkinson's disease: a questionnaire-based study. Neurol Sci 2005;25:316-321.

Scalzo P, Kummer A, Cardoso F, Teixeira AL. Depressive symptoms and perception of quality of life in Parkinson's disease. Arq Neuropsiquiatr 2009;67:203-208.

Schlesinger I, Ravin PD. Dopamine agonists induce episodes of irresistible daytime sleepiness. Eur Neurol 2003;49:30-33.

Schneider CB, Pilhatsch M, Rifati M, et al. Utility of the WHO-Five Well-being Index as a screening tool for depression in Parkinson's disease. Mov Disord 2010;25:777-783.

Schneider JS, Elm JJ, Parashos SA, Ravina BM, Galpern WR. Predictors of cognitive outcomes in early Parkinson disease patients: The National Institutes of Health Exploratory Trials in Parkinson Disease (NET-PD) experience. Parkinsonism Relat Disord 2010;16:507-512.

Schrag A, Ben-Shlomo Y, Quinn N. How common are complications of Parkinson's disease? J Neurol 2002;249:419-423.

Shah M, Deeb J, Fernando M, et al. Abnormality of taste and smell in Parkinson's disease. Parkinsonism Relat Disord 2009;15:232-237.

Sharma JC, Turton J. Olfaction, dyskinesia and profile of weight change in Parkinson's disease: identifying neurodegenerative phenotypes. Parkinsonism & related disorders 2012;18:964-970.

Shearer J, Green C, Counsell CE, Zajicek JP. The impact of motor and non motor symptoms on health state values in newly diagnosed idiopathic Parkinson's disease. Journal of neurology 2012;259:462-468.

Shiba M, Bower JH, Maraganore DM, et al. Anxiety disorders and depressive disorders preceding Parkinson's disease: a case-control study. Mov Disord 2000;15:669-677.

Shin HY, Joo EY, Kim ST, Dhong HJ, Cho JW. Comparison study of olfactory function and substantia nigra hyperechogenicity in idiopathic REM sleep behavior disorder, Parkinson's disease and normal control. Neurological sciences : official journal of the Italian Neurological Society and of the Italian Society of Clinical Neurophysiology 2012.

Shine JM, Naismith SL, Lewis SJ. The differential yet concurrent contributions of motor, cognitive and affective disturbance to freezing of gait in Parkinson's disease. Clin Neurol Neurosurg 2012.

Shpirer I, Miniovitz A, Klein C, et al. Excessive daytime sleepiness in patients with Parkinson's disease: a polysomnography study. Mov Disord 2006;21:1432-1438.

Shulman LM, Taback RL, Bean J, Weiner WJ. Comorbidity of the nonmotor symptoms of Parkinson's disease. Mov Disord 2001;16:507-510.

Shulman LM, Taback RL, Rabinstein AA, Weiner WJ. Non-recognition of depression and other non-motor symptoms in Parkinson's disease. Parkinsonism & related disorders 2002;8:193-197.

Sinforiani E, Zangaglia R, Manni R, et al. REM sleep behavior disorder, hallucinations, and cognitive impairment in Parkinson's disease. Mov Disord 2006;21:462-466.

Singer C, Weiner WJ, Sanchez-Ramos JR. Autonomic dysfunction in men with Parkinson's disease. Eur Neurol 1992;32:134-140.

Siri C, Cilia R, De Gaspari D, et al. Psychiatric symptoms in Parkinson's disease assessed with the SCL-90R self-reported questionnaire. Neurol Sci 2010;31:35-40.

Sixel-Doring F, Schweitzer M, Mollenhauer B, Trenkwalder C. Polysomnographic findings, video-based sleep analysis and sleep perception in progressive supranuclear palsy. Sleep Med 2009;10:407-415.

Skeie GO, Muller B, Haugarvoll K, Larsen JP, Tysnes OB. Parkinson disease: Associated disorders in the Norwegian population based incident ParkWest study. Parkinsonism Relat Disord 2012.

Solla P, Cannas A, Floris GL, et al. Behavioral, neuropsychiatric and cognitive disorders in Parkinson's disease patients with and without motor complications. Prog Neuropsychopharmacol Biol Psychiatry 2011;35:1009-1013.

Solla P, Cannas A, Ibba FC, et al. Gender differences in motor and non-motor symptoms among Sardinian patients with Parkinson's disease. Journal of the neurological sciences 2012;323:33-39.

Spica V, Pekmezovic T, Svetel M, Kostic VS. Prevalence of non-motor symptoms in young-onset versus late-onset Parkinson's disease. Journal of neurology 2012.

Starkstein S, Dragovic M, Jorge R, et al. Diagnostic criteria for depression in Parkinson's disease: a study of symptom patterns using latent class analysis. Mov Disord 2011;26:2239-2245.

Stella F, Banzato CE, Quagliato EM, Viana MA, Christofoletti G. Psychopathological features in patients with Parkinson's disease and related caregivers' burden. Int J Geriatr Psychiatry 2009;24:1158-1165.

Stern Y, Marder K, Tang MX, Mayeux R. Antecedent clinical features associated with dementia in Parkinson's disease. Neurology 1993;43:1690-1692.

Stevens S, Cormella CL, Stepanski EJ. Daytime sleepiness and alertness in patients with Parkinson disease. Sleep 2004;27:967-972.

Strutt AM, Simpson R, Jankovic J, York MK. Changes in cognitive-emotional and physiological symptoms of depression following STN-DBS for the treatment of Parkinson's disease. European journal of neurology : the official journal of the European Federation of Neurological Societies 2012;19:121-127.

Sung HY, Choi MG, Kim YI, Lee KS, Kim JS. Anorectal manometric dysfunctions in newly diagnosed, early-stage Parkinson's disease. Journal of clinical neurology (Seoul, Korea) 2012;8:184-189.

Surdhar I, Gee M, Bouchard T, Coupland N, Malykhin N, Camicioli R. Intact limbic-prefrontal connections and reduced amygdala volumes in Parkinson's disease with mild depressive symptoms. Parkinsonism Relat Disord 2012;18:809-813.

Suzuki K, Miyamoto T, Miyamoto M, et al. Excessive daytime sleepiness and sleep episodes in Japanese patients with Parkinson's disease. J Neurol Sci 2008;271:47-52.

Suzuki M, Hashimoto M, Yoshioka M, Murakami M, Kawasaki K, Urashima M. The odor stick identification test for Japanese differentiates Parkinson's disease from multiple system atrophy and progressive supra nuclear palsy. BMC neurology 2011;11:157.

Svensson E, Beiske AG, Loge JH, Beiske KK, Sivertsen B. Sleep problems in Parkinson's disease: a community-based study in Norway. BMC neurology 2012;12:71.

Tan EK, Lum SY, Fook-Chong SM, et al. Evaluation of somnolence in Parkinson's disease: comparison with age- and sex-matched controls. Neurology 2002;58:465-468.

Tandberg E, Larsen JP, Karlsen K. Excessive daytime sleepiness and sleep benefit in Parkinson's disease: a community-based study. Mov Disord 1999;14:922-927.

Tateno F, Sakakibara R, Yokoi Y, et al. Levodopa ameliorated anorectal constipation in de novo Parkinson's disease: The QL-GAT study. Parkinsonism Relat Disord 2011;17:662-666.

Thompson AW, Liu H, Hays RD, et al. Diagnostic accuracy and agreement across three depression assessment measures for Parkinson's disease. Parkinsonism Relat Disord 2011;17:40-45.

Tissingh G, Berendse HW, Bergmans P, et al. Loss of olfaction in de novo and treated Parkinson's disease: possible implications for early diagnosis. Movement disorders : official journal of the Movement Disorder Society 2001;16:41-46.

Tremblay C, Monchi O, Hudon C, Macoir J, Monetta L. Are verbal fluency and nonliteral language comprehension deficits related to depressive symptoms in Parkinson's disease? Parkinsons Dis 2012;2012:308501.

Ueki A, Otsuka M. Life style risks of Parkinson's disease: association between decreased water intake and constipation. J Neurol 2004;251 Suppl 7:vII18-23.

Valko PO, Waldvogel D, Weller M, Bassetti CL, Held U, Baumann CR. Fatigue and excessive daytime sleepiness in idiopathic Parkinson's disease differently correlate with motor symptoms, depression and dopaminergic treatment. Eur J Neurol 2010;17:1428-1436.

van der Hoek TC, Bus BA, Matui P, van der Marck MA, Esselink RA, Tendolkar I. Prevalence of depression in Parkinson's disease: Effects of disease stage, motor subtype and gender. J Neurol Sci 2011.

Vanderheyden JE, Gonce M, Bourgeois P, et al. Epidemiology of major depression in Belgian parkinsonian patients. Acta Neurol Belg 2010;110:148-156.

Veazey C, Cook KF, Stanley M, Lai EC, Kunik ME. Telephone-administered cognitive behavioral therapy: a case study of anxiety and depression in Parkinson's disease. J Clin Psychol Med Settings 2009;16:243-253.

Veiga BA, Borges V, Silva SM, Goulart Fde O, Cendoroglo MS, Ferraz HB. Depression in Parkinson's disease: clinical-epidemiological correlates and comparison with a controlled group of non-parkinsonian geriatric patients. Rev Bras Psiquiatr 2009;31:39-42.

Velez Feijo A, Rieder CR, Chaves ML. Did depressive symptoms affect recognition of emotional prosody in Parkinson's disease? Neuropsychiatr Dis Treat 2008;4:669-674.

Verbaan D, Boesveldt S, van Rooden SM, et al. Is olfactory impairment in Parkinson disease related to phenotypic or genotypic characteristics? Neurology 2008;71:1877-1882.

Verbaan D, Marinus J, Visser M, van Rooden SM, Stiggelbout AM, van Hilten JJ. Patient-reported autonomic symptoms in Parkinson disease. Neurology 2007;69:333-341.

Verbaan D, van Rooden SM, Visser M, Marinus J, van Hilten JJ. Nighttime sleep problems and daytime sleepiness in Parkinson's disease. Mov Disord 2008;23:35-41.

Vibha D, Shukla G, Goyal V, Singh S, Srivastava AK, Behari M. RBD in Parkinson's disease: a clinical case control study from North India. Clin Neurol Neurosurg 2011;113:472-476.

Vibha D, Shukla G, Singh S, Goyal V, Srivastava AK, Behari M. Lower prevalence of sleep disturbances in familial versus sporadic Parkinson's disease: a questionnaire based study. J Neurol Sci 2010;295:27-30.

Wallace DM, Shafazand S, Carvalho DZ, et al. Sleep-related falling out of bed in Parkinson's disease. J Clin Neurol 2012;8:51-57.

Wang G, Wan Y, Cheng Q, et al. Malnutrition and associated factors in Chinese patients with Parkinson's disease: Results from a pilot investigation. Parkinsonism & related disorders 2010;16:119-123.

Wang G, Wan Y, Wang Y, et al. Visual hallucinations and associated factors in Chinese patients with Parkinson's disease: roles of RBD and visual pathway deficit. Parkinsonism Relat Disord 2010;16:695-696.

Wang SJ, Fuh JL, Shan DE, et al. Sympathetic skin response and R-R interval variation in Parkinson's disease. Mov Disord 1993;8:151-157.

Ward CD, Hess WA, Calne DB. Olfactory impairment in Parkinson's disease. Neurology 1983;33:943-946.

Wei YJ, Stuart B, Zuckerman IH. Use of antiparkinson medications among elderly Medicare beneficiaries with Parkinson's disease. Am J Geriatr Pharmacother 2010;8:384-394.

Weintraub D, Moberg PJ, Duda JE, Katz IR, Stern MB. Effect of psychiatric and other nonmotor symptoms on disability in Parkinson's disease. J Am Geriatr Soc 2004;52:784-788.

Welge-Lussen A, Wattendorf E, Schwerdtfeger U, et al. Olfactory-induced brain activity in Parkinson's disease relates to the expression of event-related potentials: a functional magnetic resonance imaging study. Neuroscience 2009;162:537-543.

Wetter TC, Collado-Seidel V, Pollmacher T, Yassouridis A, Trenkwalder C. Sleep and periodic leg movement patterns in drug-free patients with Parkinson's disease and multiple system atrophy. Sleep 2000;23:361-367.

Wetter TC, Trenkwalder C, Gershanik O, Hogl B. Polysomnographic measures in Parkinson's disease: a comparison between patients with and without REM sleep disturbances. Wien Klin Wochenschr 2001;113:249-253.

Winter Y, von Campenhausen S, Arend M, et al. Health-related quality of life and its determinants in Parkinson's disease: results of an Italian cohort study. Parkinsonism Relat Disord 2011;17:265-269.

Witjas T, Kaphan E, Azulay JP, et al. Nonmotor fluctuations in Parkinson's disease: frequent and disabling. Neurology 2002;59:408-413.

Wolz M, Kaminsky A, Lohle M, Koch R, Storch A, Reichmann H. Chocolate consumption is increased in Parkinson's disease. Results from a self-questionnaire study. J Neurol 2009;256:488-492.

Wu X, Yu C, Fan F, et al. Correlation between progressive changes in piriform cortex and olfactory performance in early Parkinson's disease. European neurology 2011;66:98-105.

Yong MH, Fook-Chong S, Pavanni R, Lim LL, Tan EK. Case control polysomnographic studies of sleep disorders in Parkinson's disease. PLoS One 2011;6:e22511.

Yoritaka A, Ohizumi H, Tanaka S, Hattori N. Parkinson's disease with and without REM sleep behaviour disorder: are there any clinical differences? Eur Neurol 2009;61:164-170.

Young A, Home M, Churchward T, Freezer N, Holmes P, Ho M. Comparison of sleep disturbance in mild versus severe Parkinson's disease. Sleep 2002;25:573-577.

Yu B, Xiao ZY, Li JZ, Yuan J, Liu YM. Study of an integrated non-motor symptoms questionnaire for Parkinson's disease. Chin Med J (Engl) 2010;123:1436-1440.

Zahodne LB, Bernal-Pacheco O, Bowers D, et al. Are selective serotonin reuptake inhibitors associated with greater apathy in Parkinson's disease? J Neuropsychiatry Clin Neurosci 2012;24:326-330.

Zahodne LB, Marsiske M, Okun MS, Bowers D. Components of depression in Parkinson disease. J Geriatr Psychiatry Neurol 2012;25:131-137.

Zahodne LB, Marsiske M, Okun MS, Rodriguez RL, Malaty I, Bowers D. Mood and motor trajectories in Parkinson's disease: multivariate latent growth curve modeling. Neuropsychology 2012;26:71-80.

Zampieri M, de Souza EA. Locus of control, depression, and quality of life in Parkinson's Disease. J Health Psychol 2011;16:980-987.

Zhang JL, Yang JF, Chan P. No association between polymorphism of serotonin transporter gene and depression in Parkinson's disease in Chinese. Neurosci Lett 2009;455:155-158.

Zheng J, Sun S, Qiao X, Liu Y. Depression in patients with Parkinson's disease and the associated features. Journal of Huazhong University of Science and Technology Medical sciences = Hua zhong ke ji da xue xue bao Yi xue Ying De wen ban = Huazhong keji daxue xuebao Yixue Yingdewen ban 2009;29:725-728.

Ziropadja L, Stefanova E, Petrovic M, Stojkovic T, Kostic VS. Apathy and depression in Parkinson's disease: the Belgrade PD study report. Parkinsonism Relat Disord 2012;18:339-342.
